# Feeding Systems and Host Breeds Influence Ruminal Fermentation, Methane Production, Microbial Diversity and Metagenomic Gene Abundance

**DOI:** 10.3389/fmicb.2021.701081

**Published:** 2021-07-20

**Authors:** Rajaraman Bharanidharan, Chang Hyun Lee, Krishnaraj Thirugnanasambantham, Ridha Ibidhi, Yang Won Woo, Hong-Gu Lee, Jong Geun Kim, Kyoung Hoon Kim

**Affiliations:** ^1^Department of Agricultural Biotechnology, College of Agriculture and Life Sciences, Seoul National University, Seoul, South Korea; ^2^Cargill Agri Purina Inc., Technology Application Center, Pyeongchang, South Korea; ^3^Department of Ecofriendly Livestock Science, Institute of Green Bio Science and Technology, Seoul National University, Pyeongchang, South Korea; ^4^Pondicherry Centre for Biological Science and Educational Trust, Tamil Nadu, India; ^5^GN Food Ltd., R&D Team, Seoul, South Korea; ^6^Department of Animal Science and Technology, SangHa Life Science College, Konkuk University, Seoul, South Korea; ^7^Department of International Agricultural Technology, Graduate School of International Agricultural Technology, Seoul National University, Pyeongchang, South Korea

**Keywords:** Hanwoo, TMR, rumen, methane, metagenome, microbial network

## Abstract

Our previous research revealed the advantages of separate feeding (SF) systems compared to total mixed ration (TMR) in terms of ruminal methane (CH_4_) production. The purpose of this experiment was to confirm the advantage of SF as a nutritional strategy for CH_4_ mitigation, and to determine the effects of different feeding systems (TMR and SF) on the rumen microbiome and associated metagenome of two different breeds and on CH_4_ emissions. We randomly allocated four Holstein (305 ± 29 kg) and four Hanwoo steers (292 ± 24 kg) to two groups; the steers were fed a commercial concentrate with tall fescue (75:25) as TMR or SF, in a crossover design (two successive 22-day periods). Neither feeding systems nor cattle breeds had an effect on the total tract digestibility of nutrients. The TMR feeding system and Hanwoo steers generated significantly more CH_4_ (*P* < 0.05) and had a higher yield [g/d and g/kg dry matter intake (DMI)] compared to the SF system and Holstein steers. A larger rumen acetate:propionate ratio was observed for the TMR than the SF diet (*P* < 0.05), and for Hanwoo than Holstein steers (*P* < 0.001), clearly reflecting a shift in the ruminal H_2_ sink toward CH_4_ production. The linear discriminant analysis (LDA) effect size (LEfSe) revealed a greater abundance (α < 0.05 and LDA > 2.0) of operational taxonomic units (OTUs) related to methanogenesis for Hanwoo steers compared to Holstein steers. Kendall’s correlation analysis revealed wide variation of microbial co-occurrence patterns between feeding systems, indicating differential H_2_ thermodynamics in the rumen. A metagenome analysis of rumen microbes revealed the presence of 430 differentially expressed genes, among which 17 and 27 genes exhibited positive and negative associations with CH_4_ production, respectively (*P* < 0.001). A strong interaction between feeding system and breed was observed for microbial and metagenomic abundance. Overall, these results suggest that the TMR feeding system produces more CH_4_, and that Hanwoo cattle are higher CH_4_ emitters than SF diet and Holstein cattle, respectively. Interestingly, host-associated microbial interactions differed within each breed depending on the feeding system, which indicated that breed-specific feeding systems should be taken into account for farm management.

## Introduction

Some dietary interventions have the potential to reduce ruminal CH_4_ emissions with little negative impact on the animals. One strategy involves the use of different cattle feeding systems, such as separate feeding (SF) and total mixed ration (TMR) feeding with concentrate and forage. A review by Beigh et al. (2017) also clarified the advantages of TMR over conventional SF systems in ruminants. Compared to SF, TMR has a stabilising effect on rumen pH, and also has positive effects on dry matter intake (DMI), milk fat content (Phipps et al., 1984), nutrient use efficiency (Lailer et al., 2005), total milk production (Reddy and Reddy, 1983), and microbial protein synthesis (Liu et al., 2015) in dairy cattle. TMR feeding was also reported to have various advantages in beef production, in terms of precision, efficiency and convenience, which are believed to improve overall on-farm productivity (Moya et al., 2014). The practice of TMR feeding in beef cattle production has increased gradually, and is now used in about 20% of beef cattle farms in Korea. This has led to an increase in research on its effects on ruminal fermentation, animal performance and carcass quality in Hanwoo (Korean native cattle) steers (Lee et al., 2010; Kim et al., 2011; Chung et al., 2017). However, the effects of the TMR and SF systems on ruminal CH_4_ mitigation have not been studied extensively in either dairy or beef cattle. One early study compared TMR and SF silage-based diets for maintenance-level feeding of lactating Holstein cows, and found no differences in CH_4_ emissions between the feeding systems (Holter et al., 1977). Our previous studies of Holstein steers, with an average daily gain (ADG) of 0.65 kg, revealed higher CH_4_ emissions with the TMR than SF system (Lee et al., 2016; Bharanidharan et al., 2018). However, further studies are needed to validate the effects of these feeding systems on CH_4_ production in beef cattle.

The extensive livestock production system in Korea has contributed to an increase in atmospheric CH_4_ concentrations, with a reported 3.89 MtCO_2_ eq/y derived from enteric fermentation; this accounted for 50% of total CH_4_ emissions (7.81 MtCO_2_ eq/y) in Korea from the agricultural sector in 2018 (FAOSTAT, 2021). In the first quarter of 2021, Korea had around 3.33 million beef cattle, with Hanwoo accounting for 3.16 million head and Holstein accounting for the remainder (Korean Statistical Information Service (KOSIS), 2021). Studies have suggested that host breeds provide different environments for the microbial ecosystem in the rumen, and may influence the microbial composition and CH_4_ emissions (Duthie et al., 2017; De Mulder et al., 2018; Olijhoek et al., 2018; Islam et al., 2021). A comparison of the whole-genome sequence between Hanwoo and Holstein steers also revealed huge differences in the copy number variation regions (CNVR) between the two breeds (Choi et al., 2014), which could influence the rumen microbial composition (Benson et al., 2010). Furthermore, studies have provided direct evidence of the genetic influence of the host animal on CH_4_ production by ruminal microbes, thereby allowing the breeding of low-emitting animals through genetic selection (Roehe et al., 2016; Zhang et al., 2020). However, there have been no studies directly comparing CH_4_ emissions and rumen microbiomes between the Hanwoo and Holstein breeds with the diet held constant.

A better understanding of the contributions of Korean indigenous breeds to the microbial metagenome and variation in CH_4_ emissions will enable identification of metagenomic markers and the development of an optimal feeding system, both of which will reduce ruminal emissions. The present study was performed to clarify the effects of different feeding systems (TMR and SF), as well as differences in metabolites and microbiota, between the rumens of Hanwoo and Holstein steers, and their interactions with CH_4_ emissions, fermentation characteristics and the microbial metagenome using next-generation sequencing.

## Materials and Methods

All experimental animals were obtained from the animal farm of Seoul National University. The methods and protocols for all experiments involving animals were approved by the Committee for the Institutional Animal Care and Use of Seoul National University (SNU-160105-1), and performed in accordance with relevant guidelines and regulations.

### Experimental Animals and Diet

Four Holstein and four Hanwoo steers [initial body weights (BWs) of 305 ± 29 and 292 ± 24 kg, respectively] were allocated to two adjacent pens fitted with Calan doors. Four steers of each breed were offered commercial concentrate and tall fescue hay (75:25, on a DM basis) as either TMR or SF for two consecutive 22-day periods in a crossover design. All animals were adapted for 30 days to the experimental diet (TMR), and to the Calan doors, before the experiment. Each experimental period included 14 days of diet adaptation in the pen, 3 days of metabolic adaptation in a respiratory chamber, 2 days of CH_4_ measurements, 2 days of grab faecal sampling and 1 day of rumen fluid sampling. The diets (Table 1) were provided at 2.1% BW twice daily (at 09:00 and 18:00) and the animals had unrestricted access to water in both the pen and respiratory chamber. The DM intake was recorded daily, both in the pen and in the chamber. The TMR diet was prepared by blending commercial concentrate and chopped tall fescue hay every 7 days during the experimental period. The prepared TMR diet was then packed in vinyl bags in quantities of 20 kg and stored for feeding to the animals. In the SF system, tall fescue hay was provided first, followed by concentrate pellet with flaked corn 40 min later, to avoid an unnecessary drop in pH during initial ruminal fermentation compared to the TMR diet. The BW of the animals was measured at both the beginning and end of each period to determine the ADG. The offered feed samples were collected at the beginning of the experimental period. The refused feed samples were collected daily when the animals were placed in the chamber. Both samples were packed and stored at −20°C until being analysed for DM content and the composition of other chemicals as described previously (Bharanidharan et al., 2018). The particle size of the offered feed in both the TMR and SF diets was determined using a Penn State particle separator according to the technique of Kononoff et al. (2003).

**TABLE 1:** Ingredients and chemical composition of the basal diet used in the experiment.

**Ingredient composition, % DM**	
***Concentrate***	
Broken corn	1.0
Wheat	12.8
Sodium bicarbonate	0.6
Rice bran	5.0
Salt	0.2
Molasses	2.0
Ammonium chloride	0.1
CMS	1.1
Corn flake	15.0
DDGS	7.2
Soybean Hull	1.4
Amaferm^*TM*^	0.1
Corn gluten feed	15.0
Limestone	2.5
Palm kernel meal	11.0
Mineral-vitamin mixture^a^	0.2
***Roughage***	
Tall fescue hay	25.0
**Chemical composition, % DM**	
OM	93.3
CP	12.9
EE	4.4
aNDFom^b^	38.3
ADFom^c^	19.0
GE, MJ/kg	18.0

*CMS, condensed molasses soluble; DDGS, dried distiller’s grains with solubles.*

*Amaferm^*TM*^, fermentation extract of *Aspergillus oryzae* (Biozyme Enterprises Inc., MO, United States); CP, crude protein; EE, ether extract; GE, gross energy; OM, organic matter.*

*^*a*^Nutrients per kg of additive (Grobic-DC; Bayer HealthCare, Leverkusen, Germany): Vit. A, 2,650,000 IU; Vit. D3, 530,000 IU; Vit. E, 1,050 IU; Niacin, 10,000 mg; Mn, 4,400 mg; Zn, 4,400 mg; Fe, 13,200 mg; Cu, 2,200 mg; I, 440 mg; Co, 440 mg.*

*^*b*^Neutral detergent fibre assayed with a heat stable amylase and expressed exclusive of residual ash.*

*^c^Acid detergent fibre excluding residual ash.*

### Enteric CH_4_ Measurement, Digestion Trial, and Rumen Sampling

Methane production was measured using four open-circuit whole-body respiratory chambers, as described previously (Bharanidharan et al., 2018), with a ventilation rate of 700 L/min maintained throughout the experiment. The apparent total tract digestibility of nutrients in steers was studied using chromic oxide (Cr_2_O_3_) as an external marker, which was top-dressed twice daily at 0.2% of the daily feed amount throughout the experiment. On day 20, a faecal grab sample (100 g fresh weight) was collected from the rectum of each animal 30 min before feeding, and at 1, 3, 5, and 7 h post-feeding. On day 21, faeces were collected 30 min before feeding, and at 2, 4, 6, and 8 h post-feeding. Samples were frozen at −20°C until analysis. The samples were then composited by day and period for each steer prior to analysis of the digestibility of nutrients, as described previously (Bharanidharan et al., 2018). Ruminal fluid samples were collected before feeding (0 h), and at 1.5 and 3 h post-feeding, on day 22 of each period using a stomach tube (Oriental Dream, Hwaseong, Korea). The samples were filtered through four layers of muslin cloth. After immediately measuring pH using a Seven Easy pH meter (Mettler-Toledo, Schwerzenbach, Switzerland), ruminal fluid was centrifuged at 12,000 × g for 10 min (Smart 15; Hanil Science Industrial, Gimpo, South Korea). The supernatant was transferred to a 50-mL centrifuge tube and stored at −20°C for determination of ammonia-nitrogen (NH_3_-N) and volatile fatty acid (VFA) concentrations using the methods described previously (Bharanidharan et al., 2018). For microbial analysis, rumen fluid samples collected 3-h post-feeding were snap frozen in liquid nitrogen and then stored at −80°C until DNA extraction.

### 16SrRNA Gene Sequencing, Bioinformatics, and Statistical Analyses

The genomic DNA was extracted from 16 rumen samples (four per feeding system per breed) and a V4 sequencing library was constructed and sequenced for paired-end 250-bp reads using the Illumina MiSeq system (Illumina, San Diego, CA, United States). The primers and methods were described previously (Bharanidharan et al., 2018). The raw Illumina MiSeq reads were demultiplexed according to the barcodes and the sequences were quality-filtered based on the quality control process of Quantitative Insights Into Microbial Ecology (QIIME, version 1.9.0)^1^. The quality control conditions were as follows: read truncation: the raw read was truncated from the first low-quality base site with a maximum of three consecutive low-quality base calls (Phred Q < 20) allowed; and length filtering: to delete reads of continuous high quality (Phred *Q* ≥ 20), with a base length <70% of the read length. Non-overlapping regions, chimeric sequences and singletons were discarded. Each read was screened for operational taxonomic unit (OTU) picking using UCLUST embedded within QIIME 1.9.0, with reference to the Greengenes database (gg_otus-13_8-release, 97% nucleotide identity). The resulting OTU table was rarefied across samples to a depth of 10,000 reads based on the mean values of 10 iterations using the QIIME platform. All statistical analyses were performed on samples obtained from the same depth. Microbial community diversity was estimated in terms of the Chao1, Shannon, and Simpson indices using PAST software (Hammer et al., 2001). Determination of core and unique taxa was accomplished based on the abundance and prevalence (two-parameter) cut-offs across all the samples, using the inbuilt algorithm in Calypso software (Zakrzewski et al., 2017). Any taxa found to be ubiquitous (>90% occurrence across all samples) were defined as “core taxa” in the rumen, whereas those prevalent only in particular sample groups were defined as “unique taxa.” To identify bacterial lineages and other parameters that differentiated Hanwoo and Holstein cattle given the TMR or SF diet, we performed a principal component analysis (PCA) in the R software environment (R Development Core Team, Vienna, Austria) using the prcomp package; the results were visualised three-dimensionally using the pca3d package (Weiner, 2019). To identify robust microbial biomarkers for the two breeds, we calculated the linear discriminant analysis (LDA) effect size (LEfSe; Segata et al., 2011) using the Galaxy web application^2^. The OTU counts in each sample were used as the input for the LEfSe analysis. Differences among classes were determined using the Kruskal–Wallis test, at a significance level of *P* < 0.05 and with a threshold LDA score of 2.0. Feeding system was included as a subclass. The LEfSe subclasses were analysed using the Wilcoxon rank sum test, with *P* < 0.05 again taken to indicate significance. Relationships among microbes in the rumens of Hanwoo and Holstein steers given the TMR and SF diets were evaluated using Kendall’s correlation analysis, and a correlation plot was constructed using PAST software. To predict the molecular functions of each sample based on 16S rRNA data, we used the online version of the Phylogenetic Investigation of Communities by Reconstruction of Unobserved States (PICRUSt) bioinformatics software package (Langille et al., 2013), hosted on the Galaxy platform^2^. We used the Kyoto Encyclopaedia of Genes and Genomes (KEGG) database (Kanehisa and Goto, 2000) to predict the metagenome of our samples, based on OTU data obtained from rarefied 16S rRNA gene sequences using PICRUSt. The functions and products of the genes of interest were also obtained from the KEGG database. Differences between breeds given the TMR or SF diet in terms of the predicted molecular functions of bacterial communities were determined using a canonical correspondence analysis (CCA) in Calypso software. The non-parametric Kendall rank correlation coefficient was calculated to test the correlations among CH_4_ production, fermentation characteristics, bacterial communities, gene abundance and functional pathways in the rumen, using the corr.test function of the R psych package (Revelle, 2018). The resulting correlation matrix was visualised as a heatmap using the heatmap.2 function in the gplots R package (Warnes et al., 2015).

Daily CH_4_ emission, total tract digestibility, microbial diversity, gene abundance and functional abundance data were analysed using the MIXED procedure in SAS software (SAS Institute, Cary, NC, United States). The fixed effects in the model included breed, feeding system; their interaction effect was also analysed. Random effects, including period and animal, were nested within treatments. Ruminal fermentation characteristics were subjected to repeated measures analysis using the SAS PROC MIXED function (Littell et al., 1998). Appropriate covariance structures were obtained based on the Akaike information criterion. Means were calculated using the LSMEANS function, where each animal was considered as an experimental unit. *P* < 0.05 was taken to indicate significant differences according to treatment, and 0.05 < *P* < 0.1 was considered to indicate a trend toward significance.

## Results

### Effects of Feeding System and Host Breed on Enteric CH_4_ Production and Fermentation

The TMR diet had a higher percentage (99.5%) of particles <1.18 mm in size than the SF diet, whereas the SF diet had a higher percentage (72%) of particles in the size range of 19–8 mm than the TMR diet (*P* < 0.05) (Table 2). However, DM and organic matter (OM) intake did not vary between feeding systems (Table 3). Similarly, no feed sorting was observed in the SF diet. Holstein steers had higher DMI than Hanwoo steers in the chamber (Table 3). In addition, there were significant (*P* < 0.05) interactions between breed and feeding system for DM, OM, and gross energy (GE) intake (Table 3) in the chamber. Holstein steers had a greater (*P* < 0.05) ADG than Hanwoo steers (Table 3). Although there was no effect on apparent total tract nutrient digestibility (*P* > 0.05; Table 3), TMR feeding resulted in greater CH_4_ production (g/day) and CH_4_ yield per unit of OM, neutral detergent fibre and GE intake than the SF diet in both Holstein and Hanwoo steers (*P* < 0.05; Table 4). Hanwoo steers had a higher CH_4_ yield than Holstein steers (*P* < 0.005).

**TABLE 2:** Feed Particle size distribution (%) in different feeding systems.

**Particle size (% DM)**	**TMR**	**SF**	***P*-value**
>19 mm	13.8 ± 1.5	28.4 ± 1.9	0.034
19–8.0 mm	18.5 ± 1.2	64.5 ± 2.0	0.003
8.0–1.18 mm	29.1 ± 2.3	7.0 ± 0.4	0.004
<1.18 mm	38.6 ± 2.9	0.2 ± 0.6	0.004

*Values are least square means ± standard deviation (*n* = 3 replications).*

*DM, dry matter; SF, separate feeding; TMR, total mixed ration.*

**TABLE 3:** Least square means of body weight, nutrient intake and apparent total tract digestibility (%) of Hanwoo and Holstein steers fed by the TMR and SF systems.

**Item**	**Hanwoo**		**Holstein**		**SEM**	***P*-value**		
	**TMR**	**SF**	**TMR**	**SF**		**Breed**	**FS**	**Breed × FS**
Initial BW, kg	297.2	292.7	305.2	338.2	26.9	–	–	–
Final BW, kg	313.7	297.2	338.2	367.2	29.9	–	–	–
ADG, kg/d	0.6	0.6	1.1	1.0	0.26	0.014	0.411	0.591
***Intake (kg/d)***								
DM	5.7	5.1	6.4	7.6	0.65	0.093	0.401	0.026
OM	5.3	4.6	6.0	6.9	0.60	0.093	0.841	0.031
NDF	2.2	1.9	2.5	2.7	0.28	0.161	0.540	0.058
ADF	1.1	0.9	1.2	1.3	0.15	0.184	0.736	0.073
CP	0.8	0.7	0.9	1.1	0.09	0.067	0.008	0.013
GEI, MJ/d	103.4	100.1	116.4	149.2	12.50	0.088	0.028	0.015
***Digestibility (%)***								
DM	50.2	40.1	52.8	53.9	5.07	0.127	0.466	0.319
OM	54.1	41.3	55.9	52.3	6.29	0.310	0.169	0.399
NDF	37.6	37.9	39.9	44.3	4.69	0.437	0.677	0.717
ADF	30.2	32.0	30.9	41.8	5.06	0.391	0.304	0.459
CP	41.9	34.0	43.0	51.6	6.14	0.132	0.954	0.181
GE	56.1	44.5	58.1	55.0	5.93	0.325	0.125	0.322

*ADF, acid detergent fibre; ADG, average daily gain; BW, body weight; CP, crude protein; DM, dry matter; FS, feeding system; GEI, gross energy intake; NDF, neutral detergent fibre; OM, organic matter; SF, separate feeding; TMR, total mixed ration. Number of steers = 8.*

**TABLE 4:** Least square means of methane production recorded in Hanwoo and Holstein steers fed with TMR and SF diets over 24 h.

**Item**	**Hanwoo**		**Holstein**		**SEM**	***P*-value**		
	**TMR**	**SF**	**TMR**	**SF**		**Breed**	**FS**	**Breed × FS**
CH_4_, g/d	135.4	96.1	100.0	78.8	6.3	0.024	<0.0001	0.061
CH_4_, g/kg DMI	23.9	19.5	15.7	10.9	1.2	0.001	0.021	0.853
CH_4_, g/kg OMI	25.6	21.5	16.9	12.1	1.3	0.001	0.029	0.776
CH_4_, g/kg DOM	47.9	32.7	30.6	20.7	2.6	0.001	0.004	0.325
CH_4_, g/kg NDFI	62.4	55.2	41.1	30.5	4.5	0.003	0.091	0.680
CH_4_, g/kg DNDF	177.1	114.3	109.7	74.5	16.2	0.006	0.010	0.412
MCR	7.4	5.5	4.9	3.1	0.4	0.001	0.009	0.894
EF	49.4	35.1	36.5	28.8	2.3	0.024	<0.0001	0.074

*DMI, dry matter intake; DNDF, digestible NDF; DOM, digestible organic matter; EF, emission factor (CH_4_ kg/head/yr); FS, feeding system; GEI, gross energy intake; MCR, metabolic conversion ratio (CH_4_ energy expressed as %GEI); NDFI, neutral detergent fibre intake; OMI, organic matter intake; SF, separate feeding; TMR, total mixed ration. Number of steers = 8.*

The mean concentration of total ruminal VFA did not differ between the experimental treatments (*P* > 0.05; Table 5). The steers given the TMR diet had a lower ruminal pH than those given the SF diet (*P* = 0.069), and the proportion of acetate was higher in the rumen fluid of steers given the TMR diet (*P* < 0.01). The proportions of butyrate and isobutyric acid did not vary between feeding systems (*P* > 0.05), whereas the proportions of valerate and isovaleric acid were lower in steers given the TMR than SF diet (*P* = 0.001). Hanwoo steers had a lower propionate and higher butyrate content than Holstein steers (*P* < 0.001). Similarly, the ratio of acetate to propionate in Hanwoo steers was greater than that in Holstein steers (*P* < 0.05). Ruminal NH_3_-N concentrations did not vary significantly between feeding systems or breeds.

**TABLE 5:** Effects of feeding system and host breed on ruminal fermentation characteristics^a^.

**Item**	**Hanwoo**						**Holstein**						**SEM**	***P*-value**		
	**TMR**			**SF**			**TMR**			**SF**				**Breed**	**FS**	**Breed × FS**
	**0 h**	**1.5 h**	**3 h**	**0 h**	**1.5 h**	**3 h**	**0 h**	**1.5 h**	**3 h**	**0 h**	**1.5 h**	**3 h**				
pH	7.0	6.7	6.6	6.9	6.7	6.7	6.9	6.5	6.2	6.9	6.7	6.6	0.17	0.162	0.028	0.247
Total VFA, mM	86.7	110.6	112.3	81.4	103.6	101.6	80.7	110.1	105.7	55.3	108.9	113.6	8.51	0.457	0.168	0.888
**VFA,%**																
Acetate	46.8	43.0	44.5	44.8	42.4	43.5	46.5	42.0	42.8	44.5	38.1	37.9	1.57	0.210	0.001	0.073
Propionate	23.5	26.4	25.4	22.8	25.3	24.7	28.2	28.8	28.4	30.3	36.5	37.5	2.28	<0.0001	0.041	0.009
Isobutyrate	1.9	1.7	1.5	2.3	1.8	1.7	1.9	1.8	1.6	2.3	1.5	1.3	0.18	0.647	0.356	0.083
Butyrate	24.3	25.1	24.9	25.8	26.0	26.0	19.6	22.8	22.9	17.8	18.5	18.2	2.26	0.001	0.255	0.035
Isovalerate	2.0	1.9	1.7	2.7	2.5	2.1	2.4	2.7	2.3	3.0	2.6	2.0	0.22	0.051	0.001	0.006
Valerate	1.6	2.0	1.9	1.7	2.1	2.0	1.4	1.9	1.9	2.1	2.9	3.0	0.27	0.015	0.000	0.002
Acetate:propionate	2.0	1.6	1.8	2.0	1.7	1.8	1.7	1.5	1.5	1.5	1.1	1.1	0.13	<0.0001	0.042	0.021
NH_3_-N, mg/dL	7.8	18.1	13.4	10.6	17.6	11.5	6.9	19.3	13.2	4.0	11.4	8.9	2.45	0.096	0.081	0.068

*FS, feeding system; SF, separate feeding; TMR, total mixed ration.*

*a Values are least square means with standard error.*

*Number of steers = 8.*

### Effects of Feeding System and Host Breed on Rumen Bacterial/Archaeal Richness, Diversity, Composition, and Core Microbiome

**FIGURE 1:**
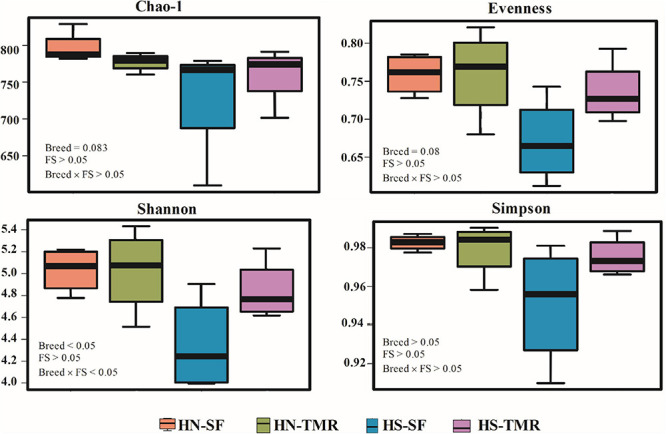
Alpha diversity metrics of the bacterial/archaeal composition in the rumen of Hanwoo (HN) and Holstein (HS) steers fed a total mixed ration (TMR) or separate feed (SF) diet.

At a depth of 10,000 quality reads, an average of 806 OTUs (range: 503–907 OTUs) were generated (Supplementary Figure 1). There were no differences (*P* > 0.05) in richness or diversity indices between the feeding systems. Differences in alpha diversity metrics, including observed OTUs (*P* < 0.05), Chao1 index (*P* = 0.083) and Shannon index (*P* < 0.05), were detected between breeds, demonstrating greater richness and diversity in Hanwoo steers (Figure 1 and Supplementary Table 1).

**FIGURE 2:**
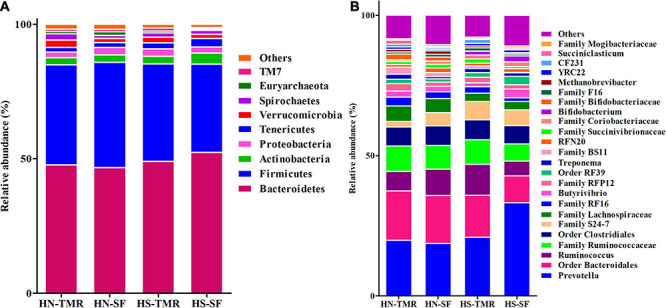
Taxonomic profiles of the relative phylum-level **(A)** and genus-level **(B)** abundances of bacteria and archaea in both Hanwoo (HN) and Holstein (HS) steers fed by different feeding systems, with a classification criterion of >0.5% of the total sequence.

The relative abundance of the bacterial genus *Prevotella* (18.6–33.2%) was high in all samples (Figure 2 and Supplementary Table 2). Similarly, the archaeal genus *Methanobrevibacter* (0.5–1.3%) was also abundant. The core microbiome across the different feeding systems and cattle breeds used in this study consisted of 49 identified and 37 unclassified genera, which together accounted for 98.74% of the total rumen microbial population (Supplementary Figure 2). When individual groups were analysed for core taxa, Holstein steers given the SF diet were found to possess a small, distinctive core microbiome, including three additional genera (*Acidaminococcus*, *Aequorivita*, and *B42*), which were found to be unique (Supplementary Table 3). Similarly, Acholeplasmatales unclassified and *Megasphaera* were identified as being unique to the Hanwoo steers and SF system, respectively.

**FIGURE 3:**
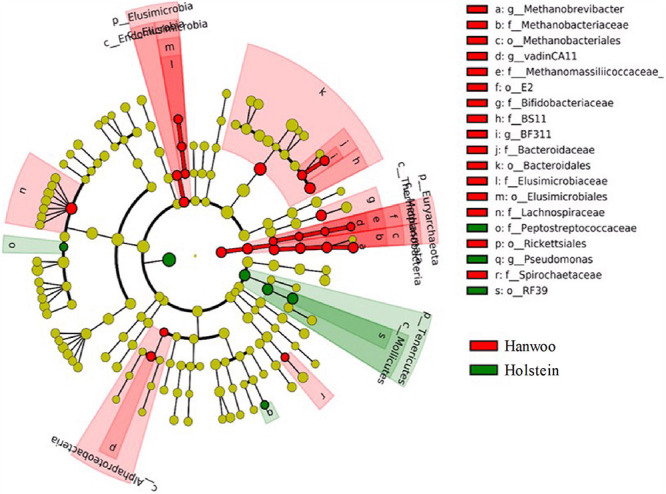
LDA effect size (LEfSe) cladogram demonstrating taxonomic differences between the Hanwoo and Holstein breeds. Taxa enriched in the rumen of Holstein steers are indicated by a positive LDA score (green), while taxa enriched in the rumen of Hanwoo steers have a negative score (red). Only taxa meeting an LDA significance threshold of 2 and *P* < 0.05 are shown.

The LEfSe analysis identified several OTUs that differed significantly between breeds (*P* < 0.05 and LDA > 2.0), indicating that they were robust biomarkers (Figure 3). The OTUs in the kingdom Archaea, phylum Euryarchaeota, families Methanomassiliicoccaceae and Methanobacteriaceae and the genera vadinCA11 and *Methanobrevibacter* were significantly more enriched in Hanwoo steers than Holstein steers. Several other families, including Spirochaetaceae, BS11 and Lachnospiraceae, were also identified as biomarkers in Hanwoo steers. A PCA biplot clustered all samples into two groups to explore the correlations with mean taxonomic annotation, CH_4_ yield and other fermentation parameters (Supplementary Figure 3).

### Effects of Feeding System and Host Breed on the Rumen Microbiome

Both feeding system and breed were found to have a significant effect on microbial composition at the phylum and genus levels (*P* < 0.05; Table 6). The feeding system × breed interaction effect on the rumen microbiome in relation to methanogenesis was significant (*P* < 0.05). Notably, the TMR system increased the archaea/bacteria ratio and abundances of the phylum Euryarchaeota and genus *Methanobrevibacter* in Hanwoo steers to a greater extent than in Holstein steers.

**TABLE 6:** Relative abundance of taxa in the Hanwoo and Holstein steers fed by different feeding systems.

**Classification**	**Total Sequences (%)**				**SEM**	***P*-Value**			**Function**
	**Hanwoo**		**Holstein**						
	**TMR**	**SF**	**TMR**	**SF**		**Breed**	**FS**	**Breed × FS**	
Archaea: Bacteria	0.010	0.014	0.007	0.005	0.003	0.022	0.787	0.041	
**Phylum level**									
Euryarchaeota	0.942	1.358	0.729	0.527	0.300	0.022	0.790	0.041	Hydrogenotrophic, H_2_ (U)
Fibrobacteres	0.270	0.222	0.610	0.208	0.117	0.188	0.079	0.156	Cellulolytic, acetate, formate, lactate (P)
Planctomycetes	0.191	0.480	0.136	0.247	0.069	0.062	0.016	0.221	NH_3_ oxidation, N_2_ (P)
Synergistetes	0.045	0.044	0.012	0.026	0.008	0.011	0.472	0.153	Saccharolytic
**Genus/family level**									
Ruminococcus	6.945	9.381	11.037	5.320	2.024	0.994	0.433	0.067	Cellulolytic, acetate, formate, lactate (P)
Coriobacteriaceae	0.518	1.046	1.395	1.615	0.352	0.093	0.401	0.639	Acetate and lactate (P)
Bifidobacteriaceae	2.063	1.412	0.038	0.082	0.555	0.057	0.392	0.335	Acetate and lactate (P)
Methanobrevibacter	0.874	1.297	0.697	0.500	0.292	0.025	0.773	0.046	Hydrogenotrophic, H_2_ (U)
CF231	1.026	0.618	1.367	0.264	0.382	0.975	0.256	0.094	Proteolytic, NH_3_ (P)
Succiniclasticum	1.370	0.485	0.330	0.461	0.299	0.099	0.225	0.113	Succinate (U), propionate (P)
Christensenellaceae	0.385	0.436	0.388	0.148	0.194	0.453	0.326	0.045	Fibre degrader
Fibrobacter	0.270	0.222	0.610	0.208	0.117	0.188	0.079	0.156	VFA (P)
p-75-a5	0.240	0.360	0.449	0.170	0.065	0.886	0.244	0.009	Fibrolytic
Pirellulaceae	0.191	0.480	0.135	0.247	0.070	0.061	0.016	0.222	NH_3_ oxidation, N_2_ (P)
Lactobacillus	0.056	0.580	0.112	0.079	0.116	0.060	0.240	0.031	Lactate (P)
BF311	0.289	0.254	0.127	0.088	0.077	0.053	0.625	0.979	Lignocellulose digestion
Selenomonas	0.406	0.081	0.107	0.042	0.081	0.060	0.035	0.133	Acetate, propionate, formate, lactate (P)
Bulleidia	0.031	0.044	0.028	0.506	0.153	0.062	0.420	0.048	Acetate, lactate, succinate (P)
Anaerovibrio	0.253	0.057	0.077	0.052	0.041	0.031	0.012	0.039	Succinate, propionate (P)
Desulfovibrio	0.126	0.065	0.051	0.112	0.039	0.676	0.990	0.086	Sulphur oxidation
Clostridiaceae	0.062	0.114	0.067	0.081	0.016	0.506	0.030	0.163	Glucose assimilation
Lachnospira	0.012	0.010	0.002	0.266	0.095	0.083	0.398	0.062	Acetate, formate, lactate (P)
Bacteroides	0.014	0.134	0.090	0.009	0.037	0.483	0.674	0.051	VFA (P)
Enterobacteriaceae	0.025	0.081	0.041	0.035	0.025	0.372	0.536	0.077	N_2_ fixation
Atopobium	0.020	0.046	0.026	0.046	0.008	0.738	0.020	0.717	Lactate (P)
Spirochaetaceae	0.053	0.042	0.028	0.008	0.016	0.014	0.053	0.535	Pectinolytic, Formate, acetate, lactate (P)
vadinCA11	0.052	0.040	0.015	0.013	0.007	0.007	0.420	0.472	Methylotrophic
Pseudomonas	0.011	0.018	0.018	0.070	0.015	0.025	0.282	0.030	Degrader
Dehalobacterium	0.031	0.052	0.026	0.007	0.024	0.400	0.910	0.078	H_2_ oxidation
Peptostreptococcaceae	0.003	0.007	0.030	0.074	0.023	0.081	0.476	0.104	N.I.
Pyramidobacter	0.033	0.030	0.010	0.026	0.008	0.059	0.528	0.092	Iso-fatty acids (P)
Weissella	0.016	0.043	0.014	0.016	0.008	0.122	0.030	0.050	Lactate (P)
Leuconostoc	0.012	0.026	0.009	0.009	0.009	0.373	0.429	0.041	Lactate (P)

*The table only includes taxa represented in >0.01% of the total sequences that tended to differ (0.05 < *P* < 0.1) and showed significant differences (*P* < 0.05).*

*FS, feeding system; N.I., no information; P, producer; SF, separate feeding; TMR, total mixed ration; U, utiliser; VFA, volatile fatty acids.*

*Data are least square means with standard error.*

*Number of steers = 8.*

### Co-occurrence of Rumen Microbes in Steers Varied by Feeding System and Host Breed

**FIGURE 4:**
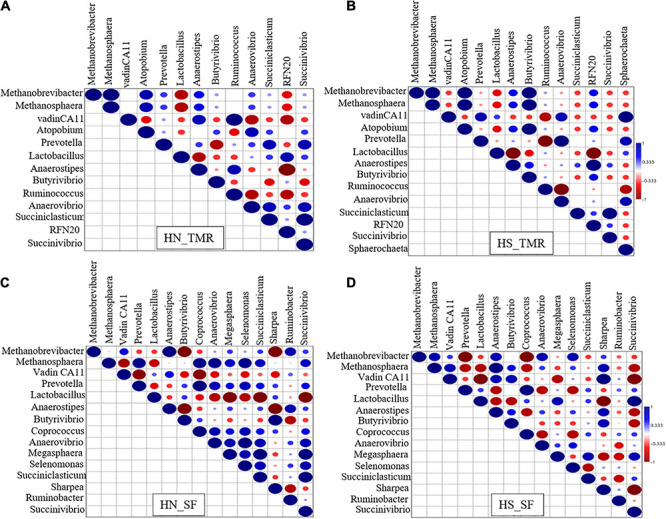
Kendall rank correlation analysis of the co-occurrences of microbial lineages. **(A)** Hanwoo (HN) steers fed a total mixed ration (TMR) diet. **(B)** Holstein (HS) steers given TMR diet. **(C)** Hanwoo (HN) steers given separate feed (SF) diet. **(D)** Holstein (HS) steers given SF diet. The genera were included in the matrix if they had a relative abundance of at least 0.01% in at least one steer. Correlation strength is indicated by the intensity of the colour. The scale colours denote whether the correlation is positive (closer to 1, blue Spheres) or negative (closer to −1, red Spheres). Big sphere indicates that the correlation is significant (*P* < 0.05).

Strong associations of hydrogenotrophic *Methanobrevibacter* and methylotrophic *Methanosphaera* with *Butyrivibrio* were detected in Holstein steers given the TMR diet (τ = 1, *P* < 0.05; Figure 4B). Strong associations were also detected between vadinCA11 and *Ruminococcus* (τ = 1, *P* < 0.05) in TMR-fed Hanwoo steers (Figure 4A), and between pectinolytic *Sphaerochaeta* and vadinCA11 (τ = 1, *P* < 0.05) in the TMR diet Holstein steers (Figure 4B). In contrast, in the SF Holstein steers (Figure 4D), we detected trends toward negative associations of *Methanobrevibacter* and vadinCA11 with *Anaerostipes*, *Coprococcus*, *Anaerovibrio*, *Prevotella*, *Pyramidobacter*, and *Succiniclasticum* (τ = −0.91, *P* = 0.063). In SF Hanwoo steers (Figure 4C), negative associations were observed between *Methanobrevibacter* and *Sharpea*, and between *Anaerostipes* and *Butyrivibrio* (τ = −1, *P* < 0.05).

### Predicted Molecular Functions of Steer Rumen Microbiota Varied by Feeding System and Host Breed

A total of 430 genes (Supplementary Table 4) and 49 gene families (Supplementary Table 5) differed between feeding systems or breeds (*P* < 0.1). The genes *pyruvate kinase* (*pyk*), K00873 (*P* < 0.05); *branched-chain amino acid transport system permease protein* (*livM*), K01998 (*P* < 0.05); *anaerobic carbon-monoxide dehydrogenase catalytic subunit* (*cooS*), K00198; and *F-type H^+^-transporting ATPase subunits* (*atpA–G*), K02110 (*P* = 0.053) were abundant in the SF system. *Pyruvate ferredoxin oxidoreductase alpha subunit* (*porA*), K00169 (*P* = 0.076) and *cobalt/nickel transport system permease protein* (*cbiM*), K02007 (*P* = 0.083) were enriched in Hanwoo steers.

**FIGURE 5:**
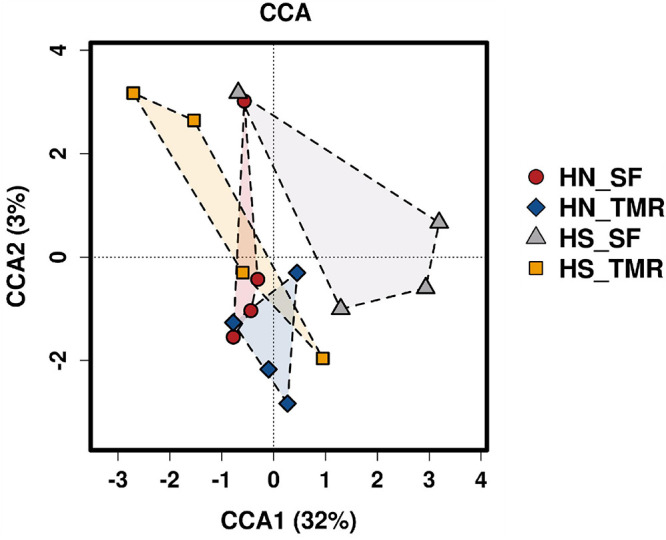
Canonical correspondence analysis (CCA) of microbial functional diversity in the rumen of Hanwoo (HN) and Holstein (HS) steers fed a total mixed ration (TMR) or separate feed (SF) diet.

**FIGURE 6:**
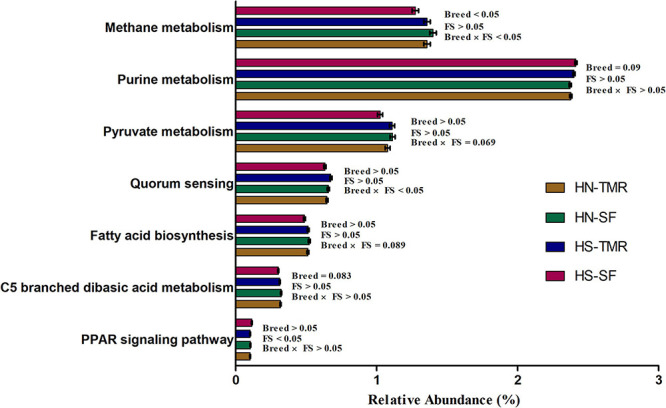
Variations in important rumen microbial Kyoto Encyclopaedia of Genes and Genomes (KEGG) metabolic pathways related to methane emissions in Hanwoo (HN) and Holstein (HS) steers fed a total mixed ration (TMR) or separate feed (SF) diet.

Pathways related to CH_4_ metabolism (*P* = 0.050) and butanoate metabolism (*P* < 0.05) were enriched in the rumen of Hanwoo steers, whereas those related to the purine metabolism pathway (*P* = 0.090) were abundant in Holstein steers. CCA of the relative abundance values of KEGG pathways of genes from the rumen microbiota showed distinct clustering of Hanwoo and Holstein cattle given the TMR and SF diets, explaining 32% of the variance (Figure 5). Genes related to CH_4_ metabolism (*P* = 0.050), pyruvate metabolism (*P* = 0.069), the bacterial secretion system (*P* < 0.05), and fatty acid biosynthesis (*P* = 0.089) were enriched in Hanwoo steers given the SF diet compared to Holstein steers given the SF diet (Figure 6).

### Association Between Microbial Gene Abundance and Steer Rumen CH_4_ Production Varied by Feeding System and Host Breed

**FIGURE 7:**
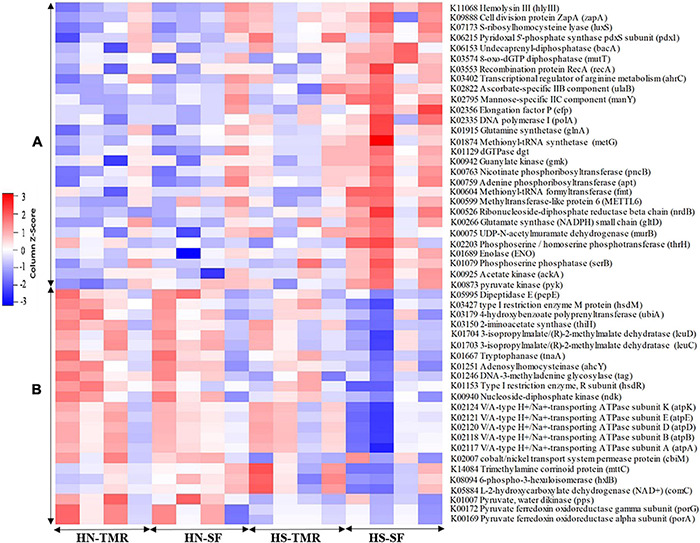
Heatmap of the relative abundance of microbial genes negatively **(A)** and positively **(B)** associated with methane production, as identified by Kendall rank correlation analysis. The scale colours denote whether the abundance is high (closer to 1, red squares) or low (closer to −1, blue squares).

**FIGURE 8:**
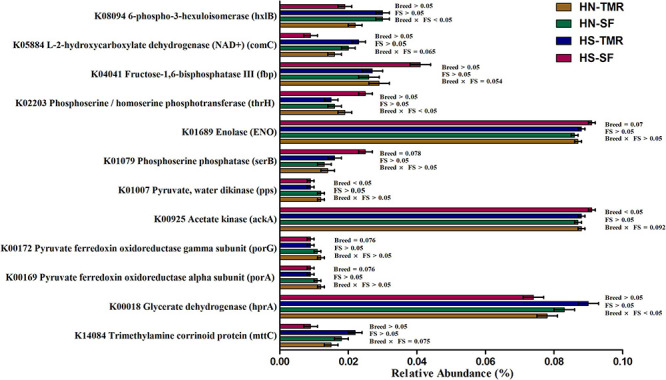
The metagenomic abundance of key genes involved in the methane metabolism pathway that tend to differ (0.05 < *P* < 0.1) or were significantly different (*P* < 0.05) between Hanwoo (HN) and Holstein (HS) steers fed a total mixed ration (TMR) or separate feed (SF) diet.

A total of 44 genes were strongly correlated with CH_4_ production, of which 17 were positively correlated and 27 were negatively correlated (Supplementary Table 6). The relative abundances of genes associated with CH_4_ production are presented as a heatmap, which clearly showed that most genes positively and negatively correlated with CH_4_ emissions were enriched in TMR Hanwoo and SF Holstein cattle, respectively (Figure 7). The genes *pyruvate ferredoxin oxidoreductase gamma subunit* (porG), K00172 (τ = 0.50, *P* = 0.006); *trimethylamine-corrinoid protein* (mttC), K14084 (τ = 0.76, *P* < 0.001); cbiM, K02007 (τ = 0.39, *P* = 0.004); *V/A-type H^+^/Na^+^-transporting ATPase subunit* (atpA), K02117 (τ = 0.47, *P* = 0.001); pyk, K00873 (τ = −0.41, *P* = 0.003); and *acetate kinase* (*ackA*), K00925 (τ = −0.46, *P* = 0.001) were found to be involved in pyruvate/CH_4_ metabolism, exhibiting significant associations with CH_4_ yield. Among these genes, porG (*P* = 0.076) and cbiM (*P* = 0.083) were enriched in Hanwoo steers (Figure 8).

### Association Among Rumen CH_4_ Production, Microbial Abundance, Functional Abundance, and Metabolites

**FIGURE 9:**
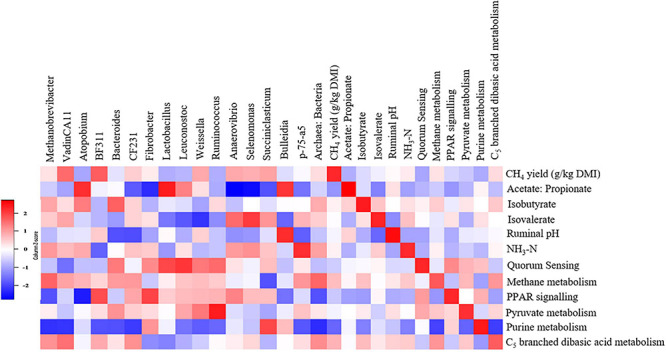
Associations among methane yield, fermentation parameters, rumen microbes, and metabolic pathways in steers. The genera and metabolic pathways were included in the matrix if they were differentially abundant (*P* < 0.1) and had at least 0.01% relative abundance in at least one of the steers. Correlation strength is indicated by the intensity of the colour. The scale colours denote whether the correlation is positive (closer to 1, red squares) or negative (closer to −1, blue squares).

We detected associations (*P* < 0.1) among several variables (Figure 9 and Supplementary Tables 7, 8). Notably, archaeal genera *Methanobrevibacter* (τ = 0.31, *P* = 0.094), vadinCA11 (τ = 0.41, *P* < 0.05) and the archaea*:*bacteria ratio (τ = 0.33, *P* = 0.078) were positively associated with the CH_4_ yield and CH_4_ metabolism pathway. *Fibrobacter* (τ = −0.46, *P* < 0.05), *Anaerovibrio* (τ = −0.51, *P* < 0.05), *Selenomonas* (τ = −0.59, *P* < 0.005) and *Succiniclasticum* (τ = −0.25, *P* = 0.077) were negatively associated with the acetate:propionate ratio in the rumen. The CH_4_ metabolism (τ = 0.43, *P* < 0.05), purine metabolism (Kendall’s τ = −0.42, *P* < 0.05) and C_5_ branched dibasic acid metabolism (τ = 0.47, *P* < 0.05) pathways were associated with CH_4_ yield.

## Discussion

Consistent with several previous reports, neither feeding system nor breed had any effect on total tract digestibility in the present study (Holter et al., 1977; Yan et al., 1998; Huuskonen et al., 2014). However, a recent study (Genís et al., 2021) reported increases in DM and CP digestibility in animals fed with the SF diet compared to the TMR diet using unprocessed long straw for which more than 70% of particles were >19 mm. However, this cannot be compared with the present study where the proportion of feed particles >19 mm was only 28%. Furthermore, in the present study, the ruminal pH of Holstein steer at 3 h post-TMR feeding was inconsistent with our previous study, which showed that the TMR diet helped to maintain ruminal pH (Bharanidharan et al., 2018). This may have been due to the 40-min delay in feeding of the concentrate to the SF animals, where the presence of sodium bicarbonate in the concentrate may have had a longer-duration stabilising effect on the rumen pH compared to TMR feeding. Furthermore, in the present study, rumen sampling was limited to 3 h, where the SF group may have experienced a further decrease in pH compared to the TMR diet in the later feeding period. In addition, in the TMR feeding system, feed particles <8 mm accounted for almost 70% of the total feed, which may have decreased the amount of time spent chewing and the ruminal pH during the initial phase of feeding (Krause et al., 2002). However, a recent study (Genís et al., 2021) noted no change in rumination or chewing activity between animals given the TMR and SF diets, although there were large variations in ruminal pH. Several studies have also reported no changes in ruminal pH in association with TMR feeding or feed particle size (Li et al., 2003; Schroeder et al., 2003; Liu et al., 2015).

Nutrition, feeding management, and animal production strategies for reducing enteric CH_4_ emissions may be more effective than strategies using feed additives as rumen modifiers (reviewed by Knapp et al., 2014). This is because a few rumen modifiers have been shown to be associated with adverse effects on rumen microbial fermentation, digestibility, residues in livestock products and adaptation to microbes on prolonged feeding at effective concentrations (reviewed by Honan et al., 2021). In this study, TMR feeding resulted in greater CH_4_ production and yield, consistent with the findings of our previous study (Bharanidharan et al., 2018). This may be related to the larger and smaller proportions of acetate and propionate, respectively, in the TMR diet, suggesting that there were fewer beneficial hydrogenotrophic reactions in the TMR system (Moss et al., 2000). Our observations contradicted earlier reports (reviewed by Janssen, 2010) that smaller particle sizes and higher passage rates were associated with a higher rumen H_2_ concentration, so that negative thermodynamic feedback reduces the production of H_2_ by fermentative microbes, resulting in increased propionate formation and ultimately reduced CH_4_ formation. Although the ruminal turnover rate was not evaluated in the present study, we postulated that the time lag between forage and concentrate feeding in the SF diet may have increased the feed passage rate compared to the TMR diet, thereby increasing propionate production and reducing CH_4_ production (Janssen, 2010). However, the limited effects of passage rate on total tract digestibility in the present study could have been due to post-ruminal compensatory digestion (Hoover, 1978). The increased proportions of isovaleric acid and valerate in the rumen of steers with the SF diet were consistent with the results of a previous study suggesting increased ruminal protein digestion (Allison and Bryant, 1963). The effects of iso-fatty acids on the synthesis of branched-chain amino acids and microbial protein depends on a wide range of hydrogenation and carboxylation reactions, which probably affect methanogenesis (Bharanidharan et al., 2018). Numerous studies have reported advantages of iso-fatty acids, in terms of rumen microbial status (Liu et al., 2014), microbial protein synthesis (Kim et al., 2005), lactation performance (Liu et al., 2018), and nutrient utilisation (Misra and Thakur, 2001). Molecular analysis of the rumen contents of feedlot beef steers also revealed increased valerate concentrations in animals with improved feed efficiency (Guan et al., 2008). However, the lack of difference in ADG between feeding systems suggested a need for more studies focusing on the effects of the SF system on animal performance.

The lower ADG observed in Hanwoo steers in the present study may have been related to the greater rumen microbial richness. An earlier study (Shabat et al., 2016) reported an association between greater microbial richness and inefficiency in cattle. Similarly, another study (Kang et al., 2005) suggested that Hanwoo steers may be inefficient when compared to Holstein steers, consistent with the results of this study. The robust rumen microbial biomarkers identified by LEfSe analysis in the present study indicated enrichment of several archaeal genera in Hanwoo steers compared to Holstein steers. This is consistent with a previous study (Lee et al., 2012), which reported a larger archaeal population in the rumen of Korean Hanwoo steers than Holstein dairy cows. We also detected a strong positive association of the archaea:bacteria ratio with CH_4_ yield, which is regarded as a proxy for CH_4_ emissions, regardless of cattle breed or diet (Wallace et al., 2015a; Auffret et al., 2018). In the present study, the most abundant archaeal taxa (*Methanobrevibacter*) was also positively associated with CH_4_ yield, consistent with previous reports of its higher abundance in high CH_4_ emitters (Wallace et al., 2015a; Auffret et al., 2018). The greater abundances of Methanomassiliicoccaceae and vadinCA11 in the rumen of Hanwoo steers indicated higher methylamine/methanol-mediated CH_4_ production (Paul et al., 2012). A previous study (Lee et al., 2012) also noted a higher concentration of methylamine in the rumen of Hanwoo steers than Holstein dairy cows. This was further supported by the higher abundances of pectinolytic Lachnospiraceae (Dusková and Marounek, 2001) and Spirochaetaceae (Ziołecki and Wojciechowicz, 1980), which may increase methylamine/methanol concentrations *via* the fermentation of digested pectin (Schink and Zeikus, 1980). Furthermore, this hypothesis was supported by the positive associations among vadinCA11, *Sphaerochaeta* and CH_4_ yield, and a recent study that revealed strong positive associations of *Sphaerochaeta* and BS11 with CH_4_ production (Difford et al., 2018).

Although a difference in microbial abundance was observed between breeds in this study, the feeding system did not significantly influence the major rumen microbiome. However, it did affect minor genera, such as *Atopobium* and *Weissella*, which are efficient lactate producers (Zhou et al., 2004; Abriouel et al., 2015) enriched in the animals given the SF diet. Enrichment of the *lactyl-CoA dehydrogenase* (*lcdA*) gene has been observed in *Megasphaera* spp., in low CH_4_ emitters that produce propionate from lactate *via* the acrylate pathway in the rumen (Counotte et al., 1981). Our core microbiome analysis only identified *Megasphaera* in steers given the SF diet, which may be related to their higher propionate content. Similarly, enrichment of lactate-producing *Sharpea* has been observed in the rumen of low CH_4_-emitting sheep (Kamke et al., 2016). Intriguingly, in the present study, a strong negative association was detected between *Sharpea* and *Methanobrevibacter* in Hanwoo steers given the SF diet, suggesting competitive utilisation of H_2_. Although increased CH_4_ production in the TMR system may be related to an increased ruminal H_2_ concentration, there were no differences in methanogen populations between the feeding systems. Similarly, the observed interaction between feeding system and breed for the total population of Euryarchaeota and *Methanobrevibacter* did not have a large influence on total CH_4_ yield. Many studies have failed to detect correlations between overall methanogen abundance and CH_4_ emissions (reviewed by Tapio et al., 2017). Therefore, further studies are needed to investigate other feeding variables, such as feeding behaviour (e.g., chewing activity), ruminal digestion and passage rate, which may provide a plausible explanation for the observed effects.

Despite the negligible differences in the microbiome, there were distinct microbial co-occurrences between the TMR and SF systems, which provided a plausible explanation for the observed differences in CH_4_ production. For example, the genera *Anaerostipes*, *Coprococcus*, *Anaerovibrio*, *Prevotella* and *Succiniclasticum*, which are involved in propionate production (Strobel, 1992; Van Gylswyk, 1995; Kishimoto et al., 2006; Privé et al., 2013; Reichardt et al., 2014), were negatively associated with hydrogenotrophic *Methanobrevibacter* in the Holstein steers given the SF diet, which clearly demonstrated the low availability of H_2_ for methanogenesis. The observed negative correlations of both *Anaerovibrio* and *Succiniclasticum* with the acetate:propionate ratio further supports this finding. *Megasphaera* and *Coprococcus* are more abundant in the rumen of efficient animals (Shabat et al., 2016), which may be related to the increased propionate and ADG in Holstein steers seen in the present study. Strong positive associations of the H_2_-producing bacteria *Ruminococcus* and *Butyrivibrio*, with vadinCA11 and *Methanobrevibacter*, respectively, were observed in the TMR feeding system, indicating that methanogenesis was the major H_2_ sink (Wolin et al., 1997).

Numerous studies have shown that microbes distributed in the rumen operate as an integrated system and have roles in the complex metabolic processes occurring within this ecosystem (Auffret et al., 2018; Wirth et al., 2018). The observed variations in H_2_ thermodynamics associated with changes in microbial abundances, and co-occurrences in breeds or feeding systems, reflect the predicted metabolic processes. The positive associations among the relative contents of isovalerate in the rumen, C_5_ branched dibasic acid metabolism pathway and CH_4_ metabolism pathway with CH_4_ yield clearly demonstrated a role of iso-fatty acids in CH_4_ production, as discussed in our previous report (Bharanidharan et al., 2018). The large abundance of genes encoding branched-chain amino acid transport system permease protein subclasses, *livH* (K01997) and *livM* (K01998) in the rumen of steers given the SF diet may have aided transport of branched-chain amino acids (Belitsky, 2015), in accordance with the increased and decreased iso-fatty acid and CH_4_ production in the rumen, respectively (Allison and Bryant, 1963). This hypothesis was supported by the associations among *Methanobrevibacter*, vadinCA11 and the C_5_ branched dibasic acid metabolism pathway in the rumen. Similarly, genes involved in pyruvate metabolism were strongly associated with the CH_4_ metabolism pathway, demonstrating their indirect roles in methanogenesis. The gene *ackA*, which produces acetyl phosphate by utilising acetate, and the genes *serB* and *thrH*, which produce serine by utilising H_2_, were negatively associated with CH_4_ production and enriched in low-emitting Holstein steers. In contrast, *porA* and *porG*, which are involved in acetyl-coA synthesis from pyruvate, leading to CO_2_ and H_2_ production, were positively associated with CH_4_ production and were abundant in high-emitting Hanwoo steers. These results clearly explain the differential patterns of pyruvate metabolism, H_2_ production and sinks between high- and low-emitting breeds detected in the present study. Similarly, Hanwoo steers showed abundant expression of cbiM, which helps to provide Ni to Ni-dependent methyl reductases and is correlated with methylamine/methanol-mediated CH_4_ production. Although no association was detected with CH_4_ yield, the gene *cooS*, which is involved in reductive acetogenesis (Wood et al., 1986), and *atpA–G*, which are involved in energy synthesis, were enriched in the SF system. All genes associated with methanogenesis in the present study were previously reported as potential metagenomic biomarkers of CH_4_ emission or feed efficiency (Wallace et al., 2015b; Roehe et al., 2016; Auffret et al., 2018; Lima et al., 2019), and could be used for the genetic selection of low-emitting animals. However, genes such as *methyl-coenzyme M reductase alpha subunit* (*mcrA*), *formylmethanofuran dehydrogenase subunit B* (*fmdB*) and *formate dehydrogenase alpha subunit* (*fdhF*), which are directly involved in the final step of methanogenesis leading to CH_4_ emission, were not identified in the present study. This could be attributed to the slightly higher (0.18 ± 0.04 SD) nearest sequence taxon index (NSTI) noted in the present study, thereby suggesting that functions based on 16S rRNA information have limitations, such that PICRUSt results should be interpreted with caution. Previous studies have also demonstrated weak correlations between the *mcr* gene and CH_4_ emissions (Morgavi et al., 2012; Tapio et al., 2017). Gene expression analyses can improve understanding of complex methanogenic processes compared to microbial and gene abundance analyses (Shi et al., 2014). A strong interaction was observed between breed and feeding system in the L-*fuculokinase* (*fucK*) gene, K00879, which is involved in fucose metabolism and plays an important role in host–microbe interactions (Hooper et al., 1999; Pacheco et al., 2012). Similarly, *Ruminococcus* spp., which are involved in fucose metabolism (Hooper et al., 2002) and correlated with quorum sensing and the CH_4_ metabolism pathway, also exhibited a similar feeding system × breed interaction. However, these interactions must be verified in a larger sample. Taken together, these results suggest that feeding systems differentially influence host–microbe interactions in different breeds, and therefore influence the rumen fermentation pattern and CH_4_ formation to some extent. Therefore, breed-specific feeding systems should be selected with caution.

## Conclusion

Comprehensive knowledge of bacterial/archaeal community composition and metabolic function is important for understanding their relationships with the host, and to develop feeding strategies to control CH_4_ emissions. In this study, we described the rumen microbial community and its associated functions in Holstein and Hanwoo steers provided with the same diet, under the same management conditions, to identify compositional changes that might underlie the marked differences in CH_4_ production between these breeds. This study is the first to show that Hanwoo cattle are higher CH_4_ emitters than Holstein steers, as supported by the greater abundance of bacterial/archaeal genera and genes involved in CH_4_ metabolism in Hanwoo cattle. We also demonstrated that, compared to TMR feeding, the SF system can reduce ruminal CH_4_ emissions, regardless of cattle breed. Similarly, genetic factors interacted with the feeding system, leading to divergent effects in the rumen, and therefore to large differences in microbial gene abundance, but with little effect on CH_4_ production. Wide variation in microbial co-occurrence patterns was observed in accordance with feeding system and breed, indicating different patterns of H_2_ thermodynamics in the rumen. The results of this study provide insight into the complex bacterial interactions occurring in the rumen, and may facilitate appropriate selection of strategies for modulating bacterial functions to reduce CH_4_ emissions. Specific microbial genes associated with CH_4_ can be used to develop molecular tools, facilitating the breeding of low CH_4_-emitting animals.

## Data Availability Statement

The datasets generated for this study can be found in online repositories. The name of the repository (NCBI) and accession number (PRJNA725944) can be found in the following link: https://www.ncbi.nlm.nih.gov/sra/PRJNA725944.

## Ethics Statement

The animal study was reviewed and approved by the Committee for the Institutional Animal Care and Use of Seoul National University (SNU-160105-1).

## Author Contributions

RB and KK designed and conceptualised the experiment. RB, CL, KT, RI, and YW performed the management of steers and sample collection. RB performed the operation of respiratory chamber, data curation, performed microbial data processing, bioinformatics, statistical analyses, visualisation, and wrote the first draft of the manuscript including tables and figures. RB, CL, and YW performed the laboratory analyses. RB, H-GL, JK, and KK revised the first draft of the manuscript including tables and figures. All authors contributed to the final manuscript revision, read and approved the final manuscript.

## Conflict of Interest

CL and YW are employed by Cargill Agri Purina Inc., and GN Food Ltd., respectively. The remaining authors declare that the research was conducted in the absence of any commercial or financial relationships that could be construed as a potential conflict of interest.

## Footnotes

1 http://qiime.org/index.html

2 https://huttenhower.sph.harvard.edu/galaxy
