# Supplementary material for: Feeding Systems and Host Breeds Influence Ruminal Fermentation, Methane Production, Microbial Diversity and Metagenomic Gene Abundance
Source: Front Microbiol. 2021 Jul 20;12:701081. doi: 10.3389/fmicb.2021.701081 (PMC8329423; doi:10.3389/fmicb.2021.701081)
Supplement: Supplementary file 1 [file Data_Sheet_1.docx]

***Supplementary data***

**Supplementary Table S1. Effect of different feeding system in Hanwoo and Holstein steers on rumen bacterial/archaeal richness and diversity.**

| **Diversity Measures** | **Hanwoo** | | **Holstein** | | **SEM** | ***P*-value** | | |
| --- | --- | --- | --- | --- | --- | --- | --- | --- |
|  | **TMR** | **SF** | **TMR** | **SF** |  | **Breed** | **FS** | **Breed × FS** |
| **No. of quality reads** | 86226.0 | 105771.0 | 79837.0 | 88100.0 | 16128.00 | 0.463 | 0.149 | 0.507 |
| **Observed OTUs^1^** | 858.0 | 857.0 | 832.0 | 676.0 | 46.69 | 0.008 | 0.340 | 0.029 |
| **Chao-1** | 960.5 | 977.4 | 926.3 | 770.3 | 53.73 | 0.015 | 0.390 | 0.074 |
| **Evenness** | 0.8 | 0.8 | 0.7 | 0.7 | 0.02 | 0.080 | 0.165 | 0.172 |
| **Shannon Index** | 5.1 | 5.1 | 4.9 | 4.4 | 0.19 | 0.040 | 0.146 | 0.142 |
| **Simpson Index** | 1.0 | 1.0 | 1.0 | 0.9 | 0.01 | 0.116 | 0.196 | 0.132 |

^1^ OTUs are based on the reads rarefied to the depth of 10000.

TMR, Total mixed ration; SF, separate feeding; FS, feeding system

Values are LS means with standard error. Number of steers = 8

**Supplementary Table S2. Relative abundance of taxa in the rumen of Hanwoo and Holstein steers fed by TMR or SF system representing > 0.1% of total sequences.**

| **Phylum/Classification** | **Total Sequences (%)** | | | | **SEM** | **P Value** | | |
| --- | --- | --- | --- | --- | --- | --- | --- | --- |
|  | **Hanwoo** | | **Holstein** | |  |  |  |  |
|  | **TMR** | **SF** | **TMR** | **SF** |  | **Breed** | **FS** | **Breed × FS** |
| ***Archaea: Bacteria*** | 0.010 | 0.014 | 0.007 | 0.005 | 0.003 | 0.022 | 0.787 | 0.041 |
| ***Euryarchaeota*** | 0.94 | 1.36 | 0.73 | 0.53 | 0.30 | 0.022 | 0.790 | 0.041 |
| Methanobrevibacter | 0.87 | 1.30 | 0.70 | 0.50 | 0.29 | 0.025 | 0.773 | 0.046 |
| ***Actinobacteria*** | 2.68 | 2.76 | 2.88 | 4.11 | 1.30 | 0.531 | 0.591 | 0.637 |
| Family Bifidobacteriaceae; Unclassified | 2.06 | 1.41 | 0.04 | 0.08 | 0.55 | 0.057 | 0.392 | 0.335 |
| Family Coriobacteriaceae; Unclassified | 0.52 | 1.05 | 1.39 | 1.62 | 0.35 | 0.093 | 0.401 | 0.639 |
| Bifidobacterium | 0.04 | 0.17 | 1.35 | 2.11 | 1.10 | 0.159 | 0.684 | 0.776 |
| ***Bacteroidetes*** | 47.75 | 46.70 | 48.98 | 52.35 | 4.13 | 0.420 | 0.784 | 0.603 |
| Prevotella | 19.79 | 18.63 | 20.88 | 33.24 | 5.50 | 0.159 | 0.304 | 0.220 |
| Order Bacteroidales; Unclassified | 17.59 | 17.11 | 14.98 | 9.50 | 2.39 | 0.036 | 0.145 | 0.211 |
| Family S24-7;  Unclassified | 2.01 | 4.75 | 6.55 | 5.64 | 1.63 | 0.120 | 0.576 | 0.278 |
| Family RF16;  Unclassified | 3.12 | 2.41 | 2.29 | 1.30 | 0.94 | 0.340 | 0.512 | 0.686 |
| Family BS11;  Unclassified | 2.49 | 1.40 | 0.93 | 0.17 | 0.72 | 0.133 | 0.320 | 0.778 |
| YRC22 | 0.88 | 0.44 | 0.91 | 1.06 | 0.29 | 0.281 | 0.613 | 0.328 |
| CF231 | 1.03 | 0.62 | 1.37 | 0.26 | 0.38 | 0.975 | 0.256 | 0.094 |
| Family Paraprevotellaceae; Unclassified | 0.35 | 0.31 | 0.53 | 0.28 | 0.10 | 0.428 | 0.154 | 0.309 |
| BF311 | 0.29 | 0.25 | 0.13 | 0.09 | 0.08 | 0.053 | 0.625 | 0.979 |
| Paludibacter | 0.08 | 0.41 | 0.19 | 0.05 | 0.18 | 0.474 | 0.674 | 0.218 |
| ***Elusimicrobia*** | 0.56 | 0.63 | 0.08 | 0.13 | 0.20 | 0.105 | 0.655 | 0.796 |
| Class Endomicrobia; Unclassified | 0.39 | 0.53 | 0.02 | 0.10 | 0.20 | 0.164 | 0.477 | 0.242 |
| ***Fibrobacteres*** | 0.27 | 0.22 | 0.61 | 0.21 | 0.12 | 0.188 | 0.079 | 0.156 |
| Fibrobacter | 0.27 | 0.22 | 0.61 | 0.21 | 0.12 | 0.188 | 0.079 | 0.156 |
| ***Firmicutes*** | 37.19 | 39.15 | 36.28 | 32.88 | 3.28 | 0.296 | 0.831 | 0.430 |
| Ruminococcus | 6.95 | 9.38 | 11.04 | 5.32 | 2.02 | 0.994 | 0.433 | 0.067 |
| Family Ruminococcaceae; Unclassified | 9.10 | 8.41 | 8.74 | 6.11 | 1.07 | 0.314 | 0.120 | 0.330 |
| Order Clostridiales; Unclassified | 6.72 | 7.02 | 7.11 | 6.50 | 1.14 | 0.950 | 0.917 | 0.662 |
| Family Lachnospiraceae; Unclassified | 5.56 | 5.03 | 2.91 | 2.98 | 1.20 | 0.111 | 0.848 | 0.801 |
| Butyrivibrio | 2.17 | 1.97 | 1.46 | 3.17 | 0.66 | 0.714 | 0.271 | 0.170 |
| RFN_2_0 | 0.98 | 1.60 | 1.00 | 1.33 | 0.66 | 0.862 | 0.487 | 0.828 |
| Succiniclasticum | 1.37 | 0.49 | 0.33 | 0.46 | 0.30 | 0.099 | 0.225 | 0.113 |
| Family Mogibacteriaceae; Unclassified | 0.51 | 0.62 | 0.48 | 0.65 | 0.10 | 0.968 | 0.294 | 0.698 |
| Sharpea | 0.03 | 0.43 | 0.09 | 1.30 | 0.31 | 0.196 | 0.033 | 0.213 |
| Clostridium | 0.37 | 0.47 | 0.35 | 0.42 | 0.13 | 0.805 | 0.515 | 0.931 |
| Family Christensenellaceae; Unclassified | 0.39 | 0.44 | 0.39 | 0.15 | 0.19 | 0.453 | 0.326 | 0.045 |
| Coprococcus | 0.17 | 0.20 | 0.15 | 0.83 | 0.28 | 0.160 | 0.420 | 0.122 |
| p-75-a5 | 0.24 | 0.36 | 0.45 | 0.17 | 0.06 | 0.886 | 0.244 | 0.009 |
| Anaerostipes | 0.52 | 0.29 | 0.14 | 0.23 | 0.21 | 0.219 | 0.789 | 0.248 |
| Family Veillonellaceae; Unclassified | 0.38 | 0.21 | 0.25 | 0.14 | 0.08 | 0.133 | 0.234 | 0.632 |
| Mogibacterium | 0.20 | 0.32 | 0.16 | 0.18 | 0.05 | 0.102 | 0.236 | 0.381 |
| Lactobacillus | 0.06 | 0.58 | 0.11 | 0.08 | 0.12 | 0.060 | 0.240 | 0.031 |
| Oscillospira | 0.19 | 0.25 | 0.18 | 0.15 | 0.04 | 0.245 | 0.443 | 0.143 |
| Moryella | 0.14 | 0.18 | 0.14 | 0.19 | 0.04 | 0.960 | 0.296 | 0.930 |
| Selenomonas | 0.41 | 0.08 | 0.11 | 0.04 | 0.08 | 0.060 | 0.035 | 0.133 |
| Bulleidia | 0.03 | 0.04 | 0.03 | 0.51 | 0.15 | 0.062 | 0.420 | 0.048 |
| Anaerovibrio | 0.25 | 0.06 | 0.08 | 0.05 | 0.04 | 0.031 | 0.012 | 0.039 |
| ***Lentisphaerae*** | 0.29 | 0.16 | 0.13 | 0.06 | 0.07 | 0.134 | 0.130 | 0.676 |
| Family Victivallaceae; Unclassified | 0.28 | 0.16 | 0.13 | 0.06 | 0.06 | 0.137 | 0.129 | 0.679 |
| ***Planctomycetes*** | 0.19 | 0.48 | 0.14 | 0.25 | 0.07 | 0.062 | 0.016 | 0.221 |
| Family Pirellulaceae; Unclassified | 0.19 | 0.48 | 0.14 | 0.25 | 0.07 | 0.061 | 0.016 | 0.222 |
| ***Proteobacteria*** | 2.00 | 2.78 | 2.70 | 2.21 | 1.18 | 0.925 | 0.933 | 0.384 |
| Family Succinivibrionaceae; Unclassified | 1.12 | 1.33 | 1.52 | 0.78 | 0.83 | 0.911 | 0.814 | 0.550 |
| Succinivibrio | 0.18 | 0.12 | 0.61 | 0.16 | 0.29 | 0.477 | 0.469 | 0.454 |
| Desulfobulbus | 0.08 | 0.23 | 0.04 | 0.19 | 0.09 | 0.500 | 0.360 | 0.971 |
| Order Rickettsiales; Unclassified | 0.07 | 0.39 | 0.01 | 0.02 | 0.18 | 0.321 | 0.468 | 0.373 |
| ***Spirochaetes*** | 2.26 | 1.07 | 1.56 | 1.39 | 0.55 | 0.703 | 0.191 | 0.318 |
| Treponema | 1.80 | 0.87 | 1.43 | 1.25 | 0.47 | 0.994 | 0.208 | 0.386 |
| Sphaerochaeta | 0.39 | 0.15 | 0.10 | 0.13 | 0.09 | 0.122 | 0.295 | 0.172 |
| ***TM7*** | 0.79 | 0.91 | 1.14 | 0.58 | 0.27 | 0.970 | 0.435 | 0.249 |
| Family F16;  Unclassified | 0.79 | 0.91 | 1.14 | 0.57 | 0.27 | 0.983 | 0.428 | 0.246 |
| ***Tenericutes*** | 1.79 | 1.77 | 2.23 | 3.21 | 0.40 | 0.106 | 0.126 | 0.118 |
| Order RF39;  Unclassified | 1.53 | 1.18 | 1.66 | 2.95 | 0.40 | 0.098 | 0.035 | 0.006 |
| Family Mycoplasmataceae; Unclassified | 0.12 | 0.43 | 0.44 | 0.21 | 0.17 | 0.733 | 0.777 | 0.099 |
| ***Verrucomicrobia*** | 2.76 | 1.62 | 2.15 | 1.57 | 0.89 | 0.585 | 0.171 | 0.640 |
| Family RFP12;  Unclassified | 2.62 | 1.44 | 1.97 | 1.51 | 0.87 | 0.599 | 0.163 | 0.519 |
| Family WCHB1-25; Unclassified | 0.11 | 0.13 | 0.16 | 0.04 | 0.04 | 0.763 | 0.248 | 0.139 |

Data is shown as LS Means with standard errors. Number of steers = 8

TMR, Total mixed ration; SF, separate feeding; FS, feeding system

**Supplementary Table S3. The abundance and prevalence of the core and unique microbiome identified in steers varied by breed and feeding system.**

| **Taxa** | **Abundance** | | | |  | **Prevalence** | | | |
| --- | --- | --- | --- | --- | --- | --- | --- | --- | --- |
|  | **Hanwoo** | | **Holstein** | |  | **Hanwoo** | | **Holstein** | |
|  | **TMR** | **SF** | **TMR** | **SF** |  | **TMR** | **SF** | **TMR** | **SF** |
| **Unique in HS steers fed by SF system** |  |  |  |  |  |  |  |  |  |
| Acidaminococcus | 0.000 | 0.002 | 0.000 | 0.115 |  | 0.00 | 0.25 | 0.00 | 0.75 |
| Aequorivita | 0.000 | 0.000 | 0.000 | 0.075 |  | 0.00 | 0.00 | 0.00 | 0.50 |
| B42 | 0.000 | 0.000 | 0.002 | 0.062 |  | 0.00 | 0.00 | 0.25 | 0.50 |
| **Unique in HN breed** |  |  |  |  |  |  |  |  |  |
| Unclassified Acholeplasmatales | 0.025 | 0.112 | 0.000 | 0.000 |  | 1.00 | 0.50 | 0.00 | 0.00 |
| **Unique in SF system** |  |  |  |  |  |  |  |  |  |
| Megasphaera | 0.025 | 0.012 | 0.000 | 0.040 |  | 0.25 | 0.75 | 0.00 | 0.75 |
| **Core Taxa** |  |  |  |  |  |  |  |  |  |
| Prevotella | 19.835 | 18.683 | 20.910 | 33.490 |  | 1.00 | 1.00 | 1.00 | 1.00 |
| Unclassified Bacteroidales | 17.593 | 17.107 | 14.977 | 9.505 |  | 1.00 | 1.00 | 1.00 | 1.00 |
| Ruminococcus | 6.945 | 9.385 | 11.040 | 5.322 |  | 1.00 | 1.00 | 1.00 | 1.00 |
| Unclassified Ruminococcaceae | 9.098 | 8.408 | 8.742 | 6.103 |  | 1.00 | 1.00 | 1.00 | 1.00 |
| Unclassified Clostridiales | 6.723 | 7.022 | 7.115 | 6.505 |  | 1.00 | 1.00 | 1.00 | 1.00 |
| Unclassified S247 | 2.007 | 4.748 | 6.550 | 5.643 |  | 1.00 | 1.00 | 1.00 | 1.00 |
| Unclassified Lachnospiraceae | 5.555 | 5.027 | 2.907 | 2.982 |  | 1.00 | 1.00 | 1.00 | 1.00 |
| Unclassified RF16 | 3.118 | 2.413 | 2.288 | 1.300 |  | 1.00 | 1.00 | 1.00 | 1.00 |
| Butyrivibrio | 2.170 | 1.968 | 1.455 | 3.170 |  | 1.00 | 1.00 | 1.00 | 1.00 |
| Unclassified RFP12 | 2.622 | 1.440 | 1.968 | 1.507 |  | 1.00 | 1.00 | 1.00 | 1.00 |
| Unclassified RF39 | 1.528 | 1.180 | 1.660 | 2.950 |  | 1.00 | 1.00 | 1.00 | 1.00 |
| Treponema | 1.805 | 0.872 | 1.432 | 1.248 |  | 1.00 | 1.00 | 1.00 | 1.00 |
| Unclassified BS11 | 2.493 | 1.400 | 0.928 | 0.168 |  | 1.00 | 1.00 | 1.00 | 1.00 |
| RFN_2_0 | 0.975 | 1.598 | 0.998 | 1.330 |  | 1.00 | 1.00 | 1.00 | 1.00 |
| Unclassified Succinivibrionaceae | 1.117 | 1.328 | 1.520 | 0.780 |  | 1.00 | 1.00 | 1.00 | 1.00 |
| Unclassified Coriobacteriaceae | 0.520 | 1.048 | 1.395 | 1.615 |  | 1.00 | 1.00 | 1.00 | 1.00 |
| Bifidobacterium | 0.042 | 0.178 | 1.355 | 2.110 |  | 1.00 | 1.00 | 1.00 | 1.00 |
| Unclassified Bifidobacteriaceae | 2.062 | 1.412 | 0.040 | 0.082 |  | 1.00 | 1.00 | 1.00 | 1.00 |
| Unclassified F16 | 0.792 | 0.910 | 1.143 | 0.570 |  | 1.00 | 1.00 | 1.00 | 1.00 |
| Methanobrevibacter | 0.875 | 1.297 | 0.698 | 0.500 |  | 1.00 | 1.00 | 1.00 | 1.00 |
| YRC22 | 0.880 | 0.432 | 0.910 | 1.055 |  | 1.00 | 1.00 | 1.00 | 1.00 |
| CF231 | 1.025 | 0.618 | 1.367 | 0.265 |  | 1.00 | 1.00 | 1.00 | 1.00 |
| Succiniclasticum | 1.367 | 0.485 | 0.332 | 0.462 |  | 1.00 | 1.00 | 1.00 | 1.00 |
| Unclassified Mogibacteriaceae | 0.515 | 0.615 | 0.482 | 0.658 |  | 1.00 | 1.00 | 1.00 | 1.00 |
| Sharpea | 0.030 | 0.425 | 0.090 | 1.305 |  | 1.00 | 1.00 | 1.00 | 1.00 |
| Clostridium | 0.378 | 0.475 | 0.365 | 0.448 |  | 1.00 | 1.00 | 1.00 | 1.00 |
| Unclassified Paraprevotellaceae | 0.352 | 0.308 | 0.528 | 0.282 |  | 1.00 | 1.00 | 1.00 | 1.00 |
| Unclassified Christensenellaceae | 0.388 | 0.438 | 0.390 | 0.145 |  | 1.00 | 1.00 | 1.00 | 1.00 |
| Coprococcus | 0.168 | 0.200 | 0.155 | 0.830 |  | 1.00 | 1.00 | 1.00 | 1.00 |
| Fibrobacter | 0.268 | 0.222 | 0.610 | 0.208 |  | 1.00 | 1.00 | 1.00 | 1.00 |
| p75a5 | 0.238 | 0.362 | 0.448 | 0.170 |  | 1.00 | 1.00 | 1.00 | 1.00 |
| Unclassified Mycoplasmataceae | 0.115 | 0.430 | 0.440 | 0.210 |  | 1.00 | 1.00 | 1.00 | 1.00 |
| Anaerostipes | 0.522 | 0.285 | 0.135 | 0.230 |  | 1.00 | 1.00 | 1.00 | 1.00 |
| Succinivibrio | 0.180 | 0.120 | 0.608 | 0.160 |  | 1.00 | 1.00 | 1.00 | 1.00 |
| Unclassified Pirellulaceae | 0.192 | 0.480 | 0.135 | 0.245 |  | 1.00 | 1.00 | 1.00 | 1.00 |
| Unclassified Endomicrobia | 0.390 | 0.528 | 0.022 | 0.103 |  | 1.00 | 1.00 | 1.00 | 0.75 |
| Unclassified Veillonellaceae | 0.380 | 0.213 | 0.250 | 0.143 |  | 1.00 | 1.00 | 1.00 | 1.00 |
| Mogibacterium | 0.202 | 0.322 | 0.158 | 0.175 |  | 1.00 | 1.00 | 1.00 | 1.00 |
| Lactobacillus | 0.055 | 0.578 | 0.112 | 0.080 |  | 1.00 | 1.00 | 1.00 | 1.00 |
| Sphaerochaeta | 0.390 | 0.152 | 0.098 | 0.130 |  | 1.00 | 1.00 | 1.00 | 1.00 |
| Oscillospira | 0.185 | 0.252 | 0.178 | 0.152 |  | 1.00 | 1.00 | 1.00 | 1.00 |
| BF311 | 0.288 | 0.255 | 0.128 | 0.088 |  | 1.00 | 1.00 | 1.00 | 1.00 |
| Paludibacter | 0.082 | 0.410 | 0.190 | 0.045 |  | 1.00 | 1.00 | 1.00 | 0.75 |
| Unclassified YS2 | 0.252 | 0.115 | 0.245 | 0.062 |  | 1.00 | 1.00 | 1.00 | 1.00 |
| Moryella | 0.143 | 0.182 | 0.143 | 0.188 |  | 1.00 | 1.00 | 1.00 | 1.00 |
| Selenomonas | 0.408 | 0.080 | 0.108 | 0.042 |  | 1.00 | 1.00 | 1.00 | 1.00 |
| Unclassified Victivallaceae | 0.278 | 0.160 | 0.130 | 0.060 |  | 1.00 | 1.00 | 1.00 | 0.75 |
| Bulleidia | 0.032 | 0.048 | 0.028 | 0.507 |  | 1.00 | 1.00 | 1.00 | 1.00 |
| Desulfobulbus | 0.085 | 0.235 | 0.040 | 0.185 |  | 1.00 | 1.00 | 1.00 | 1.00 |
| Unclassified Rickettsiales | 0.070 | 0.390 | 0.012 | 0.017 |  | 1.00 | 1.00 | 0.75 | 0.50 |
| Unclassified WCHB125 | 0.110 | 0.125 | 0.162 | 0.042 |  | 1.00 | 1.00 | 1.00 | 0.75 |
| Anaerovibrio | 0.252 | 0.055 | 0.078 | 0.053 |  | 1.00 | 1.00 | 1.00 | 0.75 |
| Desulfovibrio | 0.128 | 0.062 | 0.050 | 0.110 |  | 1.00 | 1.00 | 1.00 | 1.00 |
| Shuttleworthia | 0.038 | 0.025 | 0.100 | 0.180 |  | 0.75 | 1.00 | 1.00 | 1.00 |
| Blautia | 0.038 | 0.065 | 0.025 | 0.208 |  | 1.00 | 1.00 | 1.00 | 1.00 |
| Unclassified Clostridiaceae | 0.062 | 0.115 | 0.065 | 0.082 |  | 1.00 | 1.00 | 1.00 | 1.00 |
| Unclassified SR1 | 0.100 | 0.022 | 0.022 | 0.178 |  | 1.00 | 1.00 | 0.75 | 1.00 |
| Unclassified Elusimicrobiaceae | 0.130 | 0.098 | 0.057 | 0.025 |  | 1.00 | 1.00 | 1.00 | 0.75 |
| Bacteroides | 0.012 | 0.135 | 0.092 | 0.005 |  | 0.75 | 1.00 | 1.00 | 0.50 |
| Anaeroplasma | 0.085 | 0.032 | 0.100 | 0.008 |  | 1.00 | 1.00 | 1.00 | 0.50 |
| Eubacterium | 0.008 | 0.010 | 0.005 | 0.202 |  | 0.75 | 1.00 | 0.50 | 1.00 |
| L7A_E11 | 0.058 | 0.068 | 0.062 | 0.028 |  | 1.00 | 1.00 | 1.00 | 0.75 |
| Unclassified Pseudomonadaceae | 0.015 | 0.012 | 0.012 | 0.172 |  | 1.00 | 1.00 | 1.00 | 1.00 |
| Unclassified Alphaproteobacteria | 0.075 | 0.062 | 0.035 | 0.018 |  | 1.00 | 1.00 | 1.00 | 1.00 |
| Unclassified Comamonadaceae | 0.032 | 0.053 | 0.038 | 0.065 |  | 1.00 | 1.00 | 1.00 | 1.00 |
| Unclassified WPS2 | 0.032 | 0.062 | 0.035 | 0.055 |  | 0.75 | 0.75 | 1.00 | 0.50 |
| Unclassified Enterobacteriaceae | 0.025 | 0.080 | 0.040 | 0.035 |  | 1.00 | 1.00 | 1.00 | 1.00 |
| Atopobium | 0.018 | 0.048 | 0.028 | 0.045 |  | 1.00 | 1.00 | 1.00 | 1.00 |
| Unclassified Spirochaetaceae | 0.050 | 0.040 | 0.025 | 0.008 |  | 1.00 | 1.00 | 1.00 | 0.50 |
| vadinCA11 | 0.053 | 0.038 | 0.015 | 0.012 |  | 1.00 | 1.00 | 0.75 | 0.75 |
| Pseudomonas | 0.010 | 0.015 | 0.018 | 0.070 |  | 1.00 | 1.00 | 1.00 | 1.00 |
| Erwinia | 0.010 | 0.048 | 0.032 | 0.022 |  | 1.00 | 1.00 | 1.00 | 1.00 |
| Unclassified Streptophyta | 0.015 | 0.040 | 0.025 | 0.028 |  | 1.00 | 1.00 | 1.00 | 0.75 |
| Pyramidobacter | 0.035 | 0.030 | 0.012 | 0.028 |  | 1.00 | 1.00 | 1.00 | 0.75 |
| SMB53 | 0.005 | 0.018 | 0.020 | 0.060 |  | 0.50 | 1.00 | 0.75 | 1.00 |
| Unclassified Xanthomonadaceae | 0.008 | 0.022 | 0.012 | 0.057 |  | 0.75 | 1.00 | 1.00 | 0.75 |
| Streptococcus | 0.018 | 0.012 | 0.055 | 0.012 |  | 1.00 | 1.00 | 0.75 | 0.75 |
| Parabacteroides | 0.005 | 0.062 | 0.015 | 0.010 |  | 0.50 | 1.00 | 0.50 | 0.50 |
| Unclassified GMD14H09 | 0.030 | 0.017 | 0.012 | 0.032 |  | 0.75 | 0.75 | 0.75 | 0.75 |
| Weissella | 0.015 | 0.042 | 0.012 | 0.018 |  | 1.00 | 1.00 | 1.00 | 1.00 |
| Acinetobacter | 0.000 | 0.022 | 0.030 | 0.020 |  | 0.75 | 1.00 | 1.00 | 1.00 |
| Sphingomonas | 0.015 | 0.022 | 0.015 | 0.020 |  | 1.00 | 1.00 | 1.00 | 0.75 |
| Methanosphaera | 0.015 | 0.018 | 0.015 | 0.015 |  | 1.00 | 1.00 | 1.00 | 1.00 |
| Unclassified Prevotellaceae | 0.023 | 0.022 | 0.008 | 0.008 |  | 0.75 | 1.00 | 0.50 | 0.75 |
| Leuconostoc | 0.012 | 0.028 | 0.010 | 0.010 |  | 0.75 | 1.00 | 1.00 | 0.75 |
| Agrobacterium | 0.012 | 0.012 | 0.008 | 0.025 |  | 1.00 | 1.00 | 0.75 | 1.00 |
| **Other Shared Taxa** |  |  |  |  |  |  |  |  |  |
| Unclassified p253418B5 | 0.002 | 0.040 | 0.055 | 0.250 |  | 0.25 | 1.00 | 0.50 | 0.50 |
| Lachnospira | 0.012 | 0.010 | 0.002 | 0.265 |  | 1.00 | 1.00 | 0.25 | 0.75 |
| SHD231 | 0.035 | 0.085 | 0.045 | 0.108 |  | 1.00 | 0.75 | 1.00 | 0.25 |
| Lachnobacterium | 0.005 | 0.002 | 0.002 | 0.218 |  | 0.50 | 0.25 | 0.25 | 0.50 |
| Ruminobacter | 0.053 | 0.045 | 0.040 | 0.002 |  | 1.00 | 1.00 | 1.00 | 0.25 |
| Pseudoramibacter_Eubacterium | 0.028 | 0.025 | 0.000 | 0.078 |  | 0.50 | 0.75 | 0.00 | 0.75 |
| Dehalobacterium | 0.032 | 0.050 | 0.025 | 0.005 |  | 1.00 | 1.00 | 1.00 | 0.25 |
| Unclassified Peptostreptococcaceae | 0.002 | 0.005 | 0.030 | 0.073 |  | 0.25 | 0.50 | 1.00 | 1.00 |
| Unclassified ML615J28 | 0.025 | 0.012 | 0.028 | 0.030 |  | 1.00 | 0.75 | 0.75 | 0.25 |
| Luteimonas | 0.002 | 0.008 | 0.008 | 0.070 |  | 0.25 | 0.50 | 0.75 | 1.00 |
| Unclassified LD1 | 0.065 | 0.015 | 0.008 | 0.000 |  | 0.75 | 0.75 | 0.50 | 0.00 |
| Unclassified Porphyromonadaceae | 0.018 | 0.012 | 0.000 | 0.055 |  | 0.50 | 0.50 | 0.00 | 1.00 |
| Corynebacterium | 0.002 | 0.008 | 0.012 | 0.053 |  | 0.25 | 0.75 | 1.00 | 0.75 |
| Turicibacter | 0.000 | 0.005 | 0.012 | 0.042 |  | 0.00 | 0.50 | 0.50 | 0.75 |
| Unclassified Desulfovibrionaceae | 0.015 | 0.015 | 0.015 | 0.005 |  | 1.00 | 0.75 | 0.75 | 0.25 |
| Pediococcus | 0.000 | 0.030 | 0.010 | 0.002 |  | 0.00 | 0.75 | 0.50 | 0.25 |
| Phascolarctobacterium | 0.005 | 0.020 | 0.005 | 0.012 |  | 0.25 | 1.00 | 0.50 | 0.50 |
| Campylobacter | 0.008 | 0.002 | 0.010 | 0.018 |  | 0.75 | 0.25 | 0.75 | 0.75 |

TMR, Total mixed ration; SF, separate feeding; FS, feeding system

Number of steers = 8

**Supplementary Table S4. Relative abundance of rumen microbial genes in the Hanwoo and Holstein steers fed by two feeding system representing > 0.01% of total predicted genes by PICRUSt that tend to differ (0.05 < P < 0.1) and significantly differ (P < 0.05)**

| **KEGG ID** | **Gene Label** | **Gene definition** | **Hanwoo** | | **Holstein** | | **SEM** | **P Value** | | |
| --- | --- | --- | --- | --- | --- | --- | --- | --- | --- | --- |
|  |  |  | **TMR** | **SF** | **TMR** | **SF** |  | **Breed** | **FS** | **Breed × FS** |
| K01990 | ABC-2.A | ABC-2 type transport system ATP-binding protein | 0.272 | 0.263 | 0.263 | 0.285 | 0.005 | 0.380 | 0.370 | **0.047** |
| K00266 | gltD | glutamate synthase (NADPH) small chain [EC:1.4.1.13] | 0.243 | 0.239 | 0.247 | 0.261 | 0.004 | **0.056** | 0.450 | 0.180 |
| **K07024** | **SPP** | **sucrose-6-phosphatase [EC:3.1.3.24]** | 0.212 | 0.230 | 0.226 | 0.259 | 0.008 | 0.130 | **0.079** | 0.550 |
| K03497 | spoJ | chromosome partitioning protein ParB family | 0.196 | 0.208 | 0.211 | 0.180 | 0.006 | 0.530 | 0.360 | **0.046** |
| K11754 | folC | dihydrofolate synthase / folylpolyglutamate synthase  [EC:6.3.2.12 6.3.2.17] | 0.177 | 0.175 | 0.175 | 0.184 | 0.002 | 0.210 | 0.280 | **0.052** |
| **K00599** | **METTL6** | **methyltransferase-like protein 6 [EC:2.1.1.-]** | 0.169 | 0.177 | 0.171 | 0.186 | 0.003 | 0.220 | **0.020** | 0.450 |
| K03655 | recG | ATP-dependent DNA helicase RecG [EC:3.6.4.12] | 0.178 | 0.174 | 0.176 | 0.171 | 0.001 | 0.200 | **0.051** | 0.910 |
| K02078 | acpP | acyl carrier protein | 0.171 | 0.168 | 0.175 | 0.180 | 0.002 | **0.082** | 0.730 | 0.360 |
| K11753 | ribF | riboflavin kinase / FMN adenylyltransferase [EC:2.7.1.26 2.7.7.2] | 0.169 | 0.169 | 0.172 | 0.173 | 0.001 | **0.094** | 0.640 | 0.720 |
| K01534 | zntA | Cd2+/ZN_2_+-exporting ATPase [EC:3.6.3.3 3.6.3.5] | 0.151 | 0.151 | 0.155 | 0.162 | 0.002 | **0.081** | 0.420 | 0.320 |
| K01915 | glnA | glutamine synthetase [EC:6.3.1.2] | 0.149 | 0.151 | 0.153 | 0.160 | 0.002 | **0.086** | 0.160 | 0.430 |
| K01703 | leuC | 3-isopropylmalate/(R)-2-methylmalate dehydratase large subunit [EC:4.2.1.33 4.2.1.35] | 0.157 | 0.157 | 0.152 | 0.144 | 0.003 | **0.057** | 0.320 | 0.320 |
| K01704 | leuD | 3-isopropylmalate/(R)-2-methylmalate dehydratase small subunit [EC:4.2.1.33 4.2.1.35] | 0.153 | 0.153 | 0.150 | 0.141 | 0.003 | **0.081** | 0.330 | 0.330 |
| K03657 | pcrA | DNA helicase II / ATP-dependent DNA helicase PcrA [EC:3.6.4.12] | 0.141 | 0.140 | 0.134 | 0.156 | 0.004 | 0.450 | 0.100 | **0.077** |
| K03205 | virD4 | type IV secretion system protein VirD4 | 0.135 | 0.159 | 0.170 | 0.100 | 0.013 | 0.640 | 0.360 | **0.076** |
| K11717 | sufS | cysteine desulfurase / selenocysteine lyase [EC:2.8.1.7 4.4.1.16] | 0.133 | 0.129 | 0.134 | 0.143 | 0.003 | **0.087** | 0.590 | 0.130 |
| K01714 | dapA | 4-hydroxy-tetrahydrodipicolinate synthase [EC:4.3.3.7] | 0.132 | 0.141 | 0.144 | 0.122 | 0.004 | 0.640 | 0.440 | **0.078** |
| **K00656** | **pflD** | **formate C-acetyltransferase [EC:2.3.1.54]** | 0.129 | 0.138 | 0.146 | 0.118 | 0.005 | 0.870 | 0.320 | **0.068** |
| K03086 | rpoD | RNA polymerase primary sigma factor | 0.129 | 0.119 | 0.126 | 0.144 | 0.005 | 0.170 | 0.600 | **0.087** |
| K00764 | purF | amidophosphoribosyltransferase [EC:2.4.2.14] | 0.127 | 0.131 | 0.133 | 0.117 | 0.003 | 0.290 | 0.130 | **0.023** |
| K01784 | galE | UDP-glucose 4-epimerase [EC:5.1.3.2] | 0.125 | 0.120 | 0.120 | 0.131 | 0.002 | 0.500 | 0.530 | **0.087** |
| K01179 | E3.2.1.4 | endoglucanase [EC:3.2.1.4] | 0.126 | 0.128 | 0.138 | 0.101 | 0.007 | 0.480 | **0.098** | **0.072** |
| K01209 | abfA | alpha-N-arabinofuranosidase [EC:3.2.1.55] | 0.113 | 0.121 | 0.131 | 0.106 | 0.005 | 0.860 | 0.340 | **0.075** |
| K00945 | cmk | CMP/dCMP kinase [EC:2.7.4.25] | 0.118 | 0.113 | 0.113 | 0.124 | 0.002 | 0.520 | 0.490 | **0.065** |
| K03798 | ftsH | cell division protease FtsH [EC:3.4.24.-] | 0.114 | 0.115 | 0.110 | 0.123 | 0.002 | 0.370 | **0.014** | **0.017** |
| K03427 | hsdM | type I restriction enzyme M protein [EC:2.1.1.72] | 0.123 | 0.117 | 0.116 | 0.103 | 0.004 | **0.041** | **0.045** | 0.450 |
| K09458 | fabF | 3-oxoacyl-[acyl-carrier-protein] synthase II [EC:2.3.1.179] | 0.115 | 0.113 | 0.119 | 0.106 | 0.002 | 0.740 | **0.077** | 0.150 |
| K01448 | amiABC | N-acetylmuramoyl-L-alanine amidase [EC:3.5.1.28] | 0.115 | 0.112 | 0.117 | 0.108 | 0.002 | 0.740 | **0.075** | 0.330 |
| K01462 | PDF | peptide deformylase [EC:3.5.1.88] | 0.108 | 0.110 | 0.108 | 0.119 | 0.002 | 0.100 | **0.016** | **0.072** |
| K00789 | metK | S-adenosylmethionine synthetase [EC:2.5.1.6] | 0.110 | 0.114 | 0.116 | 0.103 | 0.003 | 0.580 | 0.310 | **0.054** |
| K01912 | paaK | phenylacetate-CoA ligase [EC:6.2.1.30] | 0.112 | 0.113 | 0.120 | 0.096 | 0.004 | 0.470 | **0.069** | **0.051** |
| K00527 | rtpR | ribonucleoside-triphosphate reductase (thioredoxin) [EC:1.17.4.2] | 0.107 | 0.112 | 0.115 | 0.105 | 0.002 | 0.930 | 0.530 | **0.094** |
| K03768 | ppiB | peptidyl-prolyl cis-trans isomerase B (cyclophilin B) [EC:5.2.1.8] | 0.108 | 0.112 | 0.114 | 0.103 | 0.002 | 0.800 | 0.350 | **0.056** |
| K00991 | ispD | 2-C-methyl-D-erythritol 4-phosphate cytidylyltransferase [EC:2.7.7.60] | 0.106 | 0.103 | 0.107 | 0.119 | 0.003 | **0.098** | 0.380 | 0.130 |
| K00548 | metH | 5-methyltetrahydrofolate--homocysteine methyltransferase [EC:2.1.1.13] | 0.108 | 0.110 | 0.113 | 0.100 | 0.002 | 0.490 | 0.140 | **0.042** |
| K01649 | leuA | 2-isopropylmalate synthase [EC:2.3.3.13] | 0.105 | 0.107 | 0.110 | 0.094 | 0.003 | 0.360 | 0.120 | **0.053** |
| K00820 | glmS | glucosamine-fructose-6-phosphate aminotransferase (isomerizing) [EC:2.6.1.16] | 0.102 | 0.107 | 0.109 | 0.096 | 0.003 | 0.560 | 0.290 | **0.046** |
| K01619 | deoC | deoxyribose-phosphate aldolase [EC:4.1.2.4] | 0.098 | 0.105 | 0.111 | 0.098 | 0.003 | 0.600 | 0.530 | **0.078** |
| K02051 | ABC.SN.S | NitT/TauT family transport system substrate-binding protein | 0.100 | 0.097 | 0.102 | 0.110 | 0.002 | **0.046** | 0.480 | 0.140 |
| K00655 | plsC | 1-acyl-sn-glycerol-3-phosphate acyltransferase [EC:2.3.1.51] | 0.102 | 0.099 | 0.097 | 0.104 | 0.001 | 0.990 | 0.410 | **0.029** |
| K00215 | dapB | 4-hydroxy-tetrahydrodipicolinate reductase [EC:1.17.1.8] | 0.101 | 0.104 | 0.106 | 0.092 | 0.003 | 0.400 | 0.200 | **0.075** |
| K03545 | tig | trigger factor | 0.098 | 0.097 | 0.095 | 0.101 | 0.001 | 0.360 | **0.028** | **0.005** |
| K05515 | mrdA | penicillin-binding protein 2 [EC:3.4.16.4] | 0.097 | 0.100 | 0.105 | 0.089 | 0.003 | 0.720 | 0.120 | **0.035** |
| K04043 | dnaK | molecular chaperone DnaK | 0.097 | 0.097 | 0.095 | 0.099 | 0.001 | 0.740 | **0.022** | **0.027** |
| K01952 | purL | phosphoribosylformylglycinamidine synthase [EC:6.3.5.3] | 0.098 | 0.098 | 0.095 | 0.093 | 0.001 | **0.047** | 0.780 | 0.600 |
| K03624 | greA | transcription elongation factor GreA | 0.096 | 0.095 | 0.094 | 0.100 | 0.001 | 0.330 | **0.013** | **0.010** |
| K00931 | proB | glutamate 5-kinase [EC:2.7.2.11] | 0.093 | 0.097 | 0.104 | 0.088 | 0.003 | 0.780 | 0.240 | **0.046** |
| K03438 | mraW | 16S rRNA (cytosine1402-N4)-methyltransferase [EC:2.1.1.199] | 0.095 | 0.094 | 0.093 | 0.100 | 0.001 | **0.083** | **0.005** | **0.002** |
| K01338 | lon | ATP-dependent Lon protease [EC:3.4.21.53] | 0.099 | 0.095 | 0.094 | 0.092 | 0.001 | **0.088** | 0.180 | 0.520 |
| K03101 | lspA | signal peptidase II [EC:3.4.23.36] | 0.096 | 0.092 | 0.094 | 0.095 | 0.001 | 0.450 | 0.290 | **0.023** |
| K01874 | metG | methionyl-tRNA synthetase [EC:6.1.1.10] | 0.092 | 0.093 | 0.094 | 0.098 | 0.001 | **0.039** | **0.096** | 0.310 |
| K03588 | ftsW | cell division protein FtsW | 0.093 | 0.093 | 0.092 | 0.098 | 0.001 | 0.190 | **0.032** | **0.027** |
| K02314 | dnaB | replicative DNA helicase [EC:3.6.4.12] | 0.093 | 0.092 | 0.093 | 0.097 | 0.001 | **0.013** | **0.030** | **0.012** |
| K01153 | hsdR | type I restriction enzyme R subunit [EC:3.1.21.3] | 0.098 | 0.093 | 0.094 | 0.088 | 0.002 | **0.052** | **0.020** | 0.590 |
| K06173 | truA | tRNA pseudouridine38-40 synthase [EC:5.4.99.12] | 0.092 | 0.092 | 0.092 | 0.096 | 0.001 | 0.190 | **0.096** | **0.060** |
| K01868 | thrS | threonyl-tRNA synthetase [EC:6.1.1.3] | 0.092 | 0.093 | 0.092 | 0.094 | 0.000 | 0.200 | **0.002** | **0.039** |
| K01662 | dxs | 1-deoxy-D-xylulose-5-phosphate synthase [EC:2.2.1.7] | 0.094 | 0.090 | 0.092 | 0.092 | 0.001 | 0.860 | **0.063** | **0.077** |
| K08591 | plsY | glycerol-3-phosphate acyltransferase PlsY [EC:2.3.1.15] | 0.090 | 0.099 | 0.099 | 0.080 | 0.004 | 0.540 | 0.520 | **0.082** |
| K01881 | proS | prolyl-tRNA synthetase [EC:6.1.1.15] | 0.091 | 0.091 | 0.091 | 0.092 | 0.000 | **0.056** | 0.210 | 0.110 |
| K01972 | ligA | DNA ligase (NAD+) [EC:6.5.1.2] | 0.090 | 0.091 | 0.091 | 0.093 | 0.001 | **0.053** | **0.089** | 0.290 |
| K02356 | efp | elongation factor P | 0.090 | 0.090 | 0.090 | 0.093 | 0.001 | **0.051** | **0.060** | 0.150 |
| K00604 | fmt | methionyl-tRNA formyltransferase [EC:2.1.2.9] | 0.090 | 0.090 | 0.090 | 0.092 | 0.001 | 0.120 | **0.009** | 0.120 |
| K02337 | dnaE | DNA polymerase III subunit alpha [EC:2.7.7.7] | 0.090 | 0.088 | 0.089 | 0.092 | 0.001 | 0.130 | 0.690 | **0.034** |
| K06881 | nrnA | bifunctional oligoribonuclease and PAP phosphatase NrnA [EC:3.1.3.7 3.1.13.3] | 0.089 | 0.088 | 0.090 | 0.093 | 0.001 | **0.094** | 0.740 | 0.190 |
| K07478 | ycaJ | putative ATPase | 0.089 | 0.088 | 0.090 | 0.092 | 0.001 | **0.005** | 0.200 | **0.023** |
| K00700 | glgB | 4-alpha-glucan branching enzyme [EC:2.4.1.18] | 0.089 | 0.088 | 0.088 | 0.094 | 0.001 | 0.140 | 0.100 | **0.098** |
| K00927 | PGK | phosphoglycerate kinase [EC:2.7.2.3] | 0.089 | 0.090 | 0.090 | 0.091 | 0.000 | **0.094** | 0.260 | 0.700 |
| K02343 | dnaX | DNA polymerase III subunit gamma/tau [EC:2.7.7.7] | 0.089 | 0.088 | 0.089 | 0.092 | 0.001 | **0.054** | 0.570 | **0.083** |
| K00981 | E2.7.7.41 | phosphatidate cytidylyltransferase [EC:2.7.7.41] | 0.089 | 0.089 | 0.089 | 0.091 | 0.000 | **0.099** | 0.380 | 0.250 |
| K07027 | K07027 | glycosyltransferase 2 family protein | 0.089 | 0.091 | 0.099 | 0.079 | 0.004 | 0.830 | **0.082** | **0.039** |
| K03070 | secA | preprotein translocase subunit SecA | 0.089 | 0.089 | 0.089 | 0.091 | 0.000 | **0.093** | 0.370 | 0.240 |
| K01937 | pyrG | CTP synthase [EC:6.3.4.2] | 0.089 | 0.089 | 0.090 | 0.090 | 0.000 | **0.090** | 0.880 | 1.000 |
| K03106 | ffh | signal recognition particle subunit SRP54 [EC:3.6.5.4] | 0.089 | 0.088 | 0.090 | 0.090 | 0.000 | **0.021** | 0.770 | 0.510 |
| K03110 | ftsY | fused signal recognition particle receptor | 0.089 | 0.088 | 0.090 | 0.090 | 0.000 | **0.021** | 0.770 | 0.510 |
| K06187 | recR | recombination protein RecR | 0.089 | 0.089 | 0.089 | 0.090 | 0.000 | **0.059** | 0.460 | 0.170 |
| K02909 | rpmE | large subunit ribosomal protein L31 | 0.089 | 0.088 | 0.089 | 0.090 | 0.000 | **0.051** | 0.640 | 0.210 |
| K01810 | pgi | glucose-6-phosphate isomerase [EC:5.3.1.9] | 0.089 | 0.088 | 0.089 | 0.090 | 0.000 | **0.055** | 0.470 | 0.210 |
| K01056 | PTH1 | peptidyl-tRNA hydrolase-PTH1 family [EC:3.1.1.29] | 0.089 | 0.088 | 0.089 | 0.090 | 0.000 | **0.057** | 0.670 | 0.190 |
| K02884 | MRPL19 | large subunit ribosomal protein L19 | 0.089 | 0.088 | 0.089 | 0.090 | 0.000 | **0.061** | 0.720 | 0.210 |
| K02926 | MRPL4 | large subunit ribosomal protein L4 | 0.089 | 0.088 | 0.089 | 0.090 | 0.000 | **0.061** | 0.720 | 0.210 |
| K03977 | engA | GTPase | 0.089 | 0.088 | 0.089 | 0.090 | 0.000 | **0.057** | 0.710 | 0.200 |
| K02357 | tsf | elongation factor Ts | 0.089 | 0.088 | 0.089 | 0.090 | 0.000 | **0.061** | 0.730 | 0.210 |
| K02835 | prfA | peptide chain release factor 1 | 0.089 | 0.088 | 0.089 | 0.090 | 0.000 | **0.061** | 0.730 | 0.210 |
| K02935 | MRPL12 | large subunit ribosomal protein L7/L12 | 0.089 | 0.088 | 0.089 | 0.090 | 0.000 | **0.061** | 0.730 | 0.210 |
| K02959 | MRPS16 | small subunit ribosomal protein S16 | 0.089 | 0.088 | 0.089 | 0.090 | 0.000 | **0.061** | 0.730 | 0.210 |
| K02838 | frr | ribosome recycling factor | 0.089 | 0.088 | 0.089 | 0.090 | 0.000 | **0.058** | 0.710 | 0.220 |
| K02879 | MRPL17 | large subunit ribosomal protein L17 | 0.089 | 0.088 | 0.089 | 0.090 | 0.000 | **0.060** | 0.720 | 0.220 |
| K02990 | MRPS6 | small subunit ribosomal protein S6 | 0.089 | 0.088 | 0.089 | 0.090 | 0.000 | **0.060** | 0.720 | 0.220 |
| K03664 | smpB | SsrA-binding protein | 0.089 | 0.088 | 0.089 | 0.090 | 0.000 | **0.056** | 0.700 | 0.230 |
| K02338 | dnaN | DNA polymerase III subunit beta [EC:2.7.7.7] | 0.089 | 0.088 | 0.089 | 0.090 | 0.000 | **0.064** | 0.810 | 0.250 |
| K02878 | MRPL16 | large subunit ribosomal protein L16 | 0.089 | 0.088 | 0.089 | 0.090 | 0.000 | **0.057** | 0.690 | 0.240 |
| K02834 | rbfA | ribosome-binding factor A | 0.089 | 0.088 | 0.089 | 0.090 | 0.000 | **0.056** | 0.730 | 0.250 |
| K03625 | nusB | N utilization substance protein B | 0.089 | 0.088 | 0.089 | 0.090 | 0.000 | **0.059** | 0.790 | 0.270 |
| K03551 | ruvB | holliday junction DNA helicase RuvB [EC:3.6.4.12] | 0.089 | 0.088 | 0.089 | 0.090 | 0.000 | **0.077** | 0.860 | 0.350 |
| K03553 | recA | recombination protein RecA | 0.088 | 0.088 | 0.089 | 0.090 | 0.000 | **0.055** | **0.096** | 0.230 |
| K02335 | polA | DNA polymerase I [EC:2.7.7.7] | 0.088 | 0.088 | 0.089 | 0.090 | 0.001 | **0.039** | 0.380 | 0.610 |
| K03695 | clpB | ATP-dependent Clp protease ATP-binding subunit ClpB | 0.088 | 0.088 | 0.089 | 0.090 | 0.000 | **0.041** | 0.550 | 0.150 |
| **K00826** | **ilvE** | **branched-chain amino acid aminotransferase [EC:2.6.1.42]** | 0.090 | 0.089 | 0.087 | 0.088 | 0.001 | **0.087** | 0.820 | 0.380 |
| **K00925** | **ackA** | **acetate kinase [EC:2.7.2.1]** | 0.088 | 0.087 | 0.088 | 0.091 | 0.001 | **0.034** | 0.330 | **0.092** |
| K02316 | dnaG | DNA primase [EC:2.7.7.-] | 0.088 | 0.088 | 0.089 | 0.090 | 0.000 | **0.044** | 0.440 | 0.650 |
| K00942 | E2.7.4.8 | guanylate kinase [EC:2.7.4.8] | 0.087 | 0.088 | 0.089 | 0.090 | 0.000 | **0.042** | 0.170 | 0.550 |
| **K01689** | **ENO** | **enolase [EC:4.2.1.11]** | 0.087 | 0.086 | 0.088 | 0.091 | 0.001 | **0.070** | 0.700 | 0.200 |
| K03574 | mutT | 8-oxo-dGTP diphosphatase [EC:3.6.1.55] | 0.086 | 0.086 | 0.084 | 0.096 | 0.002 | 0.140 | **0.038** | **0.037** |
| K00962 | pnp | polyribonucleotide nucleotidyltransferase [EC:2.7.7.8] | 0.088 | 0.087 | 0.088 | 0.089 | 0.000 | 0.120 | 0.980 | **0.096** |
| K03501 | rsmG | 16S rRNA (guanine527-N7)-methyltransferase [EC:2.1.1.170] | 0.087 | 0.087 | 0.088 | 0.089 | 0.000 | **0.089** | 0.320 | 0.530 |
| K00773 | tgt | queuine tRNA-ribosyltransferase [EC:2.4.2.29] | 0.089 | 0.086 | 0.089 | 0.087 | 0.001 | 0.530 | **0.063** | 0.690 |
| K01533 | copB | Cu2+-exporting ATPase [EC:3.6.3.4] | 0.087 | 0.087 | 0.089 | 0.089 | 0.001 | **0.030** | 0.910 | 0.830 |
| K01733 | thrC | threonine synthase [EC:4.2.3.1] | 0.089 | 0.089 | 0.088 | 0.086 | 0.001 | **0.095** | 0.280 | 0.570 |
| K03979 | obgE | GTPase [EC:3.6.5.-] | 0.087 | 0.087 | 0.088 | 0.089 | 0.000 | **0.084** | 0.260 | 0.590 |
| K10947 | padR | PadR family transcriptional regulator; regulatory protein PadR | 0.084 | 0.094 | 0.096 | 0.077 | 0.004 | 0.680 | 0.520 | **0.048** |
| K00278 | nadB | L-aspartate oxidase [EC:1.4.3.16] | 0.085 | 0.089 | 0.096 | 0.080 | 0.003 | 0.820 | 0.220 | **0.042** |
| K02563 | murG | UDP-N-acetylglucosamine--N-acetylmuramyl-(pentapeptide) pyrophosphoryl-undecaprenol N-acetylglucosamine transferase [EC:2.4.1.227] | 0.089 | 0.088 | 0.087 | 0.086 | 0.000 | **0.079** | 0.490 | 0.930 |
| K02518 | infA | translation initiation factor IF-1 | 0.087 | 0.085 | 0.088 | 0.089 | 0.001 | **0.065** | 0.990 | 0.220 |
| K02899 | MRPL27 | large subunit ribosomal protein L27 | 0.087 | 0.085 | 0.088 | 0.089 | 0.001 | **0.065** | 0.960 | 0.220 |
| K01775 | alr | alanine racemase [EC:5.1.1.1] | 0.088 | 0.087 | 0.088 | 0.085 | 0.000 | 0.260 | **0.075** | 0.450 |
| **K00873** | **PK** | **pyruvate kinase [EC:2.7.1.40]** | 0.084 | 0.087 | 0.086 | 0.089 | 0.001 | 0.220 | **0.015** | 0.840 |
| K02519 | infB | translation initiation factor IF-2 | 0.086 | 0.085 | 0.087 | 0.089 | 0.001 | **0.053** | 0.940 | 0.440 |
| K03284 | corA | magnesium transporter | 0.086 | 0.085 | 0.087 | 0.088 | 0.000 | **0.077** | 0.790 | 0.450 |
| K02968 | rpsT | small subunit ribosomal protein S20 | 0.086 | 0.085 | 0.087 | 0.088 | 0.001 | **0.054** | 0.990 | 0.520 |
| K00008 | gutB | L-iditol 2-dehydrogenase [EC:1.1.1.14] | 0.082 | 0.097 | 0.107 | 0.060 | 0.009 | 0.680 | 0.330 | **0.072** |
| K03439 | trmB | tRNA (guanine-N7-)-methyltransferase [EC:2.1.1.33] | 0.086 | 0.085 | 0.087 | 0.088 | 0.001 | **0.014** | 0.910 | 0.380 |
| K02911 | MRPL32 | large subunit ribosomal protein L32 | 0.086 | 0.085 | 0.087 | 0.088 | 0.001 | **0.079** | 0.970 | 0.460 |
| K02914 | MRPL34 | large subunit ribosomal protein L34 | 0.085 | 0.085 | 0.086 | 0.087 | 0.000 | **0.080** | 0.390 | 0.510 |
| K03324 | yjbB | phosphate:Na+ symporter | 0.087 | 0.083 | 0.085 | 0.088 | 0.001 | 0.340 | 0.980 | **0.086** |
| K00075 | murB | UDP-N-acetylmuramate dehydrogenase [EC:1.3.1.98] | 0.084 | 0.084 | 0.087 | 0.088 | 0.001 | **0.085** | 0.700 | 0.690 |
| K00919 | ispE | 4-diphosphocytidyl-2-C-methyl-D-erythritol kinase [EC:2.7.1.148] | 0.085 | 0.085 | 0.087 | 0.086 | 0.000 | **0.014** | 0.530 | 0.140 |
| K03075 | secG | preprotein translocase subunit SecG | 0.085 | 0.084 | 0.087 | 0.087 | 0.001 | **0.093** | 0.830 | 0.860 |
| K01537 | E3.6.3.8 | Ca2+-transporting ATPase [EC:3.6.3.8] | 0.081 | 0.085 | 0.088 | 0.087 | 0.001 | **0.035** | 0.440 | 0.140 |
| K03595 | era | GTPase | 0.083 | 0.084 | 0.086 | 0.087 | 0.001 | **0.084** | 0.370 | 0.770 |
| **K01572** | **oadB** | **oxaloacetate decarboxylase beta subunit [EC:4.1.1.112]** | 0.086 | 0.087 | 0.095 | 0.072 | 0.004 | 0.650 | **0.091** | **0.060** |
| K03073 | secE | preprotein translocase subunit SecE | 0.086 | 0.085 | 0.086 | 0.083 | 0.001 | 0.420 | **0.045** | 0.250 |
| K06153 | bacA | undecaprenyl-diphosphatase [EC:3.6.1.27] | 0.083 | 0.083 | 0.085 | 0.086 | 0.001 | **0.018** | 0.410 | 0.600 |
| K03584 | recO | DNA repair protein RecO (recombination protein O) | 0.084 | 0.082 | 0.085 | 0.085 | 0.001 | **0.083** | 0.750 | 0.260 |
| K00133 | asd | aspartate-semialdehyde dehydrogenase [EC:1.2.1.11] | 0.085 | 0.085 | 0.084 | 0.082 | 0.001 | **0.080** | 0.330 | 0.610 |
| K06949 | rsgA | ribosome biogenesis GTPase / thiamine phosphate phosphatase [EC:3.6.1.- 3.1.3.100] | 0.082 | 0.081 | 0.085 | 0.087 | 0.001 | **0.031** | 0.870 | 0.460 |
| K00099 | dxr | 1-deoxy-D-xylulose-5-phosphate reductoisomerase [EC:1.1.1.267] | 0.083 | 0.081 | 0.085 | 0.084 | 0.001 | **0.051** | 0.460 | 0.570 |
| K00761 | upp | uracil phosphoribosyltransferase [EC:2.4.2.9] | 0.081 | 0.082 | 0.084 | 0.086 | 0.001 | **0.088** | 0.470 | 0.820 |
| K00760 | hpt | hypoxanthine phosphoribosyltransferase [EC:2.4.2.8] | 0.082 | 0.081 | 0.084 | 0.085 | 0.001 | **0.048** | 0.940 | 0.480 |
| K00286 | proC | pyrroline-5-carboxylate reductase [EC:1.5.1.2] | 0.082 | 0.082 | 0.085 | 0.083 | 0.000 | **0.099** | 0.600 | 0.320 |
| K12573 | rnr | ribonuclease R [EC:3.1.-.-] | 0.081 | 0.081 | 0.085 | 0.085 | 0.001 | **0.041** | 0.940 | 0.910 |
| K03152 | thiJ | protein deglycase [EC:3.5.1.124] | 0.081 | 0.081 | 0.085 | 0.086 | 0.001 | **0.010** | 0.670 | 0.850 |
| K03282 | mscL | large conductance mechanosensitive channel | 0.081 | 0.081 | 0.083 | 0.084 | 0.001 | **0.056** | 0.890 | 0.570 |
| K01243 | mtn | adenosylhomocysteine nucleosidase [EC:3.2.2.9] | 0.081 | 0.079 | 0.084 | 0.085 | 0.001 | **0.011** | 0.780 | 0.450 |
| K05837 | rodA | rod shape determining protein RodA | 0.083 | 0.082 | 0.084 | 0.080 | 0.001 | 0.740 | **0.093** | 0.370 |
| K07391 | comM | magnesium chelatase family protein | 0.082 | 0.081 | 0.084 | 0.081 | 0.001 | 0.440 | **0.059** | 0.360 |
| K00783 | rlmH | 23S rRNA (pseudouridine1915-N3)-methyltransferase [EC:2.1.1.177] | 0.081 | 0.080 | 0.083 | 0.083 | 0.001 | **0.011** | 0.440 | 0.510 |
| K01776 | murI | glutamate racemase [EC:5.1.1.3] | 0.083 | 0.080 | 0.083 | 0.080 | 0.001 | 0.810 | **0.072** | 0.850 |
| K06207 | bipA | GTP-binding protein | 0.081 | 0.080 | 0.083 | 0.083 | 0.001 | **0.099** | 0.700 | 0.780 |
| K07082 | UPF0755 | UPF0755 protein | 0.080 | 0.081 | 0.083 | 0.082 | 0.001 | **0.092** | 0.960 | 0.720 |
| **K00018** | **hprA** | **glycerate dehydrogenase [EC:1.1.1.29]** | 0.078 | 0.083 | 0.090 | 0.074 | 0.003 | 0.700 | 0.300 | **0.049** |
| **K11175** | **purN** | **phosphoribosylglycinamide formyltransferase 1 [EC:2.1.2.2]** | 0.082 | 0.083 | 0.084 | 0.075 | 0.002 | **0.080** | **0.046** | **0.018** |
| **K00831** | **serC** | **phosphoserine aminotransferase [EC:2.6.1.52]** | 0.079 | 0.080 | 0.082 | 0.082 | 0.001 | **0.009** | 0.930 | 0.450 |
| K04485 | radA | DNA repair protein RadA/Sms | 0.083 | 0.080 | 0.081 | 0.077 | 0.001 | **0.090** | **0.034** | 0.690 |
| K01893 | asnS | asparaginyl-tRNA synthetase [EC:6.1.1.22] | 0.077 | 0.078 | 0.083 | 0.083 | 0.001 | **0.010** | 0.810 | 0.880 |
| K03210 | yajC | preprotein translocase subunit YajC | 0.083 | 0.080 | 0.080 | 0.077 | 0.001 | **0.064** | **0.032** | 0.780 |
| K01770 | ispF | 2-C-methyl-D-erythritol 2,4-cyclodiphosphate synthase [EC:4.6.1.12] | 0.078 | 0.077 | 0.081 | 0.081 | 0.001 | **0.020** | 0.560 | 0.690 |
| K00567 | ogt | methylated-DNA-[protein]-cysteine S-methyltransferase [EC:2.1.1.63] | 0.082 | 0.082 | 0.083 | 0.071 | 0.003 | **0.063** | **0.034** | **0.032** |
| K00147 | proA | glutamate-5-semialdehyde dehydrogenase [EC:1.2.1.41] | 0.077 | 0.078 | 0.081 | 0.080 | 0.001 | **0.067** | 0.930 | 0.260 |
| K06178 | rluB | 23S rRNA pseudouridine2605 synthase [EC:5.4.99.22] | 0.079 | 0.076 | 0.081 | 0.075 | 0.001 | 0.830 | **0.067** | 0.500 |
| K14441 | rimO | ribosomal protein S12 methylthiotransferase [EC:2.8.4.4] | 0.079 | 0.077 | 0.078 | 0.075 | 0.001 | 0.280 | **0.050** | 0.600 |
| K02687 | prmA | ribosomal protein L11 methyltransferase [EC:2.1.1.-] | 0.078 | 0.076 | 0.079 | 0.075 | 0.001 | 0.770 | **0.020** | 0.380 |
| **K02034** | **ABC.PE.P** | **peptide/nickel transport system permease protein** | 0.078 | 0.075 | 0.067 | 0.087 | 0.004 | 0.920 | 0.190 | **0.096** |
| K01953 | asnB | asparagine synthase (glutamine-hydrolysing) [EC:6.3.5.4] | 0.072 | 0.080 | 0.072 | 0.082 | 0.002 | 0.810 | **0.033** | 0.750 |
| K01159 | ruvC | crossover junction endodeoxyribonuclease RuvC [EC:3.1.22.4] | 0.076 | 0.075 | 0.079 | 0.071 | 0.001 | 0.770 | **0.025** | **0.055** |
| K01696 | trpB | tryptophan synthase beta chain [EC:4.2.1.20] | 0.078 | 0.075 | 0.078 | 0.068 | 0.002 | 0.300 | **0.082** | 0.260 |
| K02913 | MRPL33 | large subunit ribosomal protein L33 | 0.072 | 0.069 | 0.069 | 0.085 | 0.003 | 0.200 | 0.160 | **0.049** |
| **K02112** | **atpD** | **F-type H+-transporting ATPase subunit beta [EC:7.1.2.2]** | 0.073 | 0.071 | 0.066 | 0.085 | 0.003 | 0.520 | **0.082** | **0.050** |
| **K02114** | **atpC** | **F-type H+-transporting ATPase subunit epsilon** | 0.073 | 0.071 | 0.065 | 0.085 | 0.003 | 0.520 | **0.081** | **0.049** |
| **K02115** | **atpG** | **F-type H+-transporting ATPase subunit gamma** | 0.073 | 0.071 | 0.066 | 0.085 | 0.003 | 0.510 | **0.087** | **0.049** |
| K03786 | aroQ | 3-dehydroquinate dehydratase II [EC:4.2.1.10] | 0.073 | 0.074 | 0.076 | 0.071 | 0.001 | 0.920 | 0.410 | **0.055** |
| **K02109** | **atpF** | **F-type H+-transporting ATPase subunit b** | 0.071 | 0.072 | 0.066 | 0.084 | 0.003 | 0.540 | **0.084** | **0.095** |
| K01695 | trpA | tryptophan synthase alpha chain [EC:4.2.1.20] | 0.076 | 0.074 | 0.076 | 0.066 | 0.002 | 0.170 | **0.059** | 0.250 |
| **K01752** | **sdaA** | **L-serine dehydratase [EC:4.3.1.17]** | 0.077 | 0.069 | 0.066 | 0.080 | 0.003 | 0.980 | 0.550 | **0.044** |
| K01886 | glnS | glutaminyl-tRNA synthetase [EC:6.1.1.18] | 0.074 | 0.071 | 0.075 | 0.070 | 0.001 | 0.880 | **0.014** | 0.440 |
| **K02108** | **atpB** | **F-type H+-transporting ATPase subunit a** | 0.071 | 0.070 | 0.064 | 0.082 | 0.003 | 0.580 | **0.099** | **0.064** |
| **K02110** | **atpE** | **F-type H+-transporting ATPase subunit c** | 0.070 | 0.068 | 0.063 | 0.085 | 0.004 | 0.350 | **0.053** | **0.033** |
| **K02111** | **atpA** | **F-type H+-transporting ATPase subunit alpha [EC:7.1.2.2]** | 0.070 | 0.069 | 0.064 | 0.081 | 0.003 | 0.500 | **0.096** | **0.080** |
| K13747 | nspC | carboxynorspermidine decarboxylase [EC:4.1.1.96] | 0.070 | 0.071 | 0.073 | 0.068 | 0.001 | 0.770 | 0.200 | **0.055** |
| K03470 | rnhB | ribonuclease HII [EC:3.1.26.4] | 0.071 | 0.066 | 0.064 | 0.078 | 0.003 | 0.580 | 0.290 | **0.040** |
| K03614 | rnfD | electron transport complex protein RnfD | 0.071 | 0.071 | 0.073 | 0.061 | 0.002 | 0.190 | **0.073** | **0.080** |
| **K00180** | **iorB** | **indolepyruvate ferredoxin oxidoreductase beta subunit [EC:1.2.7.8]** | 0.070 | 0.071 | 0.074 | 0.060 | 0.003 | 0.170 | **0.025** | **0.010** |
| K00287 | folA | dihydrofolate reductase [EC:1.5.1.3] | 0.067 | 0.065 | 0.063 | 0.077 | 0.003 | 0.360 | 0.200 | **0.079** |
| K00796 | folP | dihydropteroate synthase [EC:2.5.1.15] | 0.070 | 0.068 | 0.070 | 0.064 | 0.001 | 0.460 | **0.095** | 0.340 |
| K03517 | nadA | quinolinate synthase [EC:2.5.1.72] | 0.069 | 0.069 | 0.072 | 0.061 | 0.002 | 0.480 | **0.095** | 0.110 |
| K00759 | apt | adenine phosphoribosyltransferase [EC:2.4.2.7] | 0.064 | 0.064 | 0.068 | 0.073 | 0.002 | **0.045** | 0.370 | 0.400 |
| K13993 | HSP20 | HSP20 family protein | 0.068 | 0.067 | 0.072 | 0.061 | 0.002 | 0.690 | **0.039** | **0.081** |
| K00766 | trpD | anthranilate phosphoribosyltransferase [EC:2.4.2.18] | 0.067 | 0.069 | 0.069 | 0.061 | 0.002 | 0.360 | 0.240 | **0.077** |
| K01129 | dgt | dGTPase [EC:3.1.5.1] | 0.063 | 0.062 | 0.066 | 0.072 | 0.002 | **0.056** | 0.450 | 0.350 |
| K09815 | znuA | zinc transport system substrate-binding protein | 0.068 | 0.069 | 0.071 | 0.056 | 0.003 | 0.230 | **0.061** | **0.037** |
| K00046 | idnO | gluconate 5-dehydrogenase [EC:1.1.1.69] | 0.066 | 0.067 | 0.069 | 0.058 | 0.002 | 0.370 | 0.100 | **0.071** |
| K00763 | pncB | nicotinate phosphoribosyltransferase [EC:6.3.4.21] | 0.060 | 0.063 | 0.065 | 0.071 | 0.002 | **0.070** | 0.210 | 0.520 |
| K14155 | patB | cystathione beta-lyase [EC:4.4.1.8] | 0.065 | 0.061 | 0.060 | 0.072 | 0.002 | 0.430 | 0.380 | **0.055** |
| K09817 | znuC | zinc transport system ATP-binding protein [EC:3.6.3.-] | 0.067 | 0.067 | 0.069 | 0.055 | 0.003 | 0.150 | **0.048** | **0.055** |
| K09816 | znuB | zinc transport system permease protein | 0.067 | 0.067 | 0.069 | 0.055 | 0.003 | 0.150 | **0.047** | **0.056** |
| **K02113** | **atpH** | **F-type H+-transporting ATPase subunit delta** | 0.062 | 0.061 | 0.058 | 0.076 | 0.003 | 0.250 | **0.083** | **0.053** |
| K01151 | nfo | deoxyribonuclease IV [EC:3.1.21.2] | 0.064 | 0.067 | 0.067 | 0.055 | 0.003 | 0.290 | 0.210 | **0.093** |
| K03522 | etfA | electron transfer flavoprotein alpha subunit | 0.067 | 0.059 | 0.056 | 0.067 | 0.002 | 0.830 | 0.780 | **0.082** |
| **K00177** | **korC** | **2-oxoglutarate ferredoxin oxidoreductase subunit gamma [EC:1.2.7.3]** | 0.062 | 0.064 | 0.066 | 0.057 | 0.002 | 0.660 | 0.150 | **0.045** |
| K06001 | trpB | tryptophan synthase beta chain [EC:4.2.1.20] | 0.061 | 0.062 | 0.066 | 0.059 | 0.001 | 0.700 | 0.110 | **0.058** |
| **K00176** | **korD** | **2-oxoglutarate ferredoxin oxidoreductase subunit delta [EC:1.2.7.3]** | 0.061 | 0.063 | 0.066 | 0.057 | 0.002 | 0.680 | 0.150 | **0.040** |
| **K02118** | **ntpB** | **V/A-type H+/Na+-transporting ATPase subunit B** | 0.065 | 0.064 | 0.066 | 0.052 | 0.003 | 0.130 | **0.075** | 0.110 |
| K03150 | thiH | 2-iminoacetate synthase [EC:4.1.99.19] | 0.067 | 0.064 | 0.065 | 0.051 | 0.003 | **0.052** | **0.038** | 0.170 |
| K03521 | etfB | electron transfer flavoprotein beta subunit | 0.065 | 0.058 | 0.056 | 0.067 | 0.002 | 0.910 | 0.740 | **0.090** |
| **K02124** | **ntpK** | **V/A-type H+/Na+-transporting ATPase subunit K** | 0.064 | 0.064 | 0.064 | 0.051 | 0.003 | 0.120 | **0.093** | 0.100 |
| **K02120** | **ntpD** | **V/A-type H+/Na+-transporting ATPase subunit D** | 0.064 | 0.064 | 0.064 | 0.051 | 0.003 | 0.130 | **0.093** | 0.100 |
| K03648 | UNG | uracil-DNA glycosylase [EC:3.2.2.27] | 0.060 | 0.058 | 0.054 | 0.070 | 0.003 | 0.500 | 0.160 | **0.055** |
| **K02117** | **ntpA** | **V/A-type H+/Na+-transporting ATPase subunit A [EC:7.1.2.2 7.2.2.1]** | 0.065 | 0.064 | 0.065 | 0.048 | 0.003 | 0.100 | **0.076** | 0.100 |
| K10118 | msmF | raffinose/stachyose/melibiose transport system permease protein | 0.057 | 0.069 | 0.074 | 0.041 | 0.006 | 0.650 | 0.400 | **0.092** |
| **K02121** | **ntpE** | **V/A-type H+/Na+-transporting ATPase subunit E** | 0.064 | 0.063 | 0.064 | 0.048 | 0.003 | **0.077** | **0.054** | **0.073** |
| K03823 | pat | phosphinothricin acetyltransferase [EC:2.3.1.183] | 0.058 | 0.063 | 0.064 | 0.052 | 0.002 | 0.460 | 0.320 | **0.035** |
| K07260 | vanY | zinc D-Ala-D-Ala carboxypeptidase [EC:3.4.17.14] | 0.055 | 0.064 | 0.068 | 0.049 | 0.004 | 0.830 | 0.520 | **0.083** |
| K01420 | fnr | CRP/FNR family transcriptional regulator | 0.058 | 0.064 | 0.068 | 0.046 | 0.004 | 0.630 | 0.290 | **0.084** |
| K03426 | NUDT12 | NAD+ diphosphatase [EC:3.6.1.22] | 0.058 | 0.055 | 0.055 | 0.066 | 0.002 | 0.300 | 0.360 | **0.081** |
| K03442 | mscS | small conductance mechanosensitive channel | 0.061 | 0.060 | 0.063 | 0.049 | 0.003 | 0.220 | **0.058** | **0.096** |
| K00979 | kdsB | 3-deoxy-manno-octulosonate cytidylyltransferase (CMP-KDO synthetase) [EC:2.7.7.38] | 0.058 | 0.059 | 0.063 | 0.052 | 0.002 | 0.700 | **0.091** | **0.059** |
| K03502 | umuC | DNA polymerase V | 0.057 | 0.053 | 0.053 | 0.068 | 0.003 | 0.230 | 0.210 | **0.035** |
| K03699 | tlyC | putative hemolysin | 0.057 | 0.061 | 0.061 | 0.052 | 0.002 | 0.460 | 0.520 | **0.077** |
| K03402 | ahrC | transcriptional regulator of arginine metabolism | 0.053 | 0.053 | 0.057 | 0.061 | 0.002 | **0.092** | 0.630 | 0.600 |
| K00784 | rnz | ribonuclease Z [EC:3.1.26.11] | 0.057 | 0.051 | 0.051 | 0.062 | 0.002 | 0.580 | 0.510 | **0.076** |
| K00537 | arsC | arsenate reductase [EC:1.20.4.1] | 0.055 | 0.059 | 0.057 | 0.046 | 0.002 | 0.160 | 0.360 | **0.053** |
| K14742 | tsaB | tRNA threonylcarbamoyladenosine biosynthesis protein TsaB | 0.058 | 0.058 | 0.058 | 0.044 | 0.003 | **0.056** | **0.056** | **0.046** |
| K02824 | uraA | uracil permease | 0.047 | 0.050 | 0.054 | 0.058 | 0.002 | **0.082** | 0.380 | 0.820 |
| K03534 | rhaM | L-rhamnose mutarotase [EC:5.1.3.32] | 0.051 | 0.053 | 0.058 | 0.044 | 0.002 | 0.810 | 0.120 | **0.044** |
| K11068 | hlyIII | hemolysin III | 0.045 | 0.047 | 0.053 | 0.060 | 0.003 | **0.022** | 0.260 | 0.470 |
| K01620 | ltaE | threonine aldolase [EC:4.1.2.48] | 0.050 | 0.047 | 0.048 | 0.059 | 0.002 | 0.240 | 0.250 | **0.082** |
| K07173 | luxS | S-ribosylhomocysteine lyase [EC:4.4.1.21] | 0.046 | 0.047 | 0.052 | 0.058 | 0.002 | **0.044** | 0.390 | 0.510 |
| K09888 | zapA | cell division protein ZapA | 0.046 | 0.048 | 0.051 | 0.053 | 0.001 | **0.084** | 0.540 | 0.950 |
| K01480 | speB | agmatinase [EC:3.5.3.11] | 0.050 | 0.053 | 0.054 | 0.039 | 0.003 | 0.180 | 0.120 | **0.019** |
| K01449 | cwlJ | N-acetylmuramoyl-L-alanine amidase [EC:3.5.1.28] | 0.045 | 0.053 | 0.059 | 0.032 | 0.005 | 0.610 | 0.250 | **0.048** |
| K06215 | pdx1 | pyridoxal 5'-phosphate synthase pdxS subunit [EC:4.3.3.6] | 0.042 | 0.044 | 0.050 | 0.048 | 0.002 | **0.044** | 0.880 | 0.600 |
| K10439 | rbsB | ribose transport system substrate-binding protein | 0.050 | 0.043 | 0.036 | 0.052 | 0.003 | 0.660 | 0.380 | **0.035** |
| K01442 | E3.5.1.24 | choloylglycine hydrolase [EC:3.5.1.24] | 0.043 | 0.049 | 0.049 | 0.037 | 0.002 | 0.420 | 0.480 | **0.038** |
| K08974 | K08974 | putative membrane protein | 0.046 | 0.047 | 0.049 | 0.036 | 0.003 | 0.270 | 0.120 | **0.060** |
| **K02231** | **cobP** | **adenosylcobinamide kinase / adenosylcobinamide-phosphate guanylyltransferase [EC:2.7.1.156 2.7.7.62]** | 0.046 | 0.038 | 0.039 | 0.050 | 0.002 | 0.500 | 0.720 | **0.025** |
| K08681 | pdxT | 5'-phosphate synthase pdxT subunit [EC:4.3.3.6] | 0.041 | 0.041 | 0.047 | 0.044 | 0.001 | **0.094** | 0.550 | 0.530 |
| K07301 | yrbG | cation:H+ antiporter | 0.042 | 0.039 | 0.038 | 0.052 | 0.003 | 0.290 | 0.220 | **0.055** |
| K07979 | GntR | GntR family transcriptional regulator | 0.039 | 0.042 | 0.034 | 0.055 | 0.004 | 0.400 | **0.019** | **0.069** |
| K10117 | msmE | raffinose/stachyose/melibiose transport system substrate-binding protein | 0.040 | 0.049 | 0.051 | 0.030 | 0.004 | 0.650 | 0.480 | **0.082** |
| K03816 | xpt | xanthine phosphoribosyltransferase [EC:2.4.2.22] | 0.042 | 0.038 | 0.037 | 0.051 | 0.003 | 0.370 | 0.240 | **0.052** |
| **K00001** | **adh** | **alcohol dehydrogenase [EC:1.1.1.1]** | 0.041 | 0.049 | 0.051 | 0.027 | 0.005 | 0.520 | 0.340 | **0.072** |
| K02334 | dpo | DNA polymerase bacteriophage-type [EC:2.7.7.7] | 0.042 | 0.046 | 0.046 | 0.032 | 0.003 | 0.220 | 0.200 | **0.037** |
| K04042 | glmU | bifunctional UDP-N-acetylglucosamine pyrophosphorylase / Glucosamine-1-phosphate N-acetyltransferase [EC:2.7.7.23 2.3.1.157] | 0.040 | 0.043 | 0.036 | 0.046 | 0.002 | 0.850 | **0.020** | 0.260 |
| K07491 | K07491 | putative transposase | 0.038 | 0.039 | 0.034 | 0.050 | 0.003 | 0.360 | **0.057** | **0.087** |
| K11105 | cvrA | cell volume regulation protein A | 0.042 | 0.044 | 0.046 | 0.028 | 0.004 | 0.110 | **0.058** | **0.020** |
| K01447 | xlyAB | N-acetylmuramoyl-L-alanine amidase [EC:3.5.1.28] | 0.038 | 0.042 | 0.045 | 0.033 | 0.002 | 0.680 | 0.200 | **0.023** |
| K00963 | UGP2 | UTP--glucose-1-phosphate uridylyltransferase [EC:2.7.7.9] | 0.035 | 0.041 | 0.042 | 0.029 | 0.003 | 0.570 | 0.500 | **0.045** |
| K01546 | kdpA | K+-transporting ATPase ATPase A chain [EC:3.6.3.12] | 0.036 | 0.040 | 0.042 | 0.025 | 0.003 | 0.310 | 0.100 | **0.021** |
| K01547 | kdpB | K+-transporting ATPase ATPase B chain [EC:3.6.3.12] | 0.036 | 0.040 | 0.042 | 0.025 | 0.003 | 0.310 | 0.100 | **0.021** |
| K03292 | TC.GPH | glycoside/pentoside/hexuronide:cation symporter | 0.033 | 0.030 | 0.030 | 0.050 | 0.004 | 0.110 | 0.110 | **0.031** |
| K08309 | slt | soluble lytic murein transglycosylase [EC:4.2.2.-] | 0.036 | 0.038 | 0.041 | 0.027 | 0.003 | 0.460 | 0.130 | **0.062** |
| K01251 | ahcY | adenosylhomocysteinase [EC:3.3.1.1] | 0.040 | 0.039 | 0.035 | 0.027 | 0.003 | **0.076** | 0.290 | 0.490 |
| K03458 | TC.NCS2 | nucleobase:cation symporter-2 | 0.034 | 0.030 | 0.030 | 0.046 | 0.003 | 0.140 | 0.190 | **0.031** |
| K08483 | ptsI | phosphotransferase system enzyme I PtsI [EC:2.7.3.9] | 0.035 | 0.032 | 0.030 | 0.043 | 0.002 | 0.380 | **0.076** | **0.018** |
| K03074 | secF | preprotein translocase subunit SecF | 0.033 | 0.038 | 0.039 | 0.026 | 0.002 | 0.470 | 0.310 | **0.051** |
| **K01997** | **livH** | **branched-chain amino acid transport system permease protein** | 0.034 | 0.034 | 0.029 | 0.038 | 0.002 | 0.910 | **0.092** | 0.140 |
| **K02072** | **metI** | **D-methionine transport system permease protein** | 0.036 | 0.030 | 0.030 | 0.039 | 0.002 | 0.610 | 0.490 | **0.016** |
| **K00865** | **glxK** | **glycerate 2-kinase [EC:2.7.1.165]** | 0.033 | 0.031 | 0.029 | 0.042 | 0.002 | 0.340 | 0.230 | **0.093** |
| K01421 | yhgE | putative membrane protein | 0.031 | 0.033 | 0.028 | 0.042 | 0.003 | 0.430 | **0.036** | 0.130 |
| **K00074** | **paaH** | **3-hydroxybutyryl-CoA dehydrogenase [EC:1.1.1.157]** | 0.037 | 0.032 | 0.029 | 0.036 | 0.002 | 0.440 | 0.560 | **0.040** |
| K01269 | E3.4.11.- | aminopeptidase [EC:3.4.11.-] | 0.032 | 0.033 | 0.028 | 0.037 | 0.002 | 0.910 | **0.086** | 0.130 |
| K06374 | spmB | spore maturation protein B | 0.031 | 0.035 | 0.037 | 0.027 | 0.002 | 0.680 | 0.460 | **0.091** |
| K06373 | spmA | spore maturation protein A | 0.031 | 0.035 | 0.036 | 0.026 | 0.002 | 0.660 | 0.430 | **0.088** |
| K06392 | spoIIIAC | stage III sporulation protein AC | 0.031 | 0.034 | 0.036 | 0.027 | 0.002 | 0.670 | 0.380 | **0.096** |
| **K01962** | **accA** | **acetyl-CoA carboxylase carboxyl transferase subunit alpha [EC:6.4.1.2 2.1.3.15]** | 0.030 | 0.037 | 0.034 | 0.026 | 0.002 | 0.380 | 0.920 | **0.076** |
| K06393 | spoIIIAD | stage III sporulation protein AD | 0.031 | 0.034 | 0.035 | 0.026 | 0.002 | 0.620 | 0.380 | **0.087** |
| K06334 | cotJC | spore coat protein JC | 0.030 | 0.034 | 0.035 | 0.026 | 0.002 | 0.750 | 0.420 | **0.077** |
| K03306 | TC.PIT | inorganic phosphate transporter | 0.028 | 0.033 | 0.038 | 0.026 | 0.002 | 0.710 | 0.380 | **0.053** |
| **K10188** | **lacE** | **lactose/L-arabinose transport system substrate-binding protein** | 0.030 | 0.033 | 0.035 | 0.025 | 0.002 | 0.660 | 0.340 | **0.076** |
| K03072 | secD | preprotein translocase subunit SecD | 0.030 | 0.034 | 0.036 | 0.023 | 0.003 | 0.530 | 0.220 | **0.057** |
| K04769 | AbrB | AbrB family transcriptional regulator stage V sporulation protein T | 0.030 | 0.033 | 0.035 | 0.024 | 0.002 | 0.570 | 0.320 | **0.065** |
| K00940 | ndk | nucleoside-diphosphate kinase [EC:2.7.4.6] | 0.035 | 0.033 | 0.031 | 0.023 | 0.002 | **0.084** | 0.250 | 0.430 |
| **K01998** | **livM** | **branched-chain amino acid transport system permease protein** | 0.030 | 0.031 | 0.025 | 0.036 | 0.002 | 0.770 | **0.036** | **0.074** |
| K04041 | fbp3 | fructose-1,6-bisphosphatase III [EC:3.1.3.11] | 0.029 | 0.026 | 0.027 | 0.041 | 0.003 | 0.130 | 0.180 | **0.054** |
| K06387 | spoIIR | stage II sporulation protein R | 0.030 | 0.033 | 0.035 | 0.024 | 0.002 | 0.610 | 0.260 | **0.067** |
| K03179 | ubiA | 4-hydroxybenzoate polyprenyltransferase [EC:2.5.1.39] | 0.035 | 0.032 | 0.030 | 0.024 | 0.002 | **0.097** | 0.200 | 0.690 |
| K06295 | gerKA | spore germination protein KA | 0.030 | 0.034 | 0.036 | 0.021 | 0.003 | 0.390 | 0.150 | **0.035** |
| K06284 | abrB | transcriptional pleiotropic regulator of transition state genes | 0.030 | 0.033 | 0.034 | 0.022 | 0.002 | 0.460 | 0.230 | **0.058** |
| **K00626** | **atoB** | **acetyl-CoA C-acetyltransferase [EC:2.3.1.9]** | 0.030 | 0.030 | 0.026 | 0.034 | 0.001 | 0.980 | 0.120 | **0.089** |
| K06397 | spoIIIAH | stage III sporulation protein AH | 0.029 | 0.033 | 0.034 | 0.023 | 0.002 | 0.510 | 0.300 | **0.054** |
| **K10190** | **lacG** | **lactose/L-arabinose transport system permease protein** | 0.028 | 0.031 | 0.034 | 0.023 | 0.002 | 0.740 | 0.330 | **0.077** |
| K03722 | dinG | ATP-dependent DNA helicase DinG [EC:3.6.4.12] | 0.027 | 0.032 | 0.035 | 0.023 | 0.002 | 0.940 | 0.390 | **0.060** |
| K07130 | kynB | arylformamidase [EC:3.5.1.9] | 0.028 | 0.031 | 0.034 | 0.023 | 0.002 | 0.790 | 0.300 | **0.089** |
| K02794 | manX | PTS system- mannose-specific IIB component [EC:2.7.1.191] | 0.022 | 0.028 | 0.024 | 0.042 | 0.004 | **0.056** | **0.007** | 0.130 |
| K08722 | yfbR | 5'-deoxynucleotidase [EC:3.1.3.89] | 0.027 | 0.031 | 0.035 | 0.022 | 0.002 | 0.920 | 0.290 | **0.048** |
| K11072 | potA | spermidine/putrescine transport system ATP-binding protein [EC:3.6.3.31] | 0.031 | 0.027 | 0.024 | 0.033 | 0.002 | 0.910 | 0.380 | **0.035** |
| K06438 | yqfD | similar to stage IV sporulation protein | 0.028 | 0.030 | 0.033 | 0.022 | 0.002 | 0.720 | 0.260 | **0.097** |
| K02795 | manY | PTS system- mannose-specific IIC component | 0.020 | 0.027 | 0.024 | 0.040 | 0.004 | **0.052** | **0.008** | 0.200 |
| K06206 | sfsA | sugar fermentation stimulation protein A | 0.026 | 0.031 | 0.031 | 0.023 | 0.002 | 0.730 | 0.650 | **0.094** |
| K06333 | cotJB | spore coat protein JB | 0.026 | 0.030 | 0.032 | 0.023 | 0.002 | 0.780 | 0.470 | **0.095** |
| K00901 | dgkA | diacylglycerol kinase (ATP) [EC:2.7.1.107] | 0.025 | 0.031 | 0.033 | 0.022 | 0.002 | 0.810 | 0.510 | **0.049** |
| K00857 | tdk | thymidine kinase [EC:2.7.1.21] | 0.025 | 0.023 | 0.024 | 0.038 | 0.003 | **0.072** | 0.120 | **0.055** |
| K06382 | spoIIE | stage II sporulation protein E [EC:3.1.3.16] | 0.026 | 0.029 | 0.033 | 0.022 | 0.002 | 0.840 | 0.280 | **0.078** |
| K01246 | tag | DNA-3-methyladenine glycosylase I [EC:3.2.2.20] | 0.031 | 0.029 | 0.025 | 0.024 | 0.001 | **0.019** | 0.410 | 0.980 |
| K06416 | spoVS | stage V sporulation protein S | 0.025 | 0.030 | 0.032 | 0.022 | 0.002 | 0.950 | 0.470 | **0.068** |
| K01611 | speD | S-adenosylmethionine decarboxylase [EC:4.1.1.50] | 0.027 | 0.029 | 0.033 | 0.019 | 0.002 | 0.660 | 0.170 | **0.072** |
| K02770 | fruA | PTS system- fructose-specific IIC component | 0.026 | 0.026 | 0.023 | 0.034 | 0.002 | 0.350 | **0.058** | **0.044** |
| K02224 | cobB-cbiA | cobyrinic acid c-diamide synthase [EC:6.3.5.9 6.3.5.11] | 0.028 | 0.027 | 0.022 | 0.030 | 0.002 | 0.650 | 0.140 | **0.059** |
| K06283 | spoIIID | putative DeoR family transcriptional regulator stage III sporulation protein D | 0.025 | 0.028 | 0.032 | 0.020 | 0.002 | 0.840 | 0.260 | **0.065** |
| K05995 | pepE | dipeptidase E [EC:3.4.13.21] | 0.029 | 0.029 | 0.025 | 0.022 | 0.001 | **0.042** | 0.620 | 0.560 |
| **K00882** | **fruK** | **1-phosphofructokinase [EC:2.7.1.56]** | 0.024 | 0.025 | 0.023 | 0.033 | 0.002 | 0.220 | **0.072** | 0.120 |
| K07571 | K07571 | S1 RNA binding domain protein | 0.026 | 0.028 | 0.032 | 0.019 | 0.002 | 0.650 | 0.170 | **0.068** |
| K07284 | srtA | sortase A [EC:3.4.22.70] | 0.022 | 0.023 | 0.020 | 0.039 | 0.004 | 0.110 | **0.019** | **0.034** |
| K03697 | clpE | ATP-dependent Clp protease ATP-binding subunit ClpE | 0.025 | 0.029 | 0.031 | 0.018 | 0.003 | 0.490 | 0.230 | **0.038** |
| K06383 | spoIIGA | stage II sporulation protein GA (sporulation sigma-E factor processing peptidase) [EC:3.4.23.-] | 0.024 | 0.027 | 0.031 | 0.019 | 0.002 | 0.770 | 0.230 | **0.057** |
| K08094 | hxlB | 6-phospho-3-hexuloisomerase [EC:5.3.1.27] | 0.022 | 0.030 | 0.030 | 0.019 | 0.002 | 0.730 | 0.660 | **0.049** |
| K11071 | potB | spermidine/putrescine transport system permease protein | 0.026 | 0.024 | 0.021 | 0.030 | 0.002 | 0.820 | 0.170 | **0.023** |
| K01804 | araA | L-arabinose isomerase [EC:5.3.1.4] | 0.021 | 0.021 | 0.022 | 0.036 | 0.003 | **0.087** | 0.120 | 0.130 |
| K07319 | yhdJ | adenine-specific DNA-methyltransferase [EC:2.1.1.72] | 0.025 | 0.030 | 0.029 | 0.016 | 0.003 | 0.250 | 0.390 | **0.059** |
| K01975 | thpR | 3'-cyclic 3'-phosphodiesterase [EC:3.1.4.58] | 0.024 | 0.029 | 0.030 | 0.017 | 0.003 | 0.490 | 0.320 | **0.044** |
| K05833 | ABC.X4.A | putative ABC transport system ATP-binding protein | 0.026 | 0.022 | 0.020 | 0.030 | 0.002 | 0.690 | 0.160 | **0.004** |
| K06196 | ccdA | cytochrome c-type biogenesis protein | 0.024 | 0.027 | 0.031 | 0.017 | 0.002 | 0.650 | 0.160 | **0.049** |
| K09951 | cas2 | CRISPR-associated protein Cas2 | 0.025 | 0.024 | 0.020 | 0.030 | 0.002 | 0.840 | 0.140 | **0.060** |
| K13012 | wbqP | O-antigen biosynthesis protein WbqP | 0.024 | 0.026 | 0.031 | 0.017 | 0.003 | 0.770 | 0.200 | **0.084** |
| **K00248** | **bcd** | **butyryl-CoA dehydrogenase [EC:1.3.8.1]** | 0.029 | 0.024 | 0.019 | 0.026 | 0.002 | 0.170 | 0.780 | **0.043** |
| **K00627** | **aceF** | **pyruvate dehydrogenase E2 component (dihydrolipoamide acetyltransferase) [EC:2.3.1.12]** | 0.022 | 0.027 | 0.029 | 0.018 | 0.002 | 0.880 | 0.440 | **0.082** |
| K05540 | dusB | tRNA-dihydrouridine synthase B [EC:1.-.-.-] | 0.024 | 0.027 | 0.028 | 0.017 | 0.002 | 0.500 | 0.350 | **0.096** |
| K07736 | carD | CarD family transcriptional regulator | 0.023 | 0.023 | 0.020 | 0.029 | 0.002 | 0.490 | **0.077** | **0.064** |
| K08281 | pncA | nicotinamidase/pyrazinamidase [EC:3.5.1.19 3.5.1.-] | 0.022 | 0.019 | 0.020 | 0.033 | 0.003 | 0.160 | 0.250 | **0.084** |
| K02103 | GntR | GntR family transcriptional regulator | 0.023 | 0.027 | 0.027 | 0.017 | 0.002 | 0.480 | 0.390 | **0.085** |
| K11070 | potC | spermidine/putrescine transport system permease protein | 0.024 | 0.023 | 0.020 | 0.027 | 0.001 | 0.840 | 0.130 | **0.045** |
| **K05520** | **pfpI** | **protease I [EC:3.5.1.124]** | 0.026 | 0.026 | 0.023 | 0.019 | 0.001 | **0.069** | 0.540 | 0.440 |
| K11069 | potD | spermidine/putrescine transport system substrate-binding protein | 0.025 | 0.022 | 0.020 | 0.026 | 0.001 | 0.860 | 0.550 | **0.032** |
| **K00161** | **pdhA** | **pyruvate dehydrogenase E1 component alpha subunit [EC:1.2.4.1]** | 0.021 | 0.026 | 0.028 | 0.017 | 0.002 | 0.890 | 0.460 | **0.098** |
| **K00162** | **pdhB** | **pyruvate dehydrogenase E1 component beta subunit [EC:1.2.4.1]** | 0.021 | 0.026 | 0.028 | 0.017 | 0.002 | 0.890 | 0.460 | **0.098** |
| K01583 | E4.1.1.19 | arginine decarboxylase [EC:4.1.1.19] | 0.022 | 0.025 | 0.029 | 0.015 | 0.003 | 0.660 | 0.180 | **0.047** |
| K01989 | ABC.X4.S | putative ABC transport system substrate-binding protein | 0.023 | 0.020 | 0.018 | 0.029 | 0.002 | 0.220 | **0.040** | **0.001** |
| K01667 | tnaA | tryptophanase [EC:4.1.99.1] | 0.026 | 0.024 | 0.023 | 0.017 | 0.002 | **0.062** | 0.120 | 0.310 |
| **K03394** | **cobI-cbiL** | **precorrin-2/cobalt-factor-2 C20-methyltransferase [EC:2.1.1.130 2.1.1.151]** | 0.024 | 0.022 | 0.018 | 0.024 | 0.001 | 0.340 | 0.300 | **0.082** |
| K07775 | resD | two-component system; OmpR family response regulator ResD | 0.021 | 0.024 | 0.028 | 0.016 | 0.002 | 0.910 | 0.330 | **0.082** |
| K00010 | iolG | myo-inositol 2-dehydrogenase / D-chiro-inositol 1-dehydrogenase [EC:1.1.1.18 1.1.1.369] | 0.022 | 0.025 | 0.027 | 0.014 | 0.002 | 0.460 | 0.250 | **0.078** |
| **K10189** | **lacF** | **lactose/L-arabinose transport system permease protein** | 0.021 | 0.024 | 0.027 | 0.016 | 0.002 | 0.800 | 0.340 | **0.085** |
| K02796 | manZ | PTS system- mannose-specific IID component | 0.016 | 0.021 | 0.018 | 0.033 | 0.003 | **0.064** | **0.007** | 0.180 |
| **K00595** | **cobL** | **15-methyltransferase (decarboxylating) [EC:2.1.1.132]** | 0.022 | 0.021 | 0.018 | 0.025 | 0.001 | 0.860 | 0.150 | **0.058** |
| K08234 | yaeR | glyoxylase I family protein | 0.020 | 0.024 | 0.025 | 0.016 | 0.002 | 0.710 | 0.530 | **0.087** |
| K00065 | kduD | 2-dehydro-3-deoxy-D-gluconate 5-dehydrogenase [EC:1.1.1.127] | 0.020 | 0.023 | 0.027 | 0.015 | 0.002 | 0.850 | 0.300 | **0.086** |
| K02173 | yggC | putative kinase | 0.019 | 0.023 | 0.026 | 0.016 | 0.002 | 0.970 | 0.480 | **0.098** |
| K01308 | yqgT | g-D-glutamyl-meso-diaminopimelate peptidase [EC:3.4.19.11] | 0.021 | 0.023 | 0.027 | 0.012 | 0.003 | 0.520 | 0.170 | **0.082** |
| K05593 | aadK | aminoglycoside 6-adenylyltransferase [EC:2.7.7.-] | 0.019 | 0.023 | 0.026 | 0.015 | 0.002 | 0.920 | 0.380 | **0.097** |
| **K05832** | **ABC.X4.P** | **putative ABC transport system permease protein** | 0.021 | 0.018 | 0.017 | 0.026 | 0.002 | 0.220 | **0.072** | **0.002** |
| K02443 | glpP | glycerol uptake operon antiterminator | 0.019 | 0.023 | 0.026 | 0.015 | 0.002 | 0.880 | 0.420 | **0.086** |
| K01626 | aroF | 3-deoxy-7-phosphoheptulonate synthase [EC:2.5.1.54] | 0.019 | 0.020 | 0.016 | 0.028 | 0.002 | 0.430 | **0.057** | **0.099** |
| **K00050** | **ttuD** | **hydroxypyruvate reductase [EC:1.1.1.81]** | 0.019 | 0.023 | 0.025 | 0.014 | 0.002 | 0.730 | 0.420 | **0.082** |
| K12510 | tadB | tight adherence protein B | 0.021 | 0.019 | 0.016 | 0.024 | 0.001 | 0.920 | 0.230 | **0.067** |
| K00091 | E1.1.1.219 | dihydroflavonol-4-reductase [EC:1.1.1.219] | 0.018 | 0.022 | 0.024 | 0.015 | 0.002 | 0.930 | 0.560 | **0.099** |
| K01223 | bglA | 6-phospho-beta-glucosidase [EC:3.2.1.86] | 0.015 | 0.020 | 0.014 | 0.031 | 0.003 | 0.130 | **0.005** | 0.100 |
| K05516 | cbpA | curved DNA-binding protein | 0.016 | 0.016 | 0.019 | 0.029 | 0.003 | **0.089** | 0.250 | 0.210 |
| K07010 | K07010 | putative glutamine amidotransferase | 0.019 | 0.019 | 0.018 | 0.024 | 0.001 | 0.130 | **0.031** | **0.046** |
| K11358 | yhdR | aspartate aminotransferase [EC:2.6.1.1] | 0.020 | 0.019 | 0.017 | 0.024 | 0.001 | 0.530 | 0.260 | **0.097** |
| K07343 | tfoX | DNA transformation protein and related proteins | 0.018 | 0.022 | 0.025 | 0.013 | 0.002 | 0.770 | 0.270 | **0.062** |
| K06402 | spoIVFB | stage IV sporulation protein FB [EC:3.4.24.-] | 0.019 | 0.022 | 0.026 | 0.011 | 0.003 | 0.600 | 0.200 | **0.054** |
| **K00879** | **fucK** | **L-fuculokinase [EC:2.7.1.51]** | 0.019 | 0.022 | 0.025 | 0.011 | 0.003 | 0.490 | 0.250 | **0.085** |
| K09123 | lhpI | cis-L-3-hydroxyproline dehydratase [EC:4.2.1.171] | 0.018 | 0.022 | 0.024 | 0.012 | 0.002 | 0.680 | 0.390 | **0.065** |
| **K02203** | **thrH** | **phosphoserine / homoserine phosphotransferase**  **[EC:3.1.3.3 2.7.1.39]** | 0.019 | 0.016 | 0.015 | 0.025 | 0.002 | 0.320 | 0.200 | **0.017** |
| K02760 | celA | PTS system-cellobiose-specific IIB component [EC:2.7.1.196 2.7.1.205] | 0.014 | 0.019 | 0.015 | 0.027 | 0.003 | 0.110 | **0.007** | 0.190 |
| K02761 | celB | PTS system- cellobiose-specific IIC component | 0.014 | 0.019 | 0.014 | 0.027 | 0.003 | 0.140 | **0.007** | 0.230 |
| **K10540** | **mglB** | **methyl-galactoside transport system substrate-binding protein** | 0.021 | 0.017 | 0.016 | 0.021 | 0.001 | 0.890 | 0.760 | **0.041** |
| K00694 | bcsA | cellulose synthase (UDP-forming) [EC:2.4.1.12] | 0.017 | 0.021 | 0.024 | 0.012 | 0.002 | 0.780 | 0.390 | **0.071** |
| K07402 | xdhC | xanthine dehydrogenase accessory factor | 0.017 | 0.017 | 0.015 | 0.024 | 0.002 | 0.300 | **0.029** | **0.039** |
| K00880 | lyxK | L-xylulokinase [EC:2.7.1.53] | 0.017 | 0.021 | 0.023 | 0.010 | 0.003 | 0.580 | 0.320 | **0.056** |
| K02058 | ABC.SS.S | simple sugar transport system substrate-binding protein | 0.019 | 0.017 | 0.014 | 0.022 | 0.001 | 1.000 | 0.210 | **0.057** |
| **K02007** | **cbiM** | **cobalt/nickel transport system permease protein** | 0.020 | 0.020 | 0.015 | 0.017 | 0.001 | **0.083** | 0.550 | 0.600 |
| K03429 | ugtP | processive 1,2-diacylglycerol beta-glucosyltransferase [EC:2.4.1.315] | 0.017 | 0.020 | 0.023 | 0.011 | 0.002 | 0.710 | 0.290 | **0.079** |
| K02793 | manX | PTS system- mannose-specific IIA component [EC:2.7.1.191] | 0.015 | 0.018 | 0.013 | 0.024 | 0.002 | 0.380 | **0.003** | **0.058** |
| K01971 | ligD | bifunctional non-homologous end joining protein LigD [EC:6.5.1.1] | 0.016 | 0.020 | 0.023 | 0.012 | 0.002 | 0.880 | 0.390 | **0.079** |
| K07813 | agrB | accessory gene regulator B | 0.017 | 0.016 | 0.015 | 0.022 | 0.001 | 0.530 | 0.160 | **0.088** |
| K01698 | hemB | porphobilinogen synthase [EC:4.2.1.24] | 0.021 | 0.018 | 0.015 | 0.016 | 0.001 | **0.080** | 0.510 | 0.400 |
| K00844 | HK | hexokinase [EC:2.7.1.1] | 0.017 | 0.019 | 0.023 | 0.010 | 0.002 | 0.780 | 0.230 | **0.090** |
| K01684 | dgoD | galactonate dehydratase [EC:4.2.1.6] | 0.016 | 0.020 | 0.023 | 0.010 | 0.002 | 0.760 | 0.280 | **0.075** |
| K01512 | acyP | acylphosphatase [EC:3.6.1.7] | 0.015 | 0.018 | 0.014 | 0.022 | 0.002 | 0.570 | **0.010** | 0.160 |
| K02438 | glgX | glycogen debranching enzyme [EC:3.2.1.196] | 0.016 | 0.016 | 0.012 | 0.024 | 0.002 | 0.390 | **0.031** | **0.042** |
| **K01079** | **serB** | **phosphoserine phosphatase [EC:3.1.3.3]** | 0.014 | 0.013 | 0.016 | 0.025 | 0.002 | **0.078** | 0.300 | 0.180 |
| K06606 | iolI | 2-keto-myo-inositol isomerase [EC:5.3.99.11] | 0.016 | 0.020 | 0.022 | 0.010 | 0.002 | 0.690 | 0.310 | **0.089** |
| K00068 | srlD | sorbitol-6-phosphate 2-dehydrogenase [EC:1.1.1.140] | 0.016 | 0.019 | 0.022 | 0.010 | 0.002 | 0.770 | 0.310 | **0.098** |
| K07455 | recT | recombination protein RecT | 0.016 | 0.019 | 0.022 | 0.010 | 0.002 | 0.700 | 0.280 | **0.094** |
| K05814 | ugpA | sn-glycerol 3-phosphate transport system permease protein | 0.016 | 0.019 | 0.023 | 0.010 | 0.002 | 0.780 | 0.300 | **0.073** |
| **K03518** | **coxS** | **aerobic carbon-monoxide dehydrogenase small subunit [EC:1.2.5.3]** | 0.016 | 0.016 | 0.014 | 0.021 | 0.001 | 0.450 | **0.019** | **0.020** |
| K06972 | PreP | presequence protease [EC:3.4.24.-] | 0.017 | 0.015 | 0.014 | 0.021 | 0.001 | 0.490 | 0.160 | **0.023** |
| K05884 | comC | L-2-hydroxycarboxylate dehydrogenase (NAD+) [EC:1.1.1.337] | 0.016 | 0.020 | 0.023 | 0.009 | 0.002 | 0.710 | 0.310 | **0.065** |
| **K03928** | **yvaK** | **carboxylesterase [EC:3.1.1.1]** | 0.016 | 0.019 | 0.022 | 0.010 | 0.002 | 0.720 | 0.270 | **0.086** |
| K03698 | cbf | 3'-5' exoribonuclease [EC:3.1.-.-] | 0.016 | 0.016 | 0.012 | 0.022 | 0.002 | 0.680 | **0.003** | **0.003** |
| **K13641** | **iclR** | **IclR family transcriptional regulator; acetate operon repressor** | 0.015 | 0.019 | 0.022 | 0.010 | 0.002 | 0.760 | 0.320 | **0.071** |
| K02757 | bglF | PTS system-beta-glucoside-specific IIC component | 0.014 | 0.016 | 0.014 | 0.023 | 0.002 | **0.070** | **0.011** | **0.067** |
| **K01492** | **purNH** | **phosphoribosylglycinamide/phosphoribosylaminoimidazolecarboxamide formyltransferase [EC:2.1.2.2 2.1.2.3]** | 0.016 | 0.019 | 0.022 | 0.009 | 0.002 | 0.760 | 0.280 | **0.082** |
| K01267 | DNPEP | aspartyl aminopeptidase [EC:3.4.11.21] | 0.016 | 0.015 | 0.013 | 0.021 | 0.001 | 0.340 | **0.099** | **0.023** |
| K07217 | K07217 | Mn-containing catalase | 0.015 | 0.019 | 0.022 | 0.010 | 0.002 | 0.850 | 0.320 | **0.070** |
| K10979 | ku | DNA end-binding protein Ku | 0.015 | 0.019 | 0.022 | 0.010 | 0.002 | 0.860 | 0.330 | **0.074** |
| K02192 | bfd | bacterioferritin-associated ferredoxin | 0.015 | 0.019 | 0.022 | 0.010 | 0.002 | 0.850 | 0.320 | **0.074** |
| K03079 | sgaU | L-ribulose-5-phosphate 3-epimerase [EC:5.1.3.22] | 0.015 | 0.018 | 0.022 | 0.009 | 0.002 | 0.790 | 0.290 | **0.081** |
| K07318 | K07318 | adenine-specific DNA-methyltransferase [EC:2.1.1.72] | 0.015 | 0.018 | 0.022 | 0.009 | 0.002 | 0.820 | 0.300 | **0.074** |
| K02076 | zurR | Fur family transcriptional regulator zinc uptake regulator | 0.015 | 0.018 | 0.022 | 0.009 | 0.002 | 0.810 | 0.310 | **0.071** |
| K05346 | deoR | deoxyribonucleoside regulator | 0.015 | 0.018 | 0.022 | 0.009 | 0.002 | 0.800 | 0.310 | **0.072** |
| **K14084** | **mttC** | **trimethylamine corrinoid protein** | 0.015 | 0.018 | 0.022 | 0.009 | 0.002 | 0.820 | 0.300 | **0.075** |
| K02436 | gatR | galactitol utilization operon repressor | 0.014 | 0.018 | 0.022 | 0.009 | 0.002 | 0.820 | 0.300 | **0.074** |
| K10212 | crtO | glycosyl-4 | 0.014 | 0.018 | 0.022 | 0.009 | 0.002 | 0.820 | 0.300 | **0.075** |
| K00926 | arcC | carbamate kinase [EC:2.7.2.2] | 0.014 | 0.015 | 0.013 | 0.020 | 0.002 | 0.300 | **0.056** | 0.110 |
| K07023 | K07023 | putative hydrolases of HD superfamily | 0.016 | 0.014 | 0.011 | 0.019 | 0.002 | 0.830 | **0.063** | **0.009** |
| K02759 | celC | PTS system- cellobiose-specific IIA component [EC:2.7.1.196 2.7.1.205] | 0.012 | 0.015 | 0.013 | 0.018 | 0.001 | 0.290 | **0.056** | 0.490 |
| K03700 | recU | recombination protein U | 0.014 | 0.013 | 0.010 | 0.020 | 0.002 | 0.310 | **0.009** | **0.008** |
| K03484 | LacI | sucrose operon repressor | 0.015 | 0.013 | 0.011 | 0.018 | 0.001 | 0.840 | **0.094** | **0.011** |
| K01715 | crt | enoyl-CoA hydratase [EC:4.2.1.17] | 0.017 | 0.013 | 0.010 | 0.015 | 0.001 | 0.160 | 0.630 | **0.006** |
| K03785 | aroD | 3-dehydroquinate dehydratase I [EC:4.2.1.10] | 0.015 | 0.013 | 0.011 | 0.016 | 0.001 | 0.930 | 0.400 | **0.087** |
| K06200 | cstA | carbon starvation protein | 0.013 | 0.013 | 0.011 | 0.017 | 0.001 | 0.400 | **0.048** | **0.039** |
| K01673 | cynT | carbonic anhydrase [EC:4.2.1.1] | 0.014 | 0.012 | 0.010 | 0.017 | 0.001 | 0.810 | 0.220 | **0.083** |
| K05846 | opuBD | osmoprotectant transport system permease protein | 0.013 | 0.014 | 0.011 | 0.015 | 0.001 | 0.800 | **0.041** | 0.160 |
| K01788 | nanE | N-acylglucosamine-6-phosphate 2-epimerase [EC:5.1.3.9] | 0.011 | 0.012 | 0.011 | 0.017 | 0.001 | **0.068** | **0.028** | **0.056** |
| K02188 | cbiD | cobalt-precorrin-5B (C1)-methyltransferase [EC:2.1.1.195] | 0.012 | 0.013 | 0.010 | 0.015 | 0.001 | 0.890 | **0.075** | 0.180 |
| K10254 | ohyA | oleate hydratase [EC:4.2.1.53] | 0.011 | 0.013 | 0.011 | 0.015 | 0.001 | 0.620 | **0.033** | 0.380 |
| K04047 | dps | starvation-inducible DNA-binding protein | 0.013 | 0.010 | 0.009 | 0.017 | 0.002 | 0.720 | 0.340 | **0.037** |
| K02774 | gatB | PTS system- galactitol-specific IIB component [EC:2.7.1.200] | 0.010 | 0.011 | 0.010 | 0.018 | 0.002 | **0.095** | **0.048** | 0.150 |
| K07313 | pphA | serine/threonine protein phosphatase 1 [EC:3.1.3.16] | 0.010 | 0.013 | 0.009 | 0.016 | 0.001 | 0.550 | **0.021** | 0.380 |
| K03488 | bglG | beta-glucoside operon transcriptional antiterminator | 0.009 | 0.011 | 0.010 | 0.017 | 0.002 | **0.044** | **0.017** | 0.100 |
| K01531 | mgtA | Mg2+-importing ATPase [EC:3.6.3.2] | 0.012 | 0.011 | 0.010 | 0.013 | 0.001 | 0.930 | 0.290 | **0.035** |
| K01639 | nanA | N-acetylneuraminate lyase [EC:4.1.3.3] | 0.011 | 0.010 | 0.008 | 0.017 | 0.002 | 0.350 | **0.091** | **0.031** |
| K00526 | nrdB | ribonucleoside-diphosphate reductase beta chain [EC:1.17.4.1] | 0.008 | 0.011 | 0.011 | 0.016 | 0.001 | **0.017** | **0.027** | 0.390 |
| K00284 | gltS | glutamate synthase (ferredoxin) [EC:1.4.7.1] | 0.011 | 0.011 | 0.008 | 0.015 | 0.001 | 0.580 | **0.034** | **0.049** |
| K01008 | selD | selenide, water dikinase [EC:2.7.9.3] | 0.012 | 0.010 | 0.009 | 0.013 | 0.001 | 0.670 | 0.260 | **0.029** |
| K07404 | pgl | 6-phosphogluconolactonase [EC:3.1.1.31] | 0.011 | 0.012 | 0.008 | 0.014 | 0.001 | 0.920 | **0.023** | **0.069** |
| K05847 | opuA | osmoprotectant transport system ATP-binding protein | 0.011 | 0.012 | 0.009 | 0.012 | 0.001 | 0.460 | **0.083** | 0.260 |
| K07050 | ALAX | misacylated tRNA(Ala) deacylase [EC:3.1.1.-] | 0.011 | 0.011 | 0.008 | 0.014 | 0.001 | 0.760 | **0.070** | **0.047** |
| K02822 | ulaB | PTS system- ascorbate-specific IIB component [EC:2.7.1.194] | 0.008 | 0.010 | 0.010 | 0.014 | 0.001 | **0.088** | **0.076** | 0.650 |
| K03799 | htpX | heat shock protein HtpX [EC:3.4.24.-] | 0.013 | 0.012 | 0.009 | 0.009 | 0.001 | **0.048** | 0.610 | 0.530 |
| K02765 | gamP | PTS system- D-glucosamine-specific IIC component | 0.010 | 0.010 | 0.008 | 0.014 | 0.001 | 0.240 | **0.022** | **0.025** |
| K02775 | gatC | PTS system- galactitol-specific IIC component | 0.009 | 0.010 | 0.009 | 0.015 | 0.001 | 0.120 | **0.081** | 0.180 |
| K02773 | gatA | PTS system- galactitol-specific IIA component [EC:2.7.1.200] | 0.008 | 0.010 | 0.009 | 0.015 | 0.001 | **0.080** | **0.057** | 0.300 |
| **K01007** | **pps** | **pyruvate, water dikinase [EC:2.7.9.2]** | 0.012 | 0.012 | 0.009 | 0.009 | 0.001 | **0.017** | 0.990 | 0.770 |
| K01198 | xynB | 4-beta-xylosidase [EC:3.2.1.37] | 0.010 | 0.010 | 0.007 | 0.015 | 0.001 | 0.400 | **0.063** | **0.057** |
| **K00172** | **porG** | **pyruvate ferredoxin oxidoreductase gamma subunit [EC:1.2.7.1]** | 0.012 | 0.011 | 0.009 | 0.009 | 0.001 | **0.076** | 0.420 | 0.770 |
| K00104 | glcD | glycolate oxidase [EC:1.1.3.15] | 0.013 | 0.009 | 0.008 | 0.010 | 0.001 | 0.190 | 0.700 | **0.085** |
| **K00169** | **porA** | **pyruvate ferredoxin oxidoreductase alpha subunit [EC:1.2.7.1]** | 0.012 | 0.011 | 0.009 | 0.009 | 0.001 | **0.076** | 0.440 | 0.790 |
| K01138 | K01138 | uncharacterized sulfatase [EC:3.1.6.-] | 0.012 | 0.012 | 0.007 | 0.009 | 0.001 | **0.009** | 0.320 | 0.390 |
| Others |  |  | 75.04 | 74.70 | 74.391 | 75.08 | 0.003 | 0.141 | 0.542 | 0.771 |

Data is shown as LS Means with standard errors. Number of steers = 8

Bold P-values indicate genes that tend to differ (0.05 < P < 0.1) and significantly differ (P < 0.05)

Genes in bold type are associated with methane production either directly or indirectly.

TMR, Total mixed ration; SF, separate feeding; FS, feeding system

**Supplementary Table S5. Relative abundance of gene pathways of the rumen microbiota in Hanwoo and Holstein steers fed by two feeding system representing > 0.01% of total predicted pathways by PICRUSt that tend to differ (0.05 < P < 0.1) and significantly differ (P < 0.05)**

| **KEGG Pathway** | **HN** | | **HS** | | **SEM** | **P Value** | | |
| --- | --- | --- | --- | --- | --- | --- | --- | --- |
|  | **TMR** | **SF** | **TMR** | **SF** |  | **Breed** | **FS** | **Breed × FS** |
| Purine metabolism | 2.378 | 2.373 | 2.400 | 2.413 | 0.008 | **0.090** | 0.830 | 0.620 |
| Phosphonate and phosphinate metabolism | 1.968 | 1.950 | 1.978 | 2.005 | 0.010 | 0.710 | 0.280 | **0.083** |
| Amino sugar and nucleotide sugar metabolism | 1.393 | 1.398 | 1.398 | 1.473 | 0.017 | **0.076** | **0.076** | 0.110 |
| Dioxin degradation | 1.375 | 1.365 | 1.378 | 1.403 | 0.007 | 0.720 | 0.480 | **0.093** |
| Methane metabolism | 1.358 | 1.400 | 1.358 | 1.275 | 0.023 | **0.050** | 0.170 | **0.050** |
| Unknown function | 1.233 | 1.248 | 1.248 | 1.210 | 0.008 | 0.150 | 0.150 | **0.004** |
| Homologous recombination | 1.095 | 1.085 | 1.105 | 1.085 | 0.004 | 0.470 | **0.047** | 0.470 |
| Pyruvate metabolism | 1.078 | 1.113 | 1.110 | 1.025 | 0.018 | 0.380 | 0.420 | **0.069** |
| Translation proteins | 1.010 | 1.015 | 1.025 | 0.988 | 0.007 | 0.480 | 0.080 | **0.028** |
| Lysine biosynthesis | 0.863 | 0.868 | 0.873 | 0.835 | 0.007 | 0.330 | 0.170 | **0.080** |
| Lipid biosynthesis proteins | 0.673 | 0.673 | 0.683 | 0.653 | 0.005 | 0.550 | **0.087** | **0.087** |
| Bacterial secretion system | 0.648 | 0.658 | 0.675 | 0.633 | 0.008 | 0.920 | 0.200 | **0.049** |
| Terpenoid backbone biosynthesis | 0.645 | 0.630 | 0.633 | 0.645 | 0.003 | 0.860 | 0.860 | **0.069** |
| Butanoate metabolism | 0.638 | 0.640 | 0.630 | 0.605 | 0.007 | **0.034** | 0.230 | 0.150 |
| Nicotinate and nicotinamide metabolism | 0.518 | 0.520 | 0.538 | 0.530 | 0.004 | **0.080** | 0.760 | 0.540 |
| Fatty acid biosynthesis | 0.513 | 0.523 | 0.515 | 0.488 | 0.007 | 0.130 | 0.400 | **0.089** |
| Streptomycin biosynthesis | 0.380 | 0.375 | 0.388 | 0.365 | 0.004 | 0.840 | **0.038** | 0.160 |
| Selenocompound metabolism | 0.363 | 0.350 | 0.358 | 0.368 | 0.003 | 0.200 | 0.790 | **0.031** |
| Polycyclic aromatic hydrocarbon degradation | 0.333 | 0.333 | 0.308 | 0.395 | 0.016 | **0.089** | 0.017 | **0.089** |
| Photosynthesis proteins | 0.333 | 0.330 | 0.305 | 0.393 | 0.016 | 0.410 | **0.067** | **0.067** |
| C_5_ Branched dibasic acid metabolism | 0.318 | 0.323 | 0.313 | 0.303 | 0.004 | **0.083** | 0.710 | 0.280 |
| Sulfur metabolism | 0.245 | 0.233 | 0.233 | 0.250 | 0.004 | 0.750 | 0.750 | **0.075** |
| Photosynthesis | 0.190 | 0.218 | 0.190 | 0.298 | 0.022 | 0.410 | **0.060** | **0.048** |
| Restriction enzyme | 0.220 | 0.210 | 0.218 | 0.205 | 0.003 | 0.420 | **0.028** | 0.790 |
| Chloroalkane and chloroalkene degradation | 0.193 | 0.203 | 0.200 | 0.170 | 0.006 | 0.280 | 0.390 | **0.098** |
| Naphthalene degradation | 0.170 | 0.178 | 0.190 | 0.165 | 0.005 | 0.690 | 0.360 | **0.099** |
| Inorganic ion transport and metabolism | 0.168 | 0.173 | 0.178 | 0.168 | 0.002 | 0.530 | 0.530 | **0.073** |
| D Glutamine and D glutamate metabolism | 0.173 | 0.170 | 0.173 | 0.163 | 0.002 | 0.230 | **0.058** | 0.230 |
| Lipid metabolism | 0.140 | 0.143 | 0.153 | 0.140 | 0.003 | 0.230 | 0.230 | **0.082** |
| Peptidases | 0.128 | 0.123 | 0.133 | 0.120 | 0.002 | **0.095** | 0.790 | 0.230 |
| PPAR signaling pathway | 0.103 | 0.105 | 0.103 | 0.115 | 0.003 | 0.740 | **0.032** | 0.320 |
| Butirosin and neomycin biosynthesis | 0.095 | 0.103 | 0.108 | 0.095 | 0.003 | 0.540 | 0.540 | **0.026** |
| Protein processing in endoplasmic reticulum | 0.090 | 0.093 | 0.095 | 0.085 | 0.002 | 0.610 | 0.140 | **0.023** |
| Phosphotransferase system PTS | 0.065 | 0.070 | 0.075 | 0.055 | 0.004 | **0.065** | **0.005** | **0.065** |
| DNA replication proteins | 0.058 | 0.065 | 0.068 | 0.050 | 0.003 | **0.093** | 0.510 | 0.140 |
| Alzheimer's disease | 0.053 | 0.058 | 0.053 | 0.058 | 0.001 | 1.000 | **0.069** | 1.000 |
| Glycan biosynthesis and metabolism | 0.048 | 0.045 | 0.050 | 0.040 | 0.002 | 0.530 | **0.007** | **0.073** |
| Nitrotoluene degradation | 0.045 | 0.045 | 0.038 | 0.040 | 0.002 | **0.070** | 0.700 | 0.700 |
| Lipoic acid metabolism | 0.045 | 0.045 | 0.043 | 0.033 | 0.003 | **0.017** | **0.089** | **0.089** |
| Primary immunodeficiency | 0.040 | 0.040 | 0.035 | 0.045 | 0.002 | 1.000 | **0.031** | **0.031** |
| Retinol metabolism | 0.038 | 0.043 | 0.043 | 0.030 | 0.003 | 0.430 | 0.430 | **0.083** |
| Arachidonic acid metabolism | 0.035 | 0.030 | 0.033 | 0.048 | 0.003 | 0.100 | 0.260 | **0.035** |
| Nucleotide metabolism | 0.040 | 0.038 | 0.033 | 0.030 | 0.002 | **0.047** | 0.470 | 1.000 |
| Drug metabolism cytochrome P450 | 0.033 | 0.038 | 0.038 | 0.025 | 0.003 | 0.450 | 0.450 | **0.096** |
| Metabolism of xenobiotics by cytochrome P450 | 0.033 | 0.038 | 0.038 | 0.025 | 0.003 | 0.450 | 0.450 | **0.096** |
| Germination | 0.033 | 0.035 | 0.035 | 0.025 | 0.002 | 0.300 | 0.300 | **0.096** |
| Proximal tubule bicarbonate reclamation | 0.028 | 0.033 | 0.033 | 0.025 | 0.002 | 0.640 | 0.640 | **0.033** |
| Electron transfer carriers | 0.015 | 0.020 | 0.020 | 0.013 | 0.002 | 0.660 | 0.660 | **0.045** |
| Carotenoid biosynthesis | 0.010 | 0.015 | 0.015 | 0.008 | 0.002 | 0.700 | 0.700 | **0.070** |
| Others | 78.368 | 78.233 | 78.173 | 78.395 | 0.003 | 0.841 | 0.774 | 0.551 |

Data is shown as LS Means with standard errors. Number of steers = 8

Bold P-values indicate gene pathways that tend to differ (0.05 < P < 0.1) and significantly differ (P < 0.05)

TMR, Total mixed ration; SF, separate feeding; FS, feeding system

**Supplementary Table S6. Relative abundance of microbial genes strongly associated (P < 0.01) with methane production as identified by the Kendall rank correlation analysis.**

| **Gene ID** | **Gene Label** | **Gene Definition** | **Kendall's correlation** | | **Relative abundance (%)** | | | | **SEM** | **P value** | | | **Gene product/module** |
| --- | --- | --- | --- | --- | --- | --- | --- | --- | --- | --- | --- | --- | --- |
|  |  |  |  |  | **HN** | | **HS** | |  |  |  |  |  |
|  |  |  | **τ** | **P -Value** | **TMR** | **SF** | **TMR** | **SF** |  | **Breed** | **FS** | **Breed × FS** |  |
| K00169 | porA | pyruvate ferredoxin oxidoreductase alpha subunit [EC:1.2.7.1] | 0.49 | 0.008 | 0.012 | 0.011 | 0.009 | 0.009 | 0.001 | **0.076** | 0.440 | 0.790 | Acetyl-CoA, CO_2_, H_2_ (P) |
| K00172 | porG | pyruvate ferredoxin oxidoreductase gamma subunit [EC:1.2.7.1] | 0.50 | 0.006 | 0.012 | 0.011 | 0.009 | 0.009 | 0.001 | **0.076** | 0.420 | 0.770 | Acetyl-CoA, CO_2_, H_2_ (P) |
| K01007 | pps | pyruvate, water dikinase [EC:2.7.9.2] | 0.44 | 0.002 | 0.012 | 0.012 | 0.009 | 0.009 | 0.001 | **0.017** | 0.990 | 0.770 | Phosphoenolpyruvate (P), H_2_ (U) |
| K05884 | comC | L-2-hydroxycarboxylate dehydrogenase (NAD+) [EC:1.1.1.337] | 0.92 | <0.001 | 0.016 | 0.020 | 0.023 | 0.009 | 0.002 | 0.710 | 0.310 | **0.065** | 2-oxocarboxylate, H_2_ (P) |
| K08094 | hxlB | 6-phospho-3-hexuloisomerase [EC:5.3.1.27] | 0.93 | <0.001 | 0.022 | 0.030 | 0.030 | 0.019 | 0.002 | 0.730 | 0.660 | **0.049** | Fructose 6-phosphate (P) |
| K14084 | mttC | trimethylamine corrinoid protein | 0.76 | <0.001 | 0.015 | 0.018 | 0.022 | 0.009 | 0.002 | 0.820 | 0.300 | **0.075** | Methyl-CoM (P) |
| K02007 | cbiM | cobalt/nickel transport system permease protein | 0.39 | 0.004 | 0.020 | 0.020 | 0.015 | 0.017 | 0.001 | **0.083** | 0.550 | 0.600 | ABC transporters |
| K02117 | atpA | V/A-type H+/Na+-transporting ATPase subunit A [EC:7.1.2.2 7.2.2.1] | 0.47 | 0.001 | 0.065 | 0.064 | 0.065 | 0.048 | 0.003 | 0.100 | **0.076** | 0.100 | Energy synthesis, H_2_ transfer |
| K02118 | atpB | V/A-type H+/Na+-transporting ATPase subunit B | 0.47 | 0.001 | 0.065 | 0.064 | 0.066 | 0.052 | 0.003 | 0.130 | **0.075** | 0.110 | Energy synthesis, H_2_ transfer |
| K02120 | atpD | V/A-type H+/Na+-transporting ATPase subunit D | 0.44 | 0.008 | 0.064 | 0.064 | 0.064 | 0.051 | 0.003 | 0.130 | **0.093** | 0.100 | Energy synthesis, H_2_ transfer |
| K02121 | atpE | V/A-type H+/Na+-transporting ATPase subunit E | 0.43 | 0.002 | 0.064 | 0.063 | 0.064 | 0.048 | 0.003 | **0.077** | **0.054** | **0.073** | Energy synthesis, H_2_ transfer |
| K02124 | atpK | V/A-type H+/Na+-transporting ATPase subunit K | 0.44 | 0.008 | 0.064 | 0.064 | 0.064 | 0.051 | 0.003 | 0.120 | **0.093** | 0.100 | Energy synthesis, H_2_ transfer |
| K00940 | ndk | nucleoside-diphosphate kinase [EC:2.7.4.6] | 0.49 | 0.008 | 0.035 | 0.033 | 0.031 | 0.023 | 0.002 | **0.084** | 0.250 | 0.430 | Nucleoside triphosphate, ADP (P) |
| K01153 | hsdR | type I restriction enzyme R subunit [EC:3.1.21.3] | 0.55 | 0.003 | 0.098 | 0.093 | 0.094 | 0.088 | 0.002 | **0.052** | **0.020** | 0.590 | Acting on ester bonds |
| K01246 | tag | DNA-3-methyladenine glycosylase I [EC:3.2.2.20] | 0.54 | 0.004 | 0.031 | 0.029 | 0.025 | 0.024 | 0.001 | **0.019** | 0.410 | 0.980 | Hydrolysing N-glycosyl compounds |
| K01251 | ahcY | adenosylhomocysteinase [EC:3.3.1.1] | 0.53 | 0.004 | 0.040 | 0.039 | 0.035 | 0.027 | 0.003 | **0.076** | 0.290 | 0.490 | L-homocysteine, Adenosine(P), H_2_ (U) |
| K01667 | tnaA | tryptophanase [EC:4.1.99.1] | 0.61 | 0.001 | 0.026 | 0.024 | 0.023 | 0.017 | 0.002 | **0.062** | 0.120 | 0.310 | Pyruvate, NH_3_ (P), H_2_ (U) |
| K01703 | leuC | 3-isopropylmalate/(R)-2-methylmalate dehydratase large subunit [EC:4.2.1.33 4.2.1.35] | 0.53 | 0.004 | 0.157 | 0.157 | 0.152 | 0.144 | 0.003 | **0.057** | 0.320 | 0.320 | Pyruvate (U) |
| K01704 | leuD | 3-isopropylmalate/(R)-2-methylmalate dehydratase small subunit [EC:4.2.1.33 4.2.1.35] | 0.50 | 0.007 | 0.153 | 0.153 | 0.150 | 0.141 | 0.003 | **0.081** | 0.330 | 0.330 | Pyruvate (U) |
| K03150 | thiH | 2-iminoacetate synthase [EC:4.1.99.19] | 0.50 | 0.006 | 0.067 | 0.064 | 0.065 | 0.051 | 0.003 | **0.052** | **0.038** | 0.170 | 2-iminoacetate, 4-methylphenol, L-methionine, H^+^ (P) |
| K03179 | ubiA | 4-hydroxybenzoate polyprenyltransferase [EC:2.5.1.39] | 0.49 | 0.008 | 0.035 | 0.032 | 0.030 | 0.024 | 0.002 | **0.097** | 0.200 | 0.690 | 4-hydroxy-3-polyprenylbenzoate (P) |
| K03427 | hsdM | type I restriction enzyme M protein [EC:2.1.1.72] | 0.62 | 0.001 | 0.123 | 0.117 | 0.116 | 0.103 | 0.004 | **0.041** | **0.045** | 0.450 | S-adenosyl-L-homocysteine, N6-methyladenine in DNA |
| K05995 | pepE | dipeptidase E [EC:3.4.13.21] | 0.50 | 0.006 | 0.029 | 0.029 | 0.025 | 0.022 | 0.001 | **0.042** | 0.620 | 0.560 | Acting on peptide bonds |
| K00873 | pyk | pyruvate kinase [EC:2.7.1.40] | -0.41 | 0.003 | 0.084 | 0.087 | 0.086 | 0.089 | 0.001 | 0.220 | **0.015** | 0.840 | Phosphoenolpyruvate (P), Pyruvate (U) |
| K00925 | ackA | acetate kinase [EC:2.7.2.1] | -0.46 | 0.001 | 0.088 | 0.087 | 0.088 | 0.091 | 0.001 | **0.034** | 0.330 | **0.092** | Acetyl phosphate (P), Acetate (U) |
| K01079 | serB | phosphoserine phosphatase [EC:3.1.3.3] | -0.44 | 0.002 | 0.014 | 0.013 | 0.016 | 0.025 | 0.002 | **0.078** | 0.300 | 0.180 | Serine (P), H_2_ (U) |
| K01689 | ENO | enolase [EC:4.2.1.11] | -0.50 | 0.006 | 0.087 | 0.086 | 0.088 | 0.091 | 0.001 | **0.070** | 0.700 | 0.200 | Phosphoenolpyruvate, H_2_ (P) |
| K02203 | thrH | phosphoserine / homoserine phosphotransferase [EC:3.1.3.3 2.7.1.39] | -0.54 | 0.003 | 0.019 | 0.016 | 0.015 | 0.025 | 0.002 | 0.320 | 0.200 | **0.017** | Serine (P), H_2_ (U) |
| K00075 | murB | UDP-N-acetylmuramate dehydrogenase [EC:1.3.1.98] | -0.51 | 0.006 | 0.084 | 0.084 | 0.087 | 0.088 | 0.001 | **0.085** | 0.700 | 0.690 | UDP-N-acetyl-3-O-(1-carboxyvinyl)-alpha-D-glucosamine |
| K00266 | gltD | glutamate synthase (NADPH) small chain [EC:1.4.1.13] | -0.48 | 0.010 | 0.243 | 0.239 | 0.247 | 0.261 | 0.004 | **0.056** | 0.450 | 0.180 | L-glutamine, NH_3_, H_2_ (P) |
| K00526 | nrdB | ribonucleoside-diphosphate reductase beta chain [EC:1.17.4.1] | -0.53 | 0.004 | 0.008 | 0.011 | 0.011 | 0.016 | 0.001 | **0.017** | **0.027** | 0.390 | Ribonucleoside 5'-diphosphate, H_2_ (U) |
| K00599 | METTL6 | methyltransferase-like protein 6 [EC:2.1.1.-] | -0.49 | 0.008 | 0.169 | 0.177 | 0.171 | 0.186 | 0.003 | 0.220 | **0.020** | 0.450 | N.I |
| K00604 | fmt | methionyl-tRNA formyltransferase [EC:2.1.2.9] | -0.51 | 0.006 | 0.090 | 0.090 | 0.090 | 0.092 | 0.001 | 0.120 | **0.009** | 0.120 | Tetrahydrofolate (P) |
| K00759 | apt | adenine phosphoribosyltransferase [EC:2.4.2.7] | -0.61 | 0.001 | 0.064 | 0.064 | 0.068 | 0.073 | 0.002 | **0.045** | 0.370 | 0.400 | Adenine adenine(P) |
| K00763 | pncB | nicotinate phosphoribosyltransferase [EC:6.3.4.21] | -0.53 | 0.004 | 0.060 | 0.063 | 0.065 | 0.071 | 0.002 | **0.070** | 0.210 | 0.520 | Beta-nicotinate D-ribonucleotide (P), H_2_ (U) |
| K00942 | gmk | guanylate kinase [EC:2.7.4.8] | -0.55 | 0.003 | 0.087 | 0.088 | 0.089 | 0.090 | 0.000 | **0.042** | 0.170 | 0.550 | ADP (P) |
| K01129 | dgt | dGTPase [EC:3.1.5.1] | -0.53 | 0.004 | 0.063 | 0.062 | 0.066 | 0.072 | 0.002 | **0.056** | 0.450 | 0.350 | Deoxyguanosine (P), H_2_ (U) |
| K01874 | metG | methionyl-tRNA synthetase [EC:6.1.1.10] | -0.64 | 0.001 | 0.092 | 0.093 | 0.094 | 0.098 | 0.001 | **0.039** | **0.096** | 0.310 | L-methionyl-tRNA(Met) |
| K01915 | glnA | glutamine synthetase [EC:6.3.1.2] | -0.60 | 0.001 | 0.149 | 0.151 | 0.153 | 0.160 | 0.002 | **0.086** | 0.160 | 0.430 | L-glutamine (P), NH_3_ (U) |
| K02335 | polA | DNA polymerase I [EC:2.7.7.7] | -0.57 | 0.002 | 0.088 | 0.088 | 0.089 | 0.090 | 0.001 | **0.039** | 0.380 | 0.610 | Diphosphate (P) |
| K02356 | efp | elongation factor P | -0.53 | 0.004 | 0.090 | 0.090 | 0.090 | 0.093 | 0.001 | **0.051** | **0.060** | 0.150 | N.I |
| K02795 | manY | PTS system- mannose-specific IIC component | -0.49 | 0.008 | 0.020 | 0.027 | 0.024 | 0.040 | 0.004 | **0.052** | **0.008** | 0.200 | Fructose and mannose metabolism |
| K02822 | ulaB | PTS system- ascorbate-specific IIB component [EC:2.7.1.194] | -0.51 | 0.006 | 0.008 | 0.010 | 0.010 | 0.014 | 0.001 | **0.088** | **0.076** | 0.650 | L-ascorbate 6-phosphate (P) |
| K03402 | ahrC | transcriptional regulator of arginine metabolism | -0.49 | 0.008 | 0.053 | 0.053 | 0.057 | 0.061 | 0.002 | **0.092** | 0.630 | 0.600 | Genetic information processing |
| K03553 | recA | recombination protein RecA | -0.57 | 0.002 | 0.088 | 0.088 | 0.089 | 0.090 | 0.000 | **0.055** | **0.096** | 0.230 | Gyrase-protecting protein |
| K03574 | mutT | 8-oxo-dGTP diphosphatase [EC:3.6.1.55] | -0.48 | 0.010 | 0.086 | 0.086 | 0.084 | 0.096 | 0.002 | 0.140 | **0.038** | **0.037** | Diphosphate (P), H_2_ (U) |
| K06153 | bacA | undecaprenyl-diphosphatase [EC:3.6.1.27] | -0.48 | 0.009 | 0.083 | 0.083 | 0.085 | 0.086 | 0.001 | **0.018** | 0.410 | 0.600 | Peptidoglycan biosynthesis |
| K06215 | pdx1 | pyridoxal 5'-phosphate synthase pdxS subunit [EC:4.3.3.6] | -0.48 | 0.010 | 0.042 | 0.044 | 0.050 | 0.048 | 0.002 | **0.044** | 0.880 | 0.600 | L-glutamate, NH_3_, H_2_O (P) |
| K07173 | luxS | S-ribosylhomocysteine lyase [EC:4.4.1.21] | -0.56 | 0.002 | 0.046 | 0.047 | 0.052 | 0.058 | 0.002 | **0.044** | 0.390 | 0.510 | Auto Inducer- 2 (P) |
| K09888 | zapA | cell division protein ZapA | -0.54 | 0.003 | 0.046 | 0.048 | 0.051 | 0.053 | 0.001 | **0.084** | 0.540 | 0.950 | N.I |
| K11068 | hlyIII | hemolysin III | -0.64 | 0.001 | 0.045 | 0.047 | 0.053 | 0.060 | 0.003 | **0.022** | 0.260 | 0.470 | Signaling and cellular processes |

Data is shown as LS Means with standard errors (n = 16 observations for correlation). Number of steers = 8

The associations were considered significant only when Kendall’s P < 0.01

Bold P-values indicate genes that tend to differ (0.05 < P < 0.1) and significantly differ (P < 0.05) between groups

TMR, Total mixed ration; SF, separate feeding; FS, feeding system; U, Utilizer; P, Producer; ADP, Adenosine diphosphate; N.I, No information

**Supplementary Table S7. Associations of rumen microbes with** CH_4_ **yield, metabolic pathways and metabolites observed in the rumen of the steers. (n = 16 observations)**

| 1. **Kendall's Coefficient (τ)** | ***Methanobrevibacter*** | | | ***VadinCA11*** | | ***Atopobium*** | ***BF311*** | ***Bacteroides*** | | ***CF231*** | ***Fibrobacter*** | | ***Lactobacillus*** | | ***Leuconostoc*** |
| --- | --- | --- | --- | --- | --- | --- | --- | --- | --- | --- | --- | --- | --- | --- | --- |
| **CH_4_ yield (g/kg DMI)** | **0.31** | | | **0.41** | | -0.19 | 0.23 | 0.10 | | 0.26 | -0.03 | | -0.03 | | -0.03 |
| **Acetate: Propionate** | 0.08 | | | -0.08 | | **0.39** | 0.02 | 0.13 | | -0.22 | -0.46 | | 0.25 | | 0.18 |
| **Isobutyrate** | **0.48** | | | 0.18 | | 0.24 | -0.05 | 0.33 | | 0.25 | -0.21 | | 0.02 | | 0.01 |
| **Isovalerate** | **0.38** | | | 0.29 | | -0.10 | -0.05 | 0.01 | | 0.25 | -0.01 | | -0.08 | | -0.13 |
| **Ruminal pH** | 0.03 | | | 0.05 | | -0.07 | 0.10 | -0.08 | | -0.25 | -0.24 | | 0.05 | | -0.04 |
| **NH_3_-N** | 0.52 | | | 0.25 | | 0.15 | -0.15 | 0.14 | | 0.28 | 0.03 | | -0.05 | | 0.08 |
| **Quorum Sensing** | 0.05 | | | -0.18 | | -0.14 | -0.04 | 0.30 | | 0.12 | 0.15 | | 0.21 | | 0.26 |
| **Methane metabolism** | **0.67** | | | **0.30** | | 0.13 | 0.09 | 0.26 | | 0.38 | -0.17 | | 0.06 | | 0.11 |
| **PPAR signalling** | -0.27 | | | 0.02 | | -0.46 | 0.25 | 0.07 | | 0.35 | 0.28 | | 0.09 | | 0.12 |
| **Pyruvate metabolism** | 0.28 | | | -0.01 | | -0.03 | 0.08 | 0.27 | | 0.26 | 0.00 | | 0.04 | | 0.14 |
| **Purine metabolism** | **-0.36** | | | -0.30 | | -0.09 | -0.17 | -0.08 | | -0.29 | 0.13 | | 0.03 | | -0.10 |
| **C_5_ branched dibasic acid metabolism** | **0.54** | | | **0.40** | | -0.04 | 0.14 | 0.17 | | 0.40 | -0.28 | | -0.07 | | -0.04 |
|  | | ***Weissella*** | ***Ruminococcus*** | | ***Anaerovibrio*** | | ***Selenomonas*** | | ***Succiniclasticum*** | | ***Bulleidia*** | ***p-75-a5*** | | ***Archaea: Bacteria*** | |
| **CH_4_ yield (g/kg DMI)** | | 0.18 | 0.03 | | 0.20 | | 0.30 | | 0.13 | | -0.23 | 0.07 | | **0.33** | |
| **Acetate: Propionate** | | 0.10 | 0.02 | | **-0.51** | | **-0.59** | | **-0.25** | | 0.35 | -0.19 | | 0.10 | |
| **Isobutyrate** | | -0.08 | 0.32 | | -0.11 | | 0.08 | | -0.05 | | -0.15 | 0.09 | | 0.47 | |
| **Isovalerate** | | -0.24 | -0.05 | | 0.33 | | 0.61 | | 0.15 | | -0.52 | 0.23 | | 0.37 | |
| **Ruminal pH** | | 0.12 | 0.08 | | -0.21 | | -0.21 | | -0.05 | | 0.37 | -0.23 | | 0.05 | |
| **NH_3_-N** | | -0.05 | 0.08 | | 0.23 | | 0.38 | | 0.08 | | -0.25 | 0.49 | | 0.50 | |
| **Quorum Sensing** | | 0.25 | **0.58** | | 0.08 | | 0.04 | | 0.09 | | -0.30 | 0.13 | | 0.04 | |
| **Methane metabolism** | | 0.15 | **0.35** | | -0.02 | | 0.05 | | -0.25 | | -0.21 | 0.29 | | **0.67** | |
| **PPAR signalling** | | 0.16 | 0.41 | | 0.28 | | 0.26 | | 0.09 | | -0.49 | 0.01 | | -0.27 | |
| **Pyruvate metabolism** | | 0.25 | **0.77** | | -0.05 | | -0.07 | | -0.13 | | -0.19 | 0.20 | | 0.30 | |
| **Purine metabolism** | | -0.18 | -0.14 | | -0.03 | | -0.09 | | 0.26 | | 0.02 | -0.25 | | **-0.38** | |
| **C_5_ branched dibasic acid metabolism** | | -0.02 | 0.24 | | 0.06 | | 0.19 | | -0.11 | | -0.14 | 0.15 | | **0.56** | |

| **(B) Kendall's P- Value** | ***Methanobrevibacter*** | ***VadinCA11*** | ***Atopobium*** | ***BF311*** | | ***Bacteroides*** | | ***CF231*** | ***Fibrobacter*** | | | ***Lactobacillus*** | | ***Leuconostoc*** |
| --- | --- | --- | --- | --- | --- | --- | --- | --- | --- | --- | --- | --- | --- | --- |
| **CH_4_ yield (g/kg DMI)** | **0.094** | **0.025** | 0.294 | 0.222 | | 0.586 | | 0.161 | 0.856 | | | 0.892 | | 0.852 |
| **Acetate: Propionate** | 0.653 | 0.650 | **0.037** | 0.928 | | 0.498 | | 0.242 | **0.013** | | | 0.177 | | 0.330 |
| **Isobutyrate** | **0.009** | 0.318 | 0.204 | 0.787 | | **0.078** | | 0.177 | 0.258 | | | 0.928 | | 0.963 |
| **Isovalerate** | **0.038** | 0.123 | 0.586 | 0.787 | | 0.964 | | 0.177 | 0.964 | | | 0.653 | | 0.486 |
| **Ruminal pH** | 0.856 | 0.784 | 0.714 | 0.586 | | 0.682 | | 0.173 | 0.186 | | | 0.785 | | 0.815 |
| **NH_3_-N** | **0.005** | 0.173 | 0.414 | 0.418 | | 0.442 | | 0.126 | 0.892 | | | 0.787 | | 0.676 |
| **Quorum Sensing** | 0.776 | 0.338 | 0.444 | 0.849 | | 0.105 | | 0.506 | 0.418 | | | 0.255 | | 0.156 |
| **Methane metabolism** | **<0.001** | **0.094** | 0.487 | 0.613 | | 0.166 | | **0.038** | 0.356 | | | 0.748 | | 0.570 |
| **PPAR signalling** | 0.144 | 0.913 | **0.012** | 0.176 | | 0.704 | | **0.058** | 0.128 | | | 0.626 | | 0.503 |
| **Pyruvate metabolism** | 0.132 | 0.963 | 0.890 | 0.682 | | 0.143 | | 0.158 | 1.000 | | | 0.820 | | 0.452 |
| **Purine metabolism** | **0.050** | 0.110 | 0.638 | 0.351 | | 0.674 | | 0.113 | 0.483 | | | 0.852 | | 0.597 |
| **C_5_ branched dibasic acid metabolism** | **0.003** | **0.030** | 0.844 | 0.435 | | 0.351 | | **0.032** | 0.128 | | | 0.696 | | 0.840 |
|  | ***Weissella*** | ***Ruminococcus*** | ***Anaerovibrio*** | | ***Selenomonas*** | | ***Succiniclasticum*** | | | ***Bulleidia*** | ***p-75-a5*** | | ***Archaea: Bacteria*** | |
| **CH_4_ yield (g/kg DMI)** | 0.338 | 0.892 | 0.276 | | 0.102 | | 0.498 | | | 0.222 | 0.716 | | **0.078** | |
| **Acetate: Propionate** | 0.586 | 0.928 | **0.006** | | **0.001** | | **0.077** | | | **0.059** | 0.298 | | 0.589 | |
| **Isobutyrate** | 0.650 | **0.087** | 0.557 | | 0.684 | | 0.787 | | | 0.418 | 0.619 | | **0.012** | |
| **Isovalerate** | 0.204 | 0.787 | **0.078** | | **0.001** | | 0.418 | | | **0.005** | 0.222 | | **0.048** | |
| **Ruminal pH** | 0.522 | 0.650 | 0.254 | | 0.254 | | 0.785 | | | **0.046** | 0.218 | | 0.785 | |
| **NH_3_-N** | 0.785 | 0.653 | 0.222 | | **0.042** | | 0.653 | | | 0.177 | **0.008** | | **0.007** | |
| **Quorum Sensing** | 0.180 | **0.002** | 0.668 | | 0.812 | | 0.635 | | | 0.107 | 0.475 | | 0.849 | |
| **Methane metabolism** | 0.430 | **0.059** | 0.926 | | 0.782 | | 0.182 | | | 0.250 | 0.116 | | **<0.001** | |
| **PPAR signalling** | 0.382 | **0.026** | 0.128 | | 0.158 | | 0.626 | | | **0.008** | 0.957 | | 0.144 | |
| **Pyruvate metabolism** | 0.182 | **<0.001** | 0.784 | | 0.714 | | 0.494 | | | 0.294 | 0.272 | | 0.111 | |
| **Purine metabolism** | 0.324 | 0.456 | 0.888 | | 0.640 | | 0.162 | | | 0.926 | 0.175 | | **0.040** | |
| **C_5_ branched dibasic acid metabolism** | 0.922 | 0.204 | 0.731 | | 0.303 | | 0.558 | | | 0.435 | 0.404 | | **0.002** | |

**Supplementary Table S8. Associations between** CH_4_ **yield, metabolic pathways and metabolites observed in the rumen of the steers. (n = 16 observations).** Values in upper triangle indicates the Kendall’s P value.

|  | **CH_4_ yield (g/kg DMI)** | | **A:P** | **Isobutyrate** | **Isovalerate** | | **Ruminal pH** | | **NH_3_-N** | **Quorum Sensing** | **CH_4_ metabolism** | |
| --- | --- | --- | --- | --- | --- | --- | --- | --- | --- | --- | --- | --- |
| **CH_4_ yield (g/kg DMI)** |  | | 0.190 | 0.222 | 0.390 | | 0.494 | | 0.222 | 0.886 | **0.021** | |
| **Acetate: Propionate** | -0.24 | |  | 0.207 | 0.207 | | 0.238 | | 0.207 | 0.924 | 0.613 | |
| **Isobutyrate** | 0.23 | | 0.23 |  | **0.019** | | 0.586 | | 0.150 | **0.088** | **0.004** | |
| **Isovalerate** | 0.16 | | -0.23 | 0.43 |  | | **0.084** | | **0.031** | 0.155 | 0.250 | |
| **Ruminal pH** | 0.13 | | 0.22 | 0.10 | -0.32 | |  | | 0.238 | 0.811 | 0.286 | |
| **NH_3_-N** | 0.23 | | -0.23 | 0.27 | **0.40** | | -0.22 | |  | 1.000 | **0.048** | |
| **Quorum Sensing** | -0.03 | | 0.02 | 0.32 | 0.26 | | -0.04 | | 0.00 |  | 0.265 | |
| **Methane metabolism** | **0.43** | | 0.09 | 0.52 | 0.21 | | 0.20 | | 0.37 | 0.21 |  | |
| **PPAR signalling** | -0.04 | | -0.29 | 0.05 | 0.25 | | -0.20 | | -0.17 | 0.61 | -0.12 | |
| **Pyruvate metabolism** | 0.12 | | 0.06 | 0.40 | 0.04 | | 0.23 | | 0.14 | 0.56 | **0.55** | |
| **Purine metabolism** | **-0.42** | | 0.02 | -0.03 | 0.10 | | -0.12 | | -0.36 | 0.25 | **-0.47** | |
| **C_5_ branched dibasic acid metabolism** | **0.47** | | 0.04 | **0.54** | **0.31** | | 0.28 | | 0.36 | 0.08 | **0.75** | |
|  | | **PPAR signalling** | **Pyruvate metabolism** | | | **Purine metabolism** | | **C_5_ branched dibasic acid metabolism** | | | |  |
| **CH_4_ yield (g/kg DMI)** | | 0.828 | 0.522 | | | **0.022** | | **0.011** | | | |  |
| **Acetate: Propionate** | | 0.116 | 0.750 | | | 0.926 | | 0.845 | | | |  |
| **Isobutyrate** | | 0.787 | **0.032** | | | 0.852 | | **0.003** | | | |  |
| **Isovalerate** | | 0.176 | 0.820 | | | 0.576 | | **0.097** | | | |  |
| **Ruminal pH** | | 0.275 | 0.214 | | | 0.511 | | 0.127 | | | |  |
| **NH_3_-N** | | 0.357 | 0.438 | | | **0.050** | | **0.051** | | | |  |
| **Quorum Sensing** | | **0.001** | **0.002** | | | 0.185 | | 0.680 | | | |  |
| **Methane metabolism** | | 0.507 | **0.003** | | | **0.012** | | **<0.001** | | | |  |
| **PPAR signalling** | |  | 0.100 | | | **0.082** | | 0.639 | | | |  |
| **Pyruvate metabolism** | | 0.30 |  | | | 0.238 | | **0.009** | | | |  |
| **Purine metabolism** | | 0.32 | -0.22 | | |  | | **0.007** | | | |  |
| **C_5_ branched dibasic acid metabolism** | | -0.09 | 0.49 | | | -0.50 | |  | | | |  |


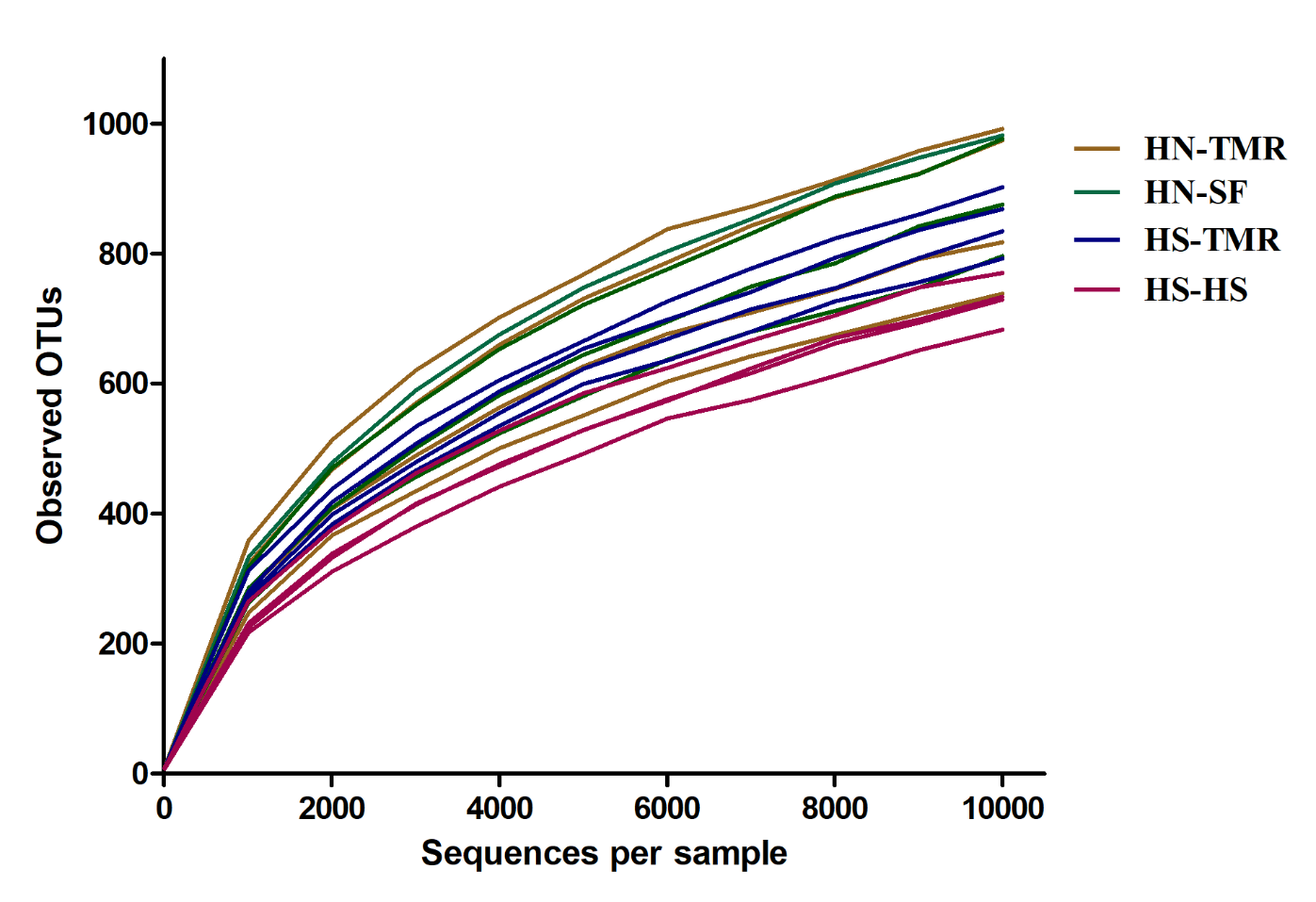


**Supplementary Figure S1. Rarefaction curve showing sequencing depth of the V4 domain of 16srDNA of rumen microbes from Hanwoo (HN) and Holstein (HS) steers fed by two different systems.**


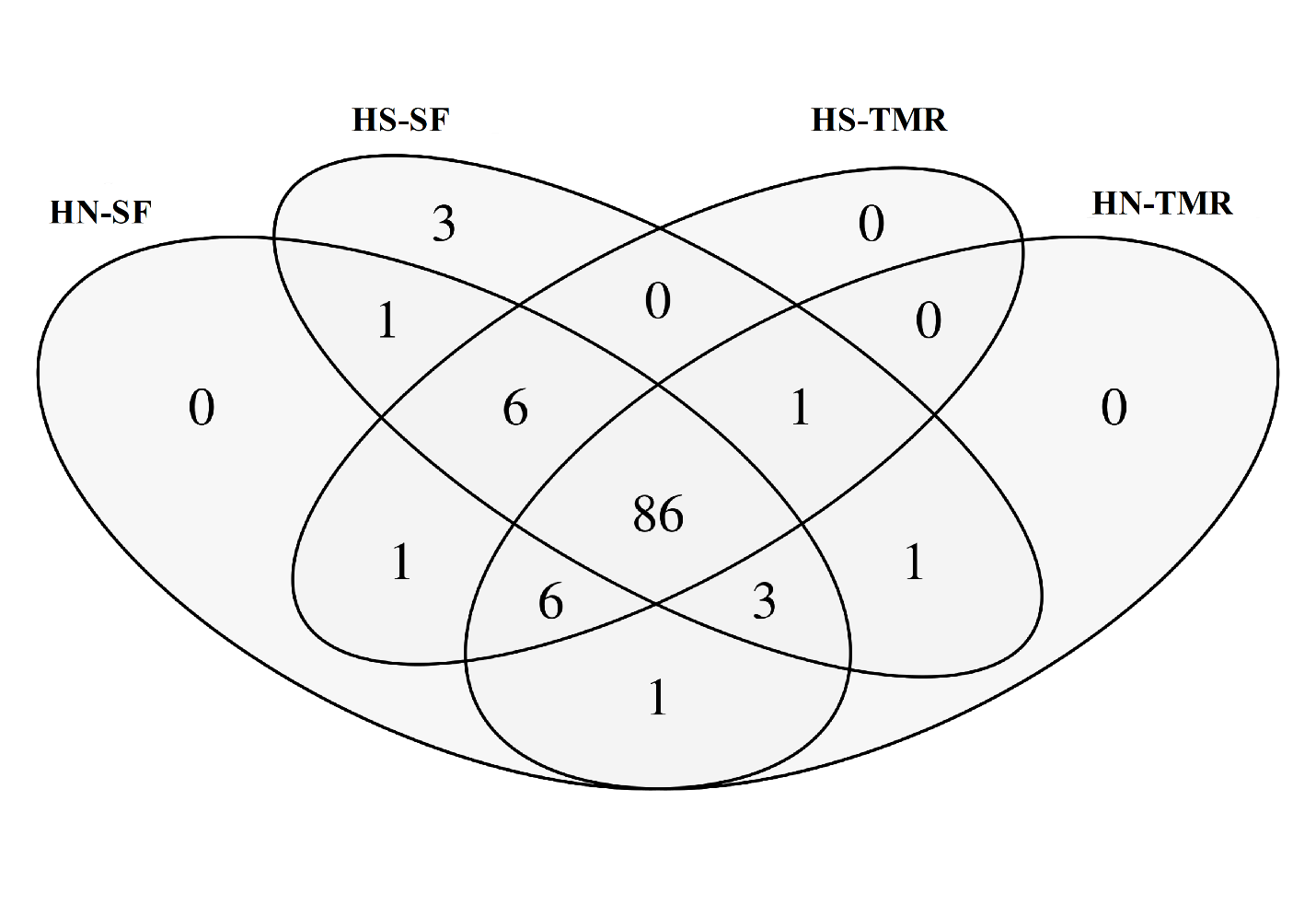


**Supplementary Figure S2. Venn diagram of the core microbiomes of rumen varied by host genetics and feeding system.**

The 4 circle Venn diagram shows the rumen core microbiomes as determined by only those taxa which were ubiquitous for all steers. Each circle represents a breed fed by a feeding system, bacterial taxa within overlapping areas were common to the corresponding group.


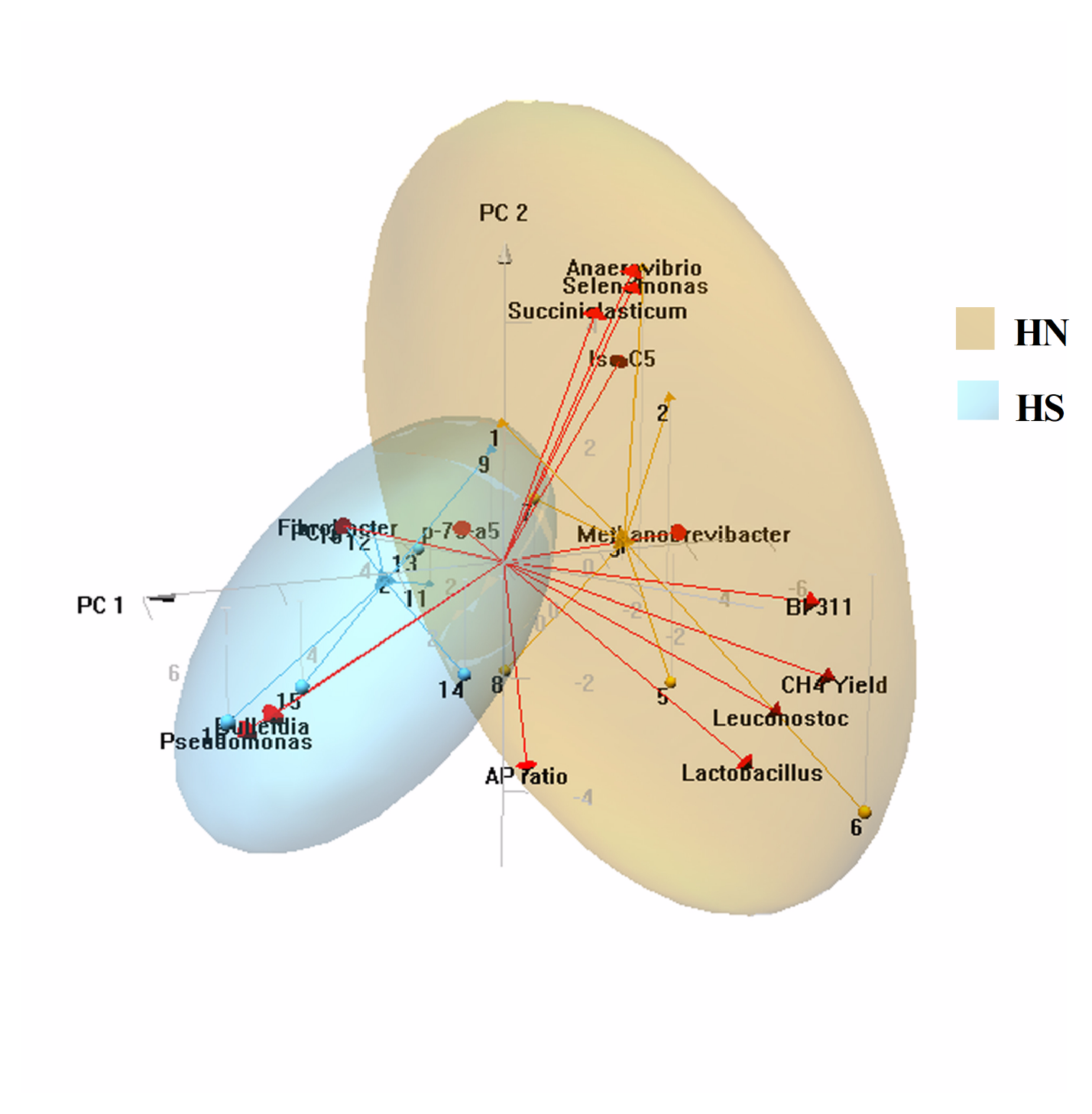


**Supplementary Figure S3. Principal component analysis (PCA) displaying correlations among the rumen bacterial communities, CH_4_ yield and fermentation parameters of Hanwoo (HN) and Holstein (HS) steers fed by TMR ( ) or SF (●) system.**
